# Enhanced WWP2 Exacerbates Podocyte Injury in Lupus Nephritis through Monoubiquitylating H2A at K119

**DOI:** 10.7150/ijbs.131475

**Published:** 2026-07-22

**Authors:** Ran You, Yuteng Jiang, Yingyi Xu, Qianying Liu, Menglei Gu, Yue Zhang, Zhanjun Jia, Mengqiu Wu, Mi Bai, Aihua Zhang

**Affiliations:** 1 Nanjing Key Laboratory of Pediatrics, Children's Hospital of Nanjing Medical University, Nanjing, China.; 2 Jiangsu Key Laboratory of Pediatrics, Nanjing Medical University, Nanjing, China.; 3 Institute of Nephrology, Zhong Da Hospital, Southeast University School of Medicine, Nanjing, China.

**Keywords:** H2A, lupus nephritis, podocyte injury, ubiquitylation, WWP2

## Abstract

Podocyte injury drives proteinuria in lupus nephritis (LN). Targeting therapy against podocyte injury in LN is in demand. The E3 ubiquitin ligase WWP2 has context-dependent roles in renal tubulointerstitial injury and repair; however, since immune disorder-induced podocyte injury has distinct pathological foundations than tubulointerstitial pathology, WWP2's function in podocytes and LN remains unknown. Here we found that WWP2 protein levels significantly increased in the glomeruli of LN kidneys and were correlated with proteinuria in patients. Renal WWP2 knockdown alleviated, while its overexpression exacerbated proteinuria and podocyte injury in MRL/lpr mice. Podocyte-specific WWP2 deficiency protected against proteinuria in the NTS model. Mechanistically, multi-omics analysis revealed WWP2's association with systemic lupus erythematosus pathways and histone H2A. We identified WWP2 as a novel E3 ligase that monoubiquitylated H2A at K119 (ub-H2A). Ub-H2A levels increased in LN glomeruli, depended on WWP2, and mediated WWP2-aggravated podocyte injury. This effect involved alteration of actin dynamics via FAK signaling. Finally, a novel WWP2 inhibitor attenuated proteinuria in nephritic mice. In conclusion, the WWP2/ub-H2A axis promotes podocyte injury in LN by disrupting actin dynamics. Targeting this pathway represents a potential therapeutic strategy for LN.

## Introduction

Systemic lupus erythematosus (SLE) is a clinically common autoimmune syndrome that affects millions of patients globally 1. Approximately one-third of patients with SLE are complicated with lupus nephritis (LN), and up to 20% of these patients may develop end-stage renal disease within 10 years of SLE diagnosis. 2. LN almost doubles the standard mortality ratio in Asian patients with SLE 3. The clinically available therapy for LN is mostly limited to immunosuppressive therapy, with often unsatisfactory effectiveness in renal protection 2, 4. Hence renal protective therapy for patients with LN is in demand. Among the broad spectrum of renal injuries, LN is characterized by glomerular lesions that result from a dramatic inflammatory cascade. This process is initiated by immune complex (IC) deposition and further amplified by pro-inflammatory cytokines. Podocytes maintain the structural and functional integrity of the glomerular filtration barrier by anchoring to the glomerular basement membrane and forming the slit diaphragm with the adjacent podocytes through their foot processes 4-6. Inflammation-induced podocyte injury, manifested as foot process effacement, podocyte-specific markers diminish, and even cell death, is a key pathological factor contributing to proteinuria in LN 5-7. Substantial evidence indicates that podocytes are direct targets of LN 5, 6, and podocyte loss may indicate poor prognosis in patients with LN 4. Therefore, protecting podocytes represents a promising therapeutic strategy for LN 6. Enhancing the understanding of the molecular mechanisms driving podocyte injury may facilitate the development of novel targeted pharmacotherapies.

Protein ubiquitylation facilitated by E3 ligase regulate cellular signaling transduction via modulating the protein stability, activity, or interaction mode of the substrate proteins in a rapid and reversible manner 8. Accumulating evidence suggested that dysregulation of ubiquitylation contribute to immune disorders, including autoimmunity such as SLE and its comorbidity 8, 9. The WW domain containing E3 ubiquitin protein ligase 2 (WWP2) is an E3 ubiquitin ligase belonging to the Nedd4 family 10. Constructed with a catalytic HECT domain in its C terminal, WWP2 can recognize and directly ubiquitylate its substrate in the nucleus or cytoplasm during various cellular events 11, 12. WWP2 has multiple pathophysiological roles including promoting oncogenesis 10, 13, 14, exacerbating cardiac fibrosis 11, 15, participating in endothelial injury in hypertension 16, and modulating the immune response as a co-effector 17. We recently reported the essential role of WWP2 in tubulointerstitial injury and repair, demonstrating that WWP2 may antagonize acute kidney injury by protecting against acute tubular injury and exacerbate renal fibrosis by modulating the differentiation of renal fibroblasts and maladaptation of tubular epithelial cells post ischemic insults 18-20. Therefore, WWP2 may regulate renal pathology in a context-dependent manner. It remains unclear whether WWP2 is implicated in podocyte injury and proteinuria in LN.

On the other hand, epigenetic modifications including histone acetylation have been shown to modulate LN progression in mouse models 21. However, the role of histone ubiquitylation in LN progression remains unclear^22^. Monoubiquitylation of H2A at the K119 site (ub-H2A) is evolutionarily conserved, accounting for 5-15% of the total H2A in higher eukaryotes 23, 24. Ub-H2A maintains nucleosome stability by impeding DNA detachment 24. It is broadly distributed throughout the whole genome, and its increased binding to the promoter region may repress gene transcription, whereas decreased binding may promote gene transcription 23, 25. Physiologically, ub-H2A is a fundamental regulator for the maintenance of cellular identity and proper differentiation via silencing key developmental genes such as Homebox genes during embryogenesis under the regulation of RING1A/B, the well-established E3 ligase for ub-H2A production 25. The pathological functions of ub-H2A in the post-developmental stages and its regulatory mechanisms remain largely unexplored 24. Recent studies indicated that ub-H2A may be involved in pathological conditions such as DNA damage response 26 and glucose metabolism in cancer cells 27. The pathological role of ub-H2A in LN requires further investigation.

Here, we established a novel functional role of WWP2/ub-H2A in aggravating proteinuria and podocyte injury in LN. We propose the use of a novel WWP2 inhibitor as a potential therapeutic strategy against LN.

## Materials and Methods

### Ethics approval

The protocol for using patient samples and clinical data was approved by the Ethics Committee of the Children's Hospital of Nanjing Medical University (Approval No. 202304069-1). Informed consent was provided by all participating patients and their parents. The protocol for the animal study was approved by the IACUC at Nanjing Medical University (Approval No. 2007001-3). A detailed description of the methods and materials can be found in **Supplemental materials**.

### Statistical analysis

The data were normalized to control groups unless otherwise described, tested for normal distribution before being analysis with the Student's *t*-test or analysis of variance (ANOVA) if fitted normal distribution, or, otherwise, with non-parametric Mann-Whitney test. The statistical analysis was performed using GraphPad Prism software (version 9.0; GraphPad Software, Inc.). The data are presented in bar charts as mean + Standard Error of the Mean (SEM). P <0.05 was considered statistically significant.

## Results

### WWP2 Increased in the Glomeruli of Patients with LN and MRL/lpr Mice

**Figure 1:**
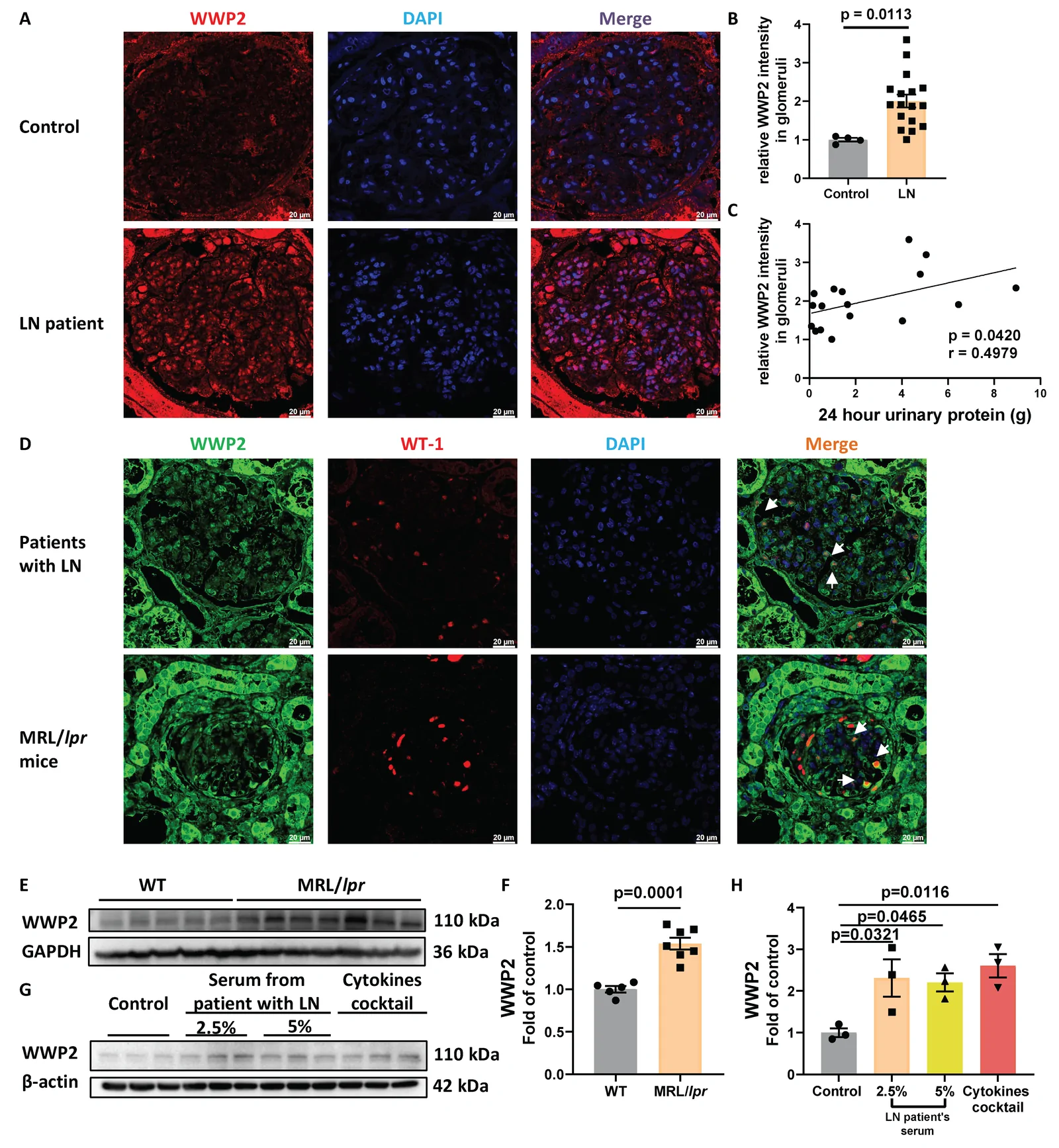
** WWP2 Increased in the Glomeruli of Patients with LN and MRL/lpr Mice.** We included 17 LN patients and 4 age- and gender-matched controls to study the clinical correlation of WWP2 with the severity of proteinuria in LN patients. Immunofluorescent staining images were used to measure glomerular WWP2 in patients' kidneys. WWP2's expression in the glomeruli of 18-week-old female MRL/lpr mice and their age- and gender-matched genetic background control MRL/MPJ mice were examined via immunohistochemical analysis. Immortalized human podocyte cell lines, HPC, were stimulated with serum from an LN patient at various concentrations or with a cytokines cocktail and WWP2 expression was studied with Western blotting analysis. (**A-C**) WWP2 was significantly upregulated in the glomeruli in the LN patients and correlated with patients' 24-hour proteinuria. **A**: Representative images of WWP2 immunofluorescent staining in glomeruli from LN patients or controls. Red: WWP2; Blue: DAPI. n=4 or 17 in Control or LN group, respectively. Scale bar: 20 μm.** B-C**: glomerular WWP2 was significantly upregulated in LN group comparing to controls (**B**) and correlated with LN patients' 24h proteinuria (**C**). (**D**) Representative images indicating that WWP2 localized in podocytes whose nuclei were indicated by its nuclear marker, WT-1 and DAPI in the glomeruli of LN patients (**top row**) and MRL/lpr mice (**bottom row**). Green: WWP2; Red: WT-1; Blue: DAPI. Scale bar: 20 μm. (**E-F**) WWP2 was significantly increased in kidneys of MRL/lpr mice by Western blotting analysis. n=5 or 7 for the Control or MRL/lpr groups, respectively. (**G-H**) WWP2 was significantly increased in the podocytes exposed to the LN patient's serum or cytokines cocktail. n=3 biological replicates. Bar charts are presented as mean + SEM. P-values for two- or multiple-group comparisons were calculated by Two-tailed unpaired Student's *t*-test or One-way ANOVA followed by Dunnett's post-hoc test, respectively.

To study the clinical correlation between WWP2 abundance and LN, we enrolled 17 patients with LN with varying severities of proteinuria, as indicated by the 24-h proteinuria results (**Table 1**). WWP2 significantly increased in the glomeruli of patients with LN (**Figure 1A-B**). Moreover, glomerular WWP2 abundance was significantly correlated with proteinuria (**Figure 1C**). Podocyte injury is an essential pathological event underlying proteinuria 6. We found an evident colocalization of WWP2 in podocyte which was indicated by Wilms' Tumor-1 (WT-1), a podocyte's nuclear marker 28, in the glomeruli of patients with LN and MRL/lpr mice, a well-known spontaneous SLE mouse model 29 (**Figure 1D**). WWP2 expression was significantly increased in the kidneys of MRL/lpr mice (**Figure 1E-F**). Pro-inflammatory cytokines augment glomerular inflammation, leading to podocyte injury during LN 6. Hence, exposing podocyte cultures to a cytokine cocktail comprising interleukin-1β, tumor necrosis factor-α, interferon-α, and interferon-γ may mimic podocyte injury during LN *in vitro* 7. WWP2 was significantly upregulated in human podocyte culture (HPC) by the cytokine cocktail and patient serum (**Figure 1G-H**). Thus, elevated WWP2 levels in podocytes may be implicated in the progression of proteinuria and podocyte injury in LN.

### WWP2 Knockdown Alleviated Proteinuria and Podocyte Injury in the MRL/lpr mice

**Figure 2:**
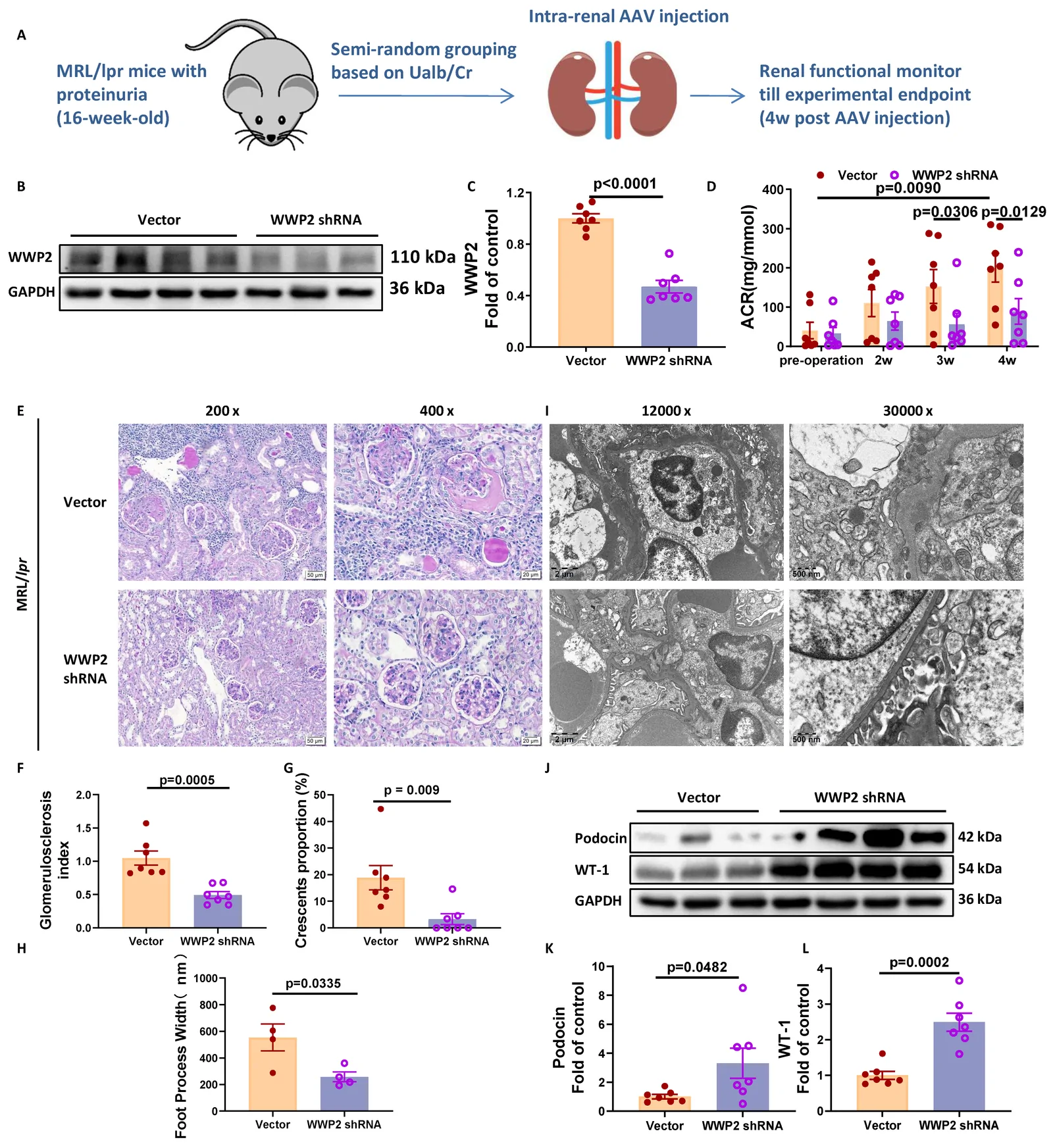
** WWP2 Knockdown Alleviated Proteinuria and Podocyte Injury in the MRL/lpr mice.** Sixteen-week-old MRL/lpr mice were injected with WWP2 shRNA AAV9 or vector AAV9 via intra-renal injection. (**A**) **Experimental scheme**: the mice were semi-randomly assigned to either group by their albumin/creatinine ratio in the instant urine (Ualb/Cr, ACR) collected within 3 days before injection. The kidney function of the mice was indicated by ACR before and 2, 3, and 4 weeks after the AAV injection. The mice were euthanized at the experimental endpoint, i.e., 4 weeks post AAV injection, for kidney samples for further analysis. n=7 for each group. (**B-C**) Knockdown efficacy in the kidneys was examined by Western blotting analysis on the kidney tissue collected 4 weeks post-injection. **B**: Representative blots; **C**: Bar chart for quantification. (**D**) WWP2 knockdown significantly dampened the progression of proteinuria in the lpr mice. (**E-G**) WWP2 knockdown attenuated the glomerular lesion as represented by mesenchymal proliferation, glomeruli sclerosis, and crescent formation in the kidneys of MRL/lpr mice.** E**: Representative images of PAS staining. Left: 200 X magnification, scale bars show 50 μm; right: 400 X magnification, scale bars show 20 μm. **F-G**: WWP2 knockdown significantly decreased glomerulosclerosis index (**F**) and crescent proportion (**G**) in the kidneys of MRL/lpr mice. (**H-I**) WWP2 knockdown alleviated podocyte injury as represented by loss of foot process in the kidneys of MRL/lpr mice. **H**: quantification of foot process width from TEM images. n=4. **I**: representative images for 12,000 x (left) and 30,000 x (right). Scale bars in the left and right panels represent 2 μm and 500 nm, respectively. (**J-L**) WWP2 knockdown significantly increased podocyte markers, such as podocin and WT-1 in the kidneys of MRL/lpr mice as examined by Western blotting analysis. **J**: Representative blots; **K-L**: Quantification results. Bar charts are presented as mean + SEM. P-values for two- or multiple-group comparisons were calculated by Two-tailed unpaired Student's *t*-test or Two-way ANOVA followed by Fisher's LSD post-hoc test, respectively.

To investigate the implication of WWP2 in LN, we manipulated WWP2 with adeno-associated virus (AAV) 2/9 30 in the kidneys of MRL/lpr mice, a spontaneous autoimmune strain that manifests progressive LN-like renal pathology and proteinuria 29. To minimize the fluctuation in experimental outcomes from individual differences in disease progression, we started the experiment following proteinuria onset in female MRL/lpr mice (16-month-old) and semi-randomly assigned them to two groups based on their albumin/creatinine ratio (ACR) in the instant urine samples collected 3d before AAV injection (**Figure 2A**). AAV2/9 successfully delivered genes to kidneys with chronic kidney disease (CKD) 30. We confirmed that intra-renal injection of WWP2 shRNA AAV2/9 significantly decreased WWP2 in the kidneys of MRL/lpr mice at the experimental endpoint, i.e, 4-week post-injection (**Figure 2B-C**).

We observed a time-dependent urinary ACR increase in MRL/lpr mice treated with the vector AAV, indicating progressive renal function decline (**Figure 2D**). WWP2 shRNA AAV dampened renal dysfunction progression by significantly delaying albuminuria progression and decreasing ACR levels at 3 and 4 weeks post-injection in MRL/lpr mice (**Figure 2D**). Consistent with the observed proteinuria, MRL/lpr mice in the vector group manifested severe glomerular lesions, including glomerulosclerosis and crescent formation, according to the histological examination via periodic acid-Schiff (PAS) staining. WWP2 knockdown significantly attenuated these glomeruli pathology, as shown by decreasing the glomerulosclerosis index and crescent proportion in the MRL/lpr mice kidneys (**Figure 2E-G**). Furthermore, WWP2 knockdown alleviated podocyte injury by attenuating foot process effacement, as quantified by foot process width in transmission electron microscopy (TEM) examination 4 (**Figure 2H-I**), and restoring the protein levels of podocyte markers, that is, podocin and WT-1, in MRL/lpr mouse kidneys (**Figure 2J-L**). These results demonstrated that WWP2 knockdown alleviated proteinuria and renal pathology, including podocyte injury, in the LN mouse model.

### WWP2 Exacerbated Proteinuria and Podocyte Injury in MRL/lpr Mice

We overexpressed WWP2 by injecting WWP2 AAV2/9 into the kidneys of 14-month-old MRL/lpr mice (**Figure S1A**). The vector was constructed with WWP2 cloned upstream of the mNeonGreen coding sequence in a non-fused manner. Compared with naïve controls, which did not receive AAV injection, both groups injected with AAV showed prominent and similar expression of mNeonGreen in the kidneys (**Figure S1B-C**). WWP2 AAV significantly increased WWP2 protein levels in the kidneys (**Figure S1D-E**). These data demonstrate the successful expression of exogenous genes, including WWP2 in the kidneys following AAV2/9 injection.

WWP2 significantly aggravated proteinuria (**Figure S1F**), glomerular lesions (**Figure S1G-I**) and podocyte injury, as shown by the more severe foot process effacement in the TEM examination and decreased podocyte markers, i.e., WT-1 and podocin (**Figure S1J-N**), in the kidneys of MRL/lpr mice. These data demonstrated that WWP2 overexpression aggravated proteinuria and worsened glomerular lesions, especially podocyte injury, in the LN mice kidneys.

Tubulointerstitial injury may be secondary to proteinuria and glomerular lesions in LN kidneys. We found that the effect of WWP2 manipulation on tubulointerstitial injury was consistent with that of glomerular lesions in the MRL/lpr mice (**Figure S2**), supporting the pathological role of WWP2 in LN glomerulonephritis.

### WWP2 Deficiency in Podocyte Prevented Proteinuria in Nephrotoxic Serum Nephritis Mouse Model

**Figure 3:**
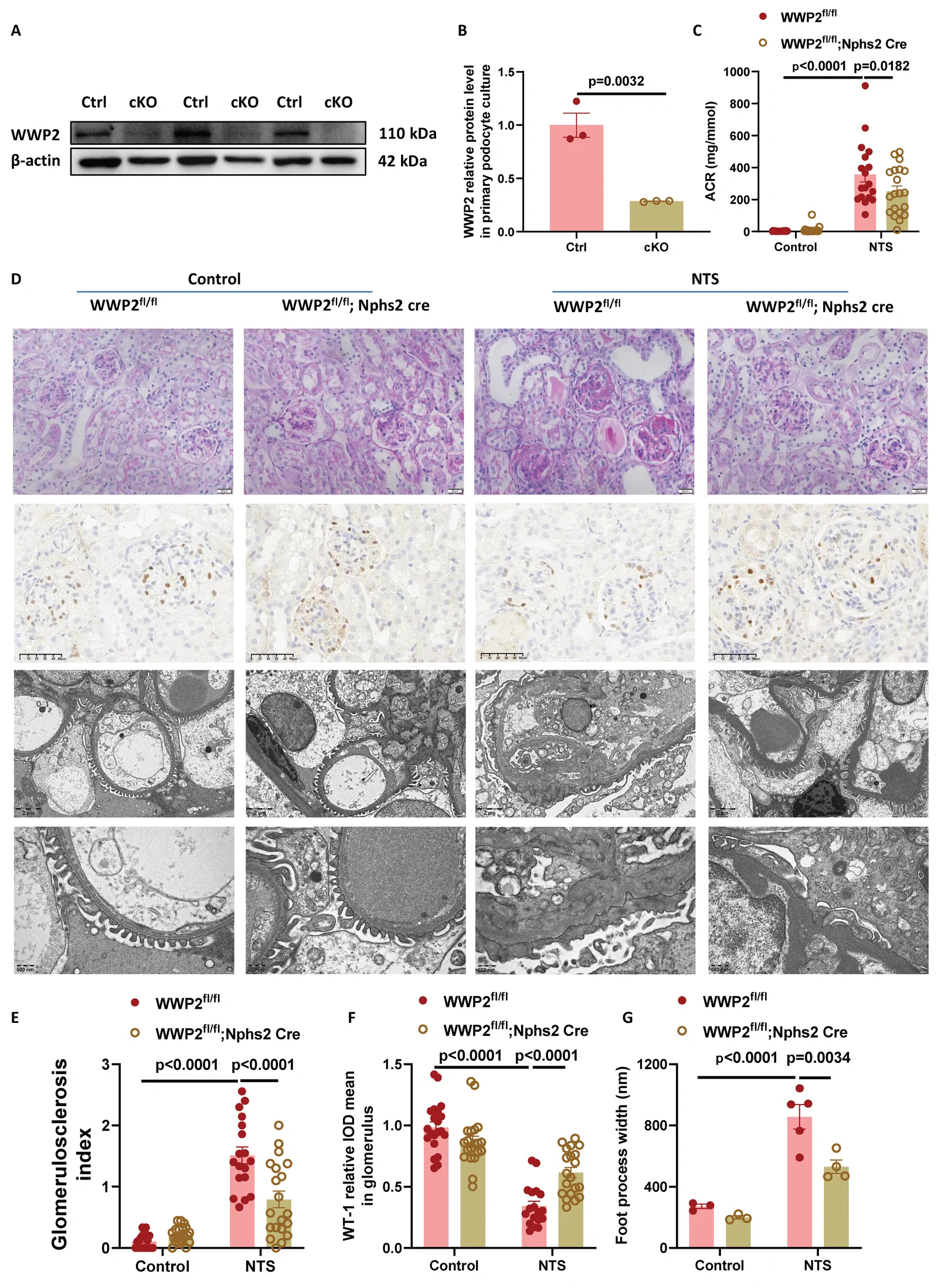
** WWP2 Deficiency in Podocyte Prevented Proteinuria in NTS Nephritis Mouse Model.** Eight-week-old male WWP2 podocyte-specific knockout mice (cKO, *Wwp2^flox/flox^*; *Nphs2-Cre*) mice and the littermate control (*Wwp2^flox/flox^* mice) were injected with 18 ul/g body weight sheep anti-rat GBM serum (Nephrotoxic serum) via intraperitoneal injection to establish the NTS model. Equal dosage of normal sheep serum was used as control. Four weeks post modeling, the mice were sampled for their instant urine within 3 days before euthanize for sample harvesting of serum and kidneys for further analysis. n=21, 20, 18, 20 for *Wwp2^fl/fl^*, cKO,* Wwp2^fl/fl^*+NTS, cKO+NTS, respectively. (**A-B**) WWP2 knockout efficacy examined by Western blotting analysis on the primary culture of mouse podocytes. n = 3. (**C**) WWP2 cKO significantly alleviated proteinuria as shown by the ACR in the NTS mice. Bar charts were plotted by ACR quantified from the instant urine samples 4-week post NTS injection. (**D-G**) WWP2 cKO significantly alleviated glomerular sclerosis and protected against podocyte injury in the NTS model. **D**: representative images of PAS staining (first row, scale bar: 20 μm), immunostaining of WT-1 (second row, scale bar: 50 μm), and TEM (third and bottom row, scale bars: 2 μm and 500 nm, respectively). **E**: glomerulosclerosis index scored from PAS staining; **F**: relative intensity of WT-1, a transcriptional factor mastering podocyte-specific gene expression located in podocytes' nuclei 28, in the glomeruli. **G**: foot process quantified from TEM images, n= 3, 3, 5, 4 for *Wwp2^fl/fl^*, cKO,* Wwp2^fl/fl^*+NTS, cKO+NTS, respectively; Bar charts are shown as mean ± SEM. P-values for **B** was calculated by Two-tailed unpaired *t*-test; that for others were by One-way ANOVA followed by Sidak's multiple comparisons test.

We bred a podocyte-specific WWP2 knockout mouse strain (*Wwp2*^fl/fl^; *Nphs2* cre+, WWP2 cKO), the knockout efficiency of which was verified in the primary culture of podocytes via western blotting (**Figure 3A-B**). In WWP2 cKO and control mice, we established a nephrotoxic serum (NTS) model, which develops glomerulonephritis through triggering the production of multiple antigens. This model manifests as proteinuria and podocyte injury, as indicated by reduced WT-1 expression in immunohistochemical staining 31, 32. WWP2 cKO significantly alleviated proteinuria (**Figure 3C**), glomerulosclerosis (**Figure 3D-E**), and podocyte injury in the NTS model, as shown by a significant restoration of WT-1 protein levels (**Figure 3D&F**) and reduction in foot process width in TEM examination (**Figure 3D&G**). These findings from podocyte-specific WWP2 knockout mice with induced nephritis further supported that WWP2 deficiency prevented proteinuria and glomerular pathology in nephritis, possibly by protecting podocytes.

### WWP2 Knockout Attenuated Podocyte Injury Under Pro-inflammatory Stimulation

**Figure 4:**
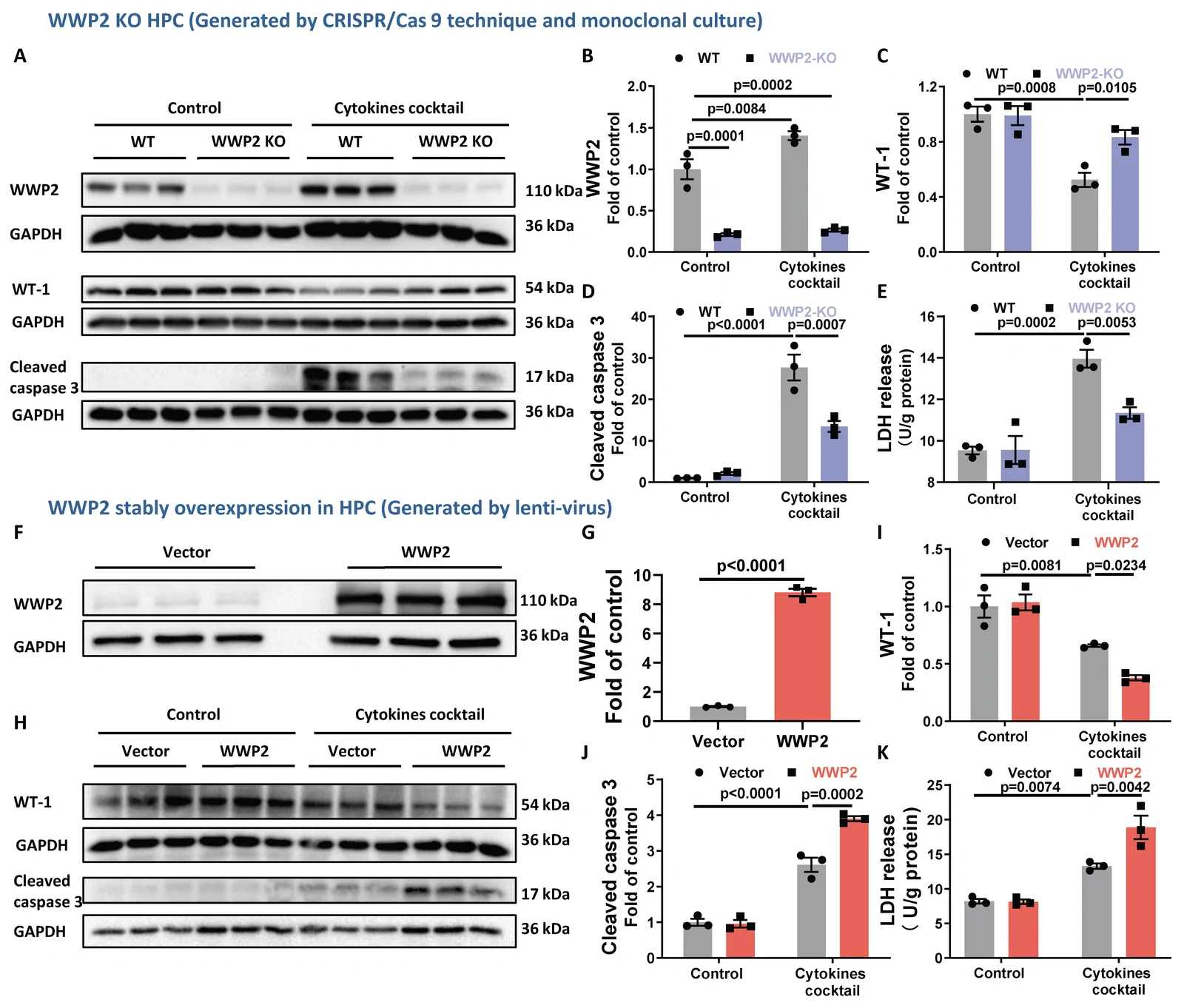
** WWP2 Knockdown Prevented Podocyte Injury under Pro-inflammatory Stimulation.** WWP2 KO HPC cell line was generated by transfecting sgRNA targeting the human WWP2 gene in HPC culture and screening for knockout monoclonal cells after eliminating the non-transfected cells with puromycin. WWP2 stably overexpression HPC cell line was generated by infecting the cells with WWP2 lentivirus and eliminating the non-transfected ones with puromycin. The control cells for WWP2 KO or stably overexpression HPCs (WT or Vector, respectively) were generated in parallel and cultured at similar conditions. The cell cultures were exposed to a cytokines cocktail for 24 hours before being harvested for supernatant and cell homogenates for further analysis. n=3 biological replicates. (**A-E**) WWP2 deficiency (**A&B**) significantly alleviated podocyte injury induced by pro-inflammatory cytokines stimulation, as shown by a restoration of WT-1 protein level (**A&C**) and a reduction in cleaved caspase-3 (**A&D**) and LDH release (**E**) in the cytokines cocktail-stimulated podocyte culture. (**F-K**) WWP2 overexpression (**F-G**) further decreased WT-1 (**H-I**) and increased cleaved caspase-3 (**H&J**) and LDH release (**K**) in the podocyte cultures stimulated by pro-inflammatory cytokines. Bar charts are presented as mean ± SEM. P-values for **G** was calculated by Two-tailed unpaired t-test; those for other charts were by One-way ANOVA followed by Sidak's multiple comparisons test.

To assess WWP2's effect on inflammatory podocyte injury, we exposed a WWP2 knockout HPC cell line to cytokine cocktail stimulation, an* in vitro* model for LN podocyte injury 7. Western blotting confirmed the knockout efficacy in the WWP2 knockout HPC and verified the upregulation of WWP2 by cytokine cocktail stimulation in WT HPC (**Figure 4A-B**). The cytokine cocktail significantly decreased WT-1 (**Figure 4A&C**), and induced cell death by increasing cleaved caspase-3 (**Figure 4A&D**) and lactate dehydrogenase (LDH) release (**Figure 4E**) in WT HPC. WWP2 knockout significantly increased WT-1 and decreased cleaved caspase-3 and LDH release in cytokine cocktail-stimulated HPC (**Figure 4A-E**). In addition, WWP2 overexpression (**Figure 4F-G**) significantly aggravated podocyte injury and cell death by decreasing WT-1 and increasing cleaved caspase-3 and LDH release in cytokine cocktail-stimulated podocytes (**Figure 4H-K**). These data from podocyte cultures demonstrated that WWP2 was upregulated in podocytes exposed to cytokines and may directly mediate inflammatory podocyte injury.

### Ub-H2A May Be a Potential Downstream Product of WWP2

**Figure 5:**
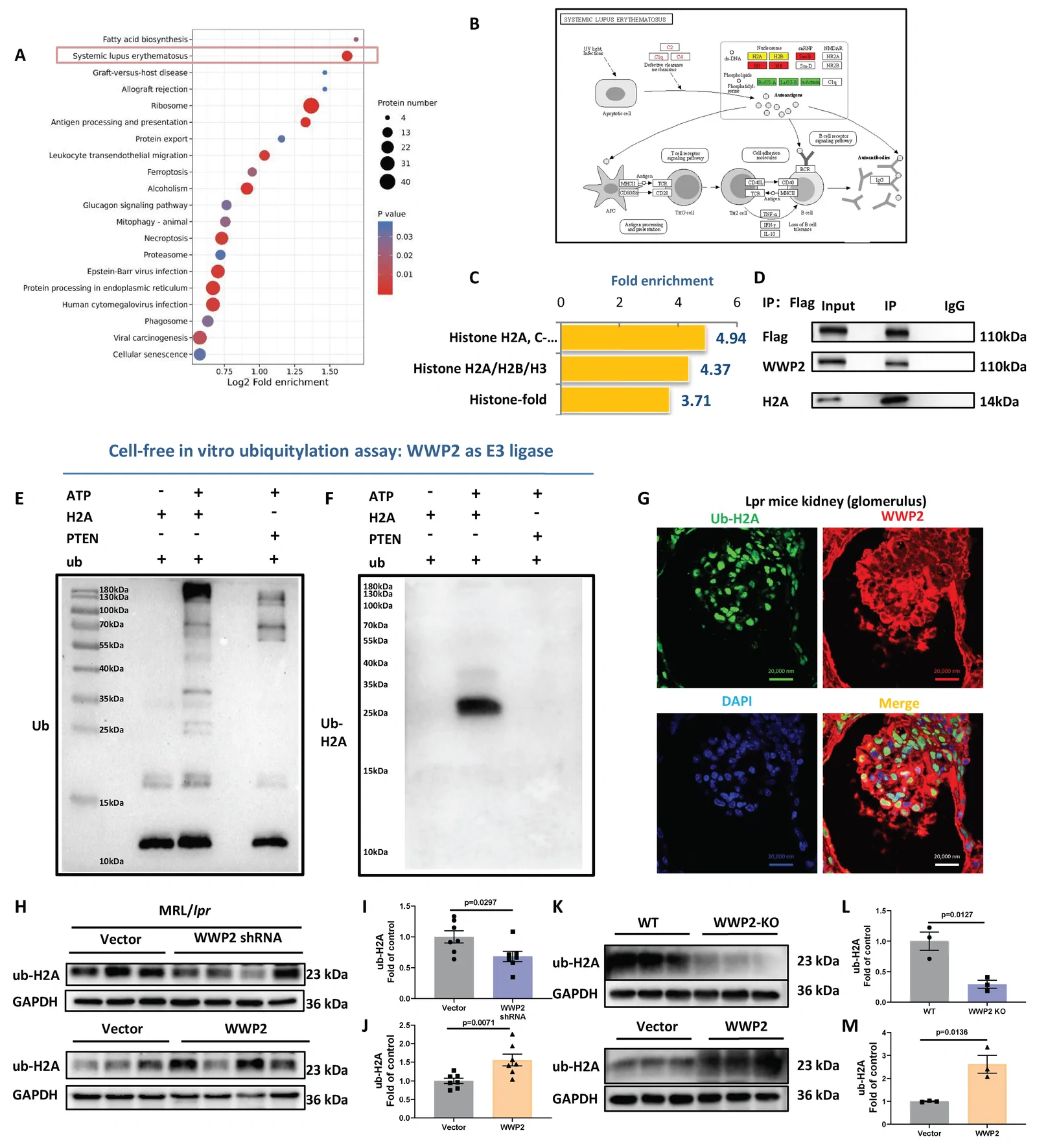
** Ub-H2A may be a Potential Downstream Product of WWP2.** Ubiquitylation omics analysis was performed on WWP2 overexpression tubular epithelial cells. (**A-B**) KEGG analysis on proteins with alteration in ubiquitylation in published datasets PXD048540 18. **A**: The analysis revealed a profound enrichment in immune-related pathways such as Systemic lupus erythematosus in the WWP2 overexpression cells. **B**: mapping of the proteins with altered ubiquitylation in SLE pathway. Red: up; Green: down; yellow: both up and down regulation in ubiquitylation. (**C**) GO analysis on the domains with upregulation in the WWP2 overexpression cells suggested H2A as one of the most profoundly affected candidates. (**D**) Co-IP assay results suggested a potential WWP2-H2A interaction. WWP2-Flag plasmid was transiently overexpressed in the HEK293T cells, harvested 24 hours post-transfection for co-IP analysis with Flag pre-coated beads. IgG-coated beads were used for negative control. (**E**) *In vitro* ubiquitylation assay results indicated that WWP2 may directly ubiquitylate H2A, generating mono- and poly-ubiquitylation products. The ubiquitylation reaction was performed in a cell-free system with WWP2 as E3 ligase and substrates, which was H2A or PTEN, a known substrate of WWP2 and hence could serve as a positive control. The negative control contains the same component as the reaction with H2A except without ATP, which fuels the ubiquitylation reaction. (**F**) The reaction products of WWP2-H2A *in vitro* ubiquitylation assay contain Ub-H2A (i.e., the monoubiquitylated H2A at the K119 site) according to ub-H2A monoclonal antibody detection. The blots in **E**,** F** showed the representative blots from 4 and 3 independent experiments, respectively. Original blots detecting the reaction component was provided as supplemental file. (**G**) WWP2 and ub-H2A may colocalize in the glomerular nuclei in the MRL/lpr mice kidneys, as shown by the immunofluorescent staining images. Green: ub-H2A; Red: WWP2; Blue: DAPI. (**H-M**) Ub-H2A protein level decreased WWP2 silencing and increased upon WWP2 overexpression in MRL/lpr mouse kidneys (**H-J**) and HPC (**K-M**). n=7 in animal studies and n=3 biological replicates in cell cultures. Bar charts are presented as mean ± SEM. Two-tailed unpaired Student's *t*-test were used to determine the P-values in **I-J** and **L-M**.

To elucidate the mechanism by which WWP2 influences LN, we analyzed the ubiquitination omics dataset of tubular epithelial cells overexpressing WWP2 18. The dataset included 646 upregulated ubiquitination sites on 522 proteins and 260 downregulated sites on 221 proteins 18. Kyoto Encyclopedia of Genes and Genomes (KEGG) analysis of ubiquitylation proteins showed that the SLE pathway, including histones H2A, H2B, and H3, was the second most significantly enriched pathway in WWP2 overexpression cells (**Figure 5A-B**). Gene Ontology (GO) analysis of the upregulated ubiquitylation domains highlighted H2A as a potential substrate for WWP2 (**Figure 5C**). Therefore, ubiquitylated H2A may be implicated in the pathological effects of WWP2 in LN. We first verified the potential WWP2-H2A interaction using co-immunoprecipitation (IP) assay (**Figure 5D**). Furthermore, *in vitro* ubiquitylation assays demonstrated that WWP2 could ubiquitylate H2A as its E3 ligase, possibly yielding products with mono- (∼25 kDa) and poly-ubiquitylation (25-110 kDa) (**Figure 5E**). Poly-ubiquitylation often leads to protein degradation. However, H2A did not change in MRL/lpr mouse kidneys or in HPC with WWP2 knockout or overexpression (**Figure S3**), suggesting that poly-ubiquitylated H2A is less likely to mediate WWP2's effect in LN.

Ubiquitylation omics analysis demonstrated that the ubiquitylation of H2A at K119 was upregulated in WWP2-overexpressing cells. Notably, up to 10% of H2A harbors K119 mono-ubiquitylation (i.e., ub-H2A) in mammalian cells 23. Ub-H2A is a highly conventional modification in vertebrate animals, governing the maintenance of cellular identity and proper differentiation under the regulation of its E3 ligase, RING1A/B 23. It remains unclear whether other E3 ligases mediate the modification of ub-H2A in various contexts (e.g., other cells and/or pathological conditions).

We used a monoclonal antibody to detect the reaction product of the *in vitro* ubiquitylation assay and found the presence of ub-H2A in this cell-free system (**Figure 5F**). These results confirmed that WWP2 may be a novel E3 ligase producing ub-H2A. Furthermore, ub-H2A and WWP2 colocalized in the glomeruli of MRL/lpr mice (**Figure 5G**). Ub-H2A level was dependent on the abundance of WWP2 in both MRL/lpr kidneys and HPCs (**Figure 5H-M**). These data indicated that ub-H2A may be a novel ubiquitylation product of WWP2, subsequently mediating WWP2's effect on podocyte injury in LN.

### Ub-H2A May Mediate the Effect of WWP2 on Podocyte Injury in LN

**Figure 6:**
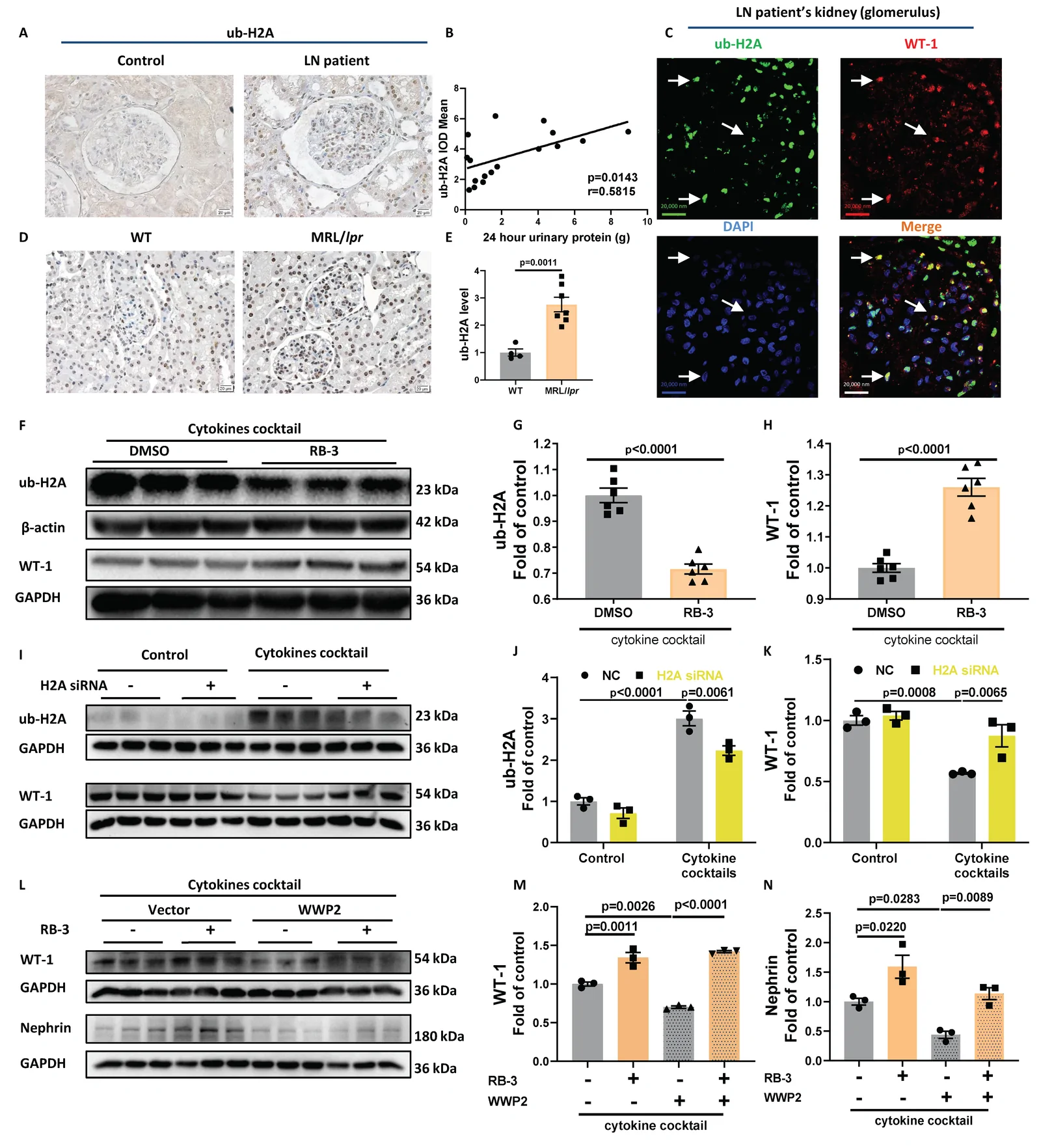
** Ub-H2A may Mediate the Effect of WWP2 on Podocyte Injury in LN.** The clinical correlation of ub-H2A with the severity of LN was studied in our cohort containing 17 LN patients and 4 age- and gender-matched controls. The levels of Ub-H2A in the glomeruli of patients with LN and 18-week-old female MRL/lpr mice and their age- and gender-matched genetic background control MRL/MPJ mice were examined via immunohistochemical analysis. (**A-B**) Glomerular ub-H2A was significantly correlated with patients' proteinuria. **A**: Representative images of immunohistochemical analysis on LN patients with ub-H2A. **B**: ub-H2A protein level was significantly positively correlated with 24-hour proteinuria in LN patients. (**C**) Ub-H2A was colocalized with WT-1, the podocyte marker enriched in the nuclei, in LN patients' glomeruli. Arrow showing the localization of ub-H2A in WT-1-positive DAPI staining. Green: Ub-H2A; Red: WT-1; Blue: DAPI. (**D-E**) ub-H2A was significantly elevated in the glomeruli of MRL/lpr mice. n=4, 7 for MRL/MPJ and MRL/lpr mice, respectively. Scale bar: 20 μm in **A**, **C**, **D**. (**F-H**) A selective inhibitor, RB-3, effectively blocked ub-H2A and significantly increased WT-1. n=6 biological replicates. (**I-K**) H2A siRNA significantly decreased ub-H2A and increased WT-1 in podocyte culture exposed to cytokines cocktail. n=3 biological replicates. (**L-N**) RB-3 blocked WWP2's effect on podocyte injury by restoring WT-1 and nephrin protein levels. n=3 biological replicates. Bar charts are presented as mean ± SEM. P-values for two-groups comparison or multiple-groups comparison were calculated by Two-tailed unpaired Student's *t*-test and one-way ANOVA followed by Sidak's multiple comparisons test, respectively.

Physiologically, ub-H2A regulates embryogenesis and cell identity maintenance 23. Its pathological role remains largely unclear 24. We investigated the potential implication of ub-H2A in LN pathology and found that ub-H2A levels increased in the nuclei of patients' glomeruli (**Figure 6A**) and correlated with the proteinuria level (**Figure 6B**). Ub-H2A was located in the podocyte nuclei, as labeled by WT-1, the podocyte's nuclear marker 28, in patients with LN (**Figure 6C**). Consistently, ub-H2A level was significantly increased in the glomeruli of MRL/lpr mice (**Figure 6D-E**). These findings suggest ub-H2A's potential implication in LN pathology.

RB-3 is a recently-developed, selective inhibitor of ub-H2A 33. RB-3 treatment or H2A knockdown with siRNA (**Figure S4**) effectively decreased ub-H2A and increased WT-1 in cytokine cocktail-stimulated HPC (**Figure 6F-K**). These findings indicate that ub-H2A inhibition may alleviate podocyte injury induced by pro-inflammatory stimulation. Moreover, decreasing ub-H2A levels with RB-3 significantly abolished WWP2's aggravation of podocyte injury, as reflected by the protein levels of WT-1 and nephrin (**Figure 6L-N**). Thus, ub-H2A may mediate the WWP2's effect on podocyte injury induced by pro-inflammatory stimulation.

### Ub-H2A may Mediate the Effect of WWP2 on Podocyte Injury by Enhancing FAK Signaling

**Figure 7:**
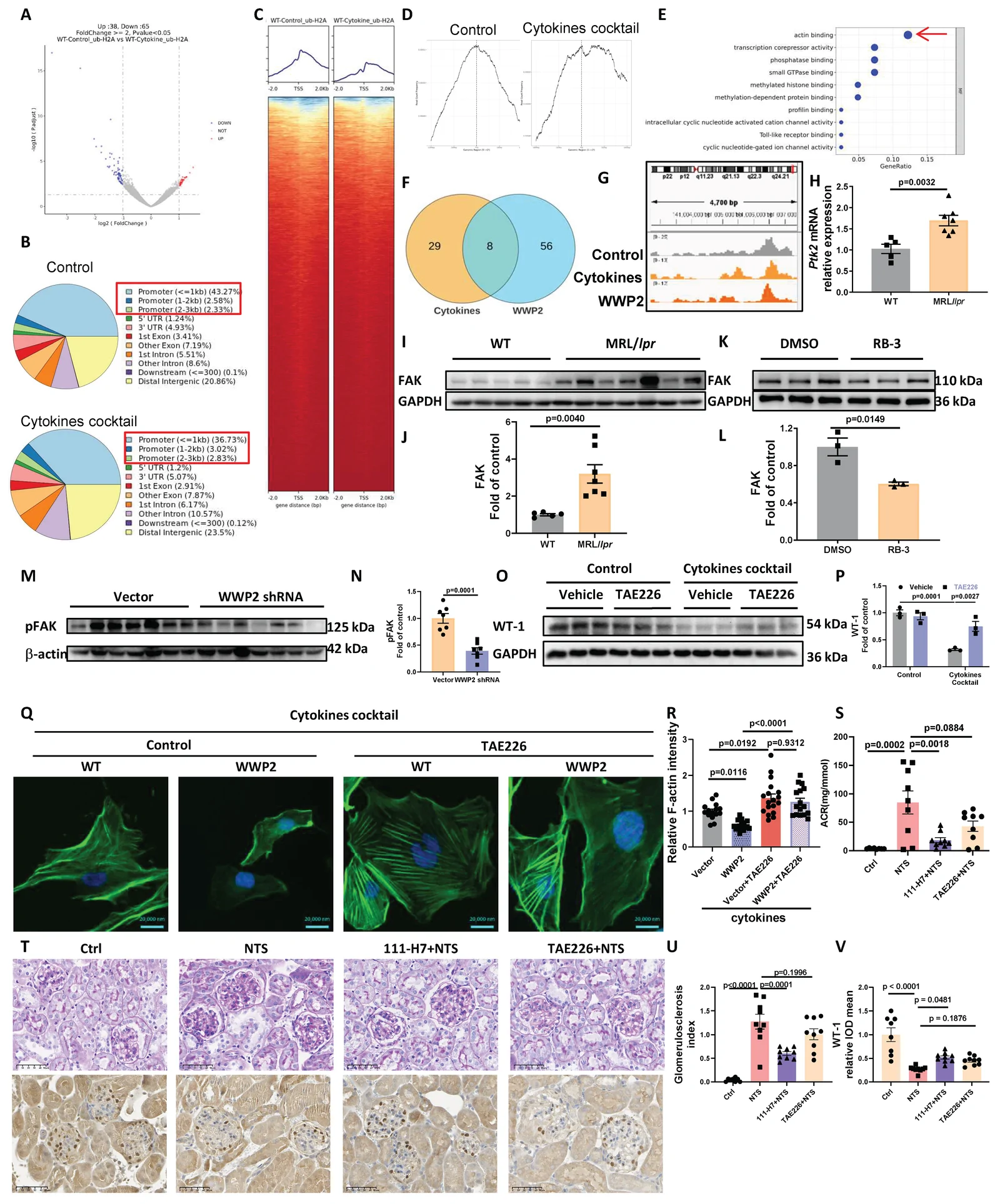
** Ub-H2A may Mediate the Effect of WWP2 on Podocyte Injury by Enhancing FAK Signaling.** CUT&Tag sequencing analysis of ub-H2A was performed on HPC with or without cytokines cocktail stimulation. (**A**) Volcano map showing that 38 and 65 genes were upregulated and downregulated, respectively, with ub-H2A binding in cytokines-stimulated HPCs according to CUT&Tag analysis. (**B-D**) Cytokines exposure decreased the binding of ub-H2A on promoter regions, as shown by the Binding region analysis (**B**) and TSS analysis (**C-D**). **B**: pie chart summarizing the binding region of ub-H2A under cytokines stimulation. **C-D**: TSS analysis showing that ub-H2A binding regions shifted away from promoters under the stimulation of cytokines in HPCs. (**E**) GO analysis on ub-H2A-bound genes at promoter highlighted that cytokines stimulation profoundly affected actin-binding protein, including EVL/WDR1/PTK2/SNTG2/FLNA. (**F**) Venn map on ub-H2A-binding down-regulated genes of cytokines group and WWP2 group showed 8 candidates that WWP2 and ub-H2A may downregulate during cytokines cocktail stimulation, including UHRF1/LY6E-DT/RUNX1/NDRG1/NBPF11/PTK2/C1QTNF1/LOC105375674. (**G**) *PTK2* promotor was downregulated in both cytokine-stimulated and WWP2-overexpressing HPCs. (**H-J**) *Ptk2* mRNA expression and its encoded protein FAK was significantly upregulated in MRL/lpr mice. n=5 and 7 for WT control and MRL/lpr mice. (**K-L**) Suppressing ub-H2A with RB-3 significantly decreased FAK in HPCs. n=3 biological replicates. (**M-N**) WWP2 knockdown significantly decreased p-FAK in MRL/lpr mouse kidneys. n=7. (**O-P**) Inhibiting FAK with TAE226 significantly increased WT-1 in cytokines cocktail-stimulated HPC culture. n=3 biological replicates. (**Q-R**) Blocking FAK activation with TAE226 rescued podocyte injury in WWP2 overexpression HPCs with cytokines cocktail stimulation, as shown by the restoration of F-actin homeostasis. **Q**: representative confocal images of HPCs. Green: F-actin; Blue: DAPI. Scale bar: 20 μm. **R**: Quantification of fluorescent intensity of F-actin (right). (**S-V**) Treatment of H111-H7, a novel WWP2 inhibitor, significantly alleviated proteinuria (**S**), glomerulosclerosis and podocyte injury (**T-V**) and in NTS model. Scale bar: 50 μm. The inhibition against WWP2's ubiquitylation activity is reported on our recent work 20. Bar charts are presented as mean ± SEM. P-values for two group- or multiple group-comparison in** H, J, L, N** and **V** were calculated by Two-tailed unpaired Student's *t*-test or One-way ANOVA followed by Dunnett's (**V**) post hoc test. P-values for the rest were determined by One-way ANOVA followed by Sidak's multiple comparisons test.

Ub-H2A is broadly distributed in the genome, including the promoter, gene body, and intergenic regions 23. Ub-H2A reinforces nucleosome's mechanical stability by impeding DNA detachment; hence, ub-H2A may suppress transcription by binding with or near the promoter, whereas it may activate transcription if such binding decreases 24. Cleavage under targets and tagmentation (CUT&Tag) sequencing showed more genes exhibited decreased binding with ub-H2A (65 genes) compared to those increased (38 genes) in HPC with cytokines stimulation (**Figure 7A**). Binding region analysis demonstrated that the cytokine cocktail shifted ub-H2A away from the promoters (48.18% vs 42.58%; **Figure 7B-D**). This binding mode remodeling, despite overall increased ub-H2A levels, likely alleviates ub-H2A-mediated transcriptional repression, leading to the enhanced expression of affected genes in cytokine-stimulated HPCs.

GO analysis of the ub-H2A-bound genes at the promoter suggested that ub-H2A may primarily govern the gene expression of actin-binding proteins, including focal adherin kinase (FAK, encoded by *PTK2*), in cytokine cocktail-stimulated HPC cells (**Figure 7E**). Further Venn mapping of downregulated genes in cytokine-stimulated and WWP2-overexpressing HPCs in CUT&tag analysis of ub-H2A pinpointed *PTK2* (**Figure 7F-G**). *PTK2* mRNA expression and protein level were significantly increased in MRL/lpr mouse kidneys (**Figure 7H-J**). FAK protein levels were suppressed by RB-3, the ub-H2A inhibitor, in HPC (**Figure 7K-L**). WWP2 knockdown decreased p-FAK, an indicator of FAK activation, in the kidneys of MRL/lpr mice (**Figure 7M-N**), further suggesting that FAK may be a downstream mediator of WWP2/ub-H2A signaling on podocyte injury in LN.

FAK is an essential kinase regulating focal adhesion formation 34. In lipopolysaccharide (LPS)-stimulated podocytes, FAK activation leads to an alteration in the actin structure, as shown by the disturbance in the F-actin structure 34. Blocking FAK activation with TAE226, a FAK inhibitor, protected against proteinuria in the LPS model by preserving the F-actin structure in podocytes 34. We also found that TAE226 restored WT-1 protein levels and preserved the F-actin structure in cytokine-stimulated HPCs (**Figure 7O-R**). TAE226 treatment antagonized WWP2-mediated exacerbation in podocyte injury (**Figure 7Q-R**), indicating that FAK activation may be a mediator through which WWP2 affected podocyte injury.

To further demonstrate the therapeutic potential against proteinuria in nephritis by antagonizing WWP2, we treated the NTS-induced mouse nephritis model with H111-H7 (CAS No. 220965-54-8), a novel WWP2 inhibitor recently developed by our group 20. H111-H7 treatment significantly decreased proteinuria, alleviated glomerulosclerosis and podocyte injury in the NTS model, similar to TAE226, whose therapeutic potential in NTS model has been previously reported 34 (**Figure 7S-V**). These data demonstrated a therapeutic potential of WWP2 inhibitor against nephritis and further suggest a potential implication of FAK-mediated actin dynamics in the pathological effect of WWP2 in LN.

## Discussion

LN, a clinically-common comorbidity of SLE 35, symptomatically manifests a progressive increase in proteinuria, which is pathologically attributed to podocyte injury 5-7. Currently, the clinical management of LN is limited to immune-suppressants, whose renal protection is often unsatisfied 5. Target treatment alleviating proteinuria and renal pathology is in urgent demand 4, 35. An improved understanding of the mechanisms driving podocyte injury may lead to novel therapeutic strategies for LN. In the present study, we revealed that the elevated WWP2 aggravated podocyte injury and proteinuria in LN through its novel ubiquitylation product ub-H2A. Our work demonstrates a novel pathological role of WWP2 in LN-associated podocyte injury, and proposes WWP2 inhibition as a novel therapeutic strategy against LN.

WWP2, an E3 ligase, regulates cell differentiation, cell proliferation, and apoptosis, and participates in the pathology of carcinogenesis, fibrosis, hypertension, and physiological differentiation of stem cells, T cells, neurons, and chondrocytes 11, 15-17, 36. In the kidneys, WWP2 may physiologically regulate sodium channel homeostasis in the distal tubules 37. In our serial studies on WWP2's effect on renal tubulointerstitial pathology, we demonstrated the distribution of upregulated WWP2 in kidney cells, including tubular epithelial cells in AKI and AKI-to-CKD kidneys, and fibroblasts in fibrotic kidneys 18, 19. The pathological progression of kidney diseases is a synergistic combination of various cellular events which dynamically features each progression stage. Glomeruli are primarily affected in LN manifested with IC deposition and inflammation, and symptomatically featured with progressive proteinuria. Inflammatory podocyte injury may largely contribute to proteinuria 5-7. Tubular injury lies in the pathological foundation of AKI, and tubulointerstitial injury may be secondary to glomerular lesions and proteinuria 2. It is possible that the distribution of the upregulated WWP2 was associated with the pathological conditions. Our present work demonstrated that WWP2 was significantly upregulated in the glomeruli of patients with LN and was positively correlated with proteinuria. WWP2 localized in the podocytes, whose nuclei were marked by WT-1, in LN kidneys. We also noticed that WWP2 was not exclusively expressed in the podocytes, but broadly distributed in the cells inside and outside of the glomeruli. This pattern was consistent with our previous finding that demonstrating WWP2's distribution in the tubulointerstitial region in kidneys with acute or chronic lesions 18-20. Since podocyte injury plays pivotal role in proteinuria in LN, we examined the podocytes exposed to pro-inflammatory stimuli, such as the serum of patients with LN or cytokine cocktail, and found an upregulation of WWP2 in the stimulated podocytes. These results indicated that WWP2 upregulation in podocytes may be implicated in the pathological progression of LN.

CKD is a chronic pathological condition that affects nearly all sorts of renal residential cells. Depending on their property and metabolic features, each type of cells shows distinct pathological changes. We uncovered WWP2's context-dependent roles in kidney diseases in our previous studies. Deletion of WWP2 in tubular epithelial cells further aggravated acute kidney injury. After the acute stage, WWP2 aggravates the various pathological courses in CKD. In the tubulointerstitium, the sustained elevated level of WWP2 promotes the AKI-to-CKD transition via partial EMT in tubular epithelial cells and exacerbates ECM production via regulating differential orientations of fibroblasts subtypes 18-20. In diabetes-associated vascular endothelial injury, WWP2 was downregulated and vascular endothelial cell-specific WWP2 deletion may further aggravate vascular endothelial injury and vascular remodeling in type 2 diabetic mellitus 38. Podocytes exhibit unique structural and functional characteristics: they are terminally differentiated cells with limited regenerative capacity and are continuously exposed to circulating inflammatory mediators 5, 6. Podocyte injury caused by inflammation is a central event in the progression of proteinuria in LN 5-7. Considering the unique pathogenic mechanisms underlying podocyte damage and their differential responses to injury compared with other renal cell types, we investigated WWP2's implication in podocyte injury in LN. Our present study showed the implication of WWP2 in podocyte injury in CKD caused by immune disorder, which may further complete the pathological roles played by WWP2.

To study the effect of WWP2 on LN progression, we employed both spontaneous and induced models mimicking IC-induced glomerulonephritis in LN, i.e., the MRL/lpr mice and NTS glomerulonephritis model, respectively 39. MRL/lpr mice are relevant to SLE because the autoantibodies and IC are produced by autoreactive immune cells 39. We manipulated WWP2 protein levels after proteinuria onset in female MRL/lpr mice 29, 40. Intra-renal injection of AAV2/9 is effective for gene delivery in CKD kidneys, including podocytes 30. Our data confirmed a robust exogenous mNeonGreen expression and efficient, specific modulation of WWP2 protein levels (both overexpression and knockdown) in MRL/lpr kidneys using the AAV2/9 system. WWP2 knockdown significantly inhibited proteinuria progression, alleviated glomerular lesions, and protected against podocyte injury by attenuating foot process effacement and preserving podocyte-specific markers. WWP2 overexpression significantly exacerbated proteinuria and podocyte injury in MRL/lpr mice. We also observed that tubulointerstitial injury was altered in parallel with glomerular pathological changes in MRL/lpr mice kidneys upon WWP2 manipulation. These results further supported the pathological role of WWP2 in glomerulonephritis in LN. Intravenous injection of AAV2/9 may be inefficient for transduction in bone marrow-derived cells and leukocytes infiltrated into the kidneys 41; therefore, the observed effect of WWP2 manipulation in MRL/lpr mouse kidneys was likely exerted on the kidney cells.

Podocyte injury underlies the proteinuria progression in LN and may indicate a poorer prognosis for patients with LN 5, 42. We generated a podocyte specific WWP2 deletion mouse strain to decipher the effect of WWP2 signaling in the podocyte on glomerulonephritis caused by immune disorder seen in LN. Since the cKO mice have the non-autoimmune C57BL/6J background, which would prevent the MRL/lpr mice to manifest symptoms 39, we employed the induced glomerulonephritis model by NTS. NTS are crude prepared serum from heterologous donor with mostly anti-GBM and also other kidney components, manifesting LN-like IC-deposition glomerulonephritis caused by multiple antibodies such as anti-GBM and anti-donor antibodies 31, 39. Despite that NTS model and MRL/lpr mice have different origin of IC, their glomerulonephritis are induced by IC-deposition and inflammatory response and both models are utilized to study LN 39. NTS model may not be prominent in crescent formation 43. We found that WWP2 cKO in podocyte effectively alleviated proteinuria, glomerulosclerosis and podocyte injury in the NTS glomerulonephritis model 31, 32. The results from spontaneous and induced LN models and kidney or podocyte targeting WWP2 silencing consistently demonstrated that inhibiting WWP2 in the kidneys, including podocyte, attenuated glomerulonephritis in LN. WWP2 deficiency directly prevented podocyte injury in an *in vitro* model for podocyte injury in LN 7, whereas WWP2 overexpression aggravated podocyte injury. Our data from LN clinical samples and experimental models demonstrated that WWP2 may boost podocyte injury in LN.

To investigate the underlying mechanism, we reanalyzed the ubiquitylation omics dataset of WWP2-overexpressing mouse tubular epithelial cells 18. Enrichment analysis indicated that WWP2 may affect the SLE pathway via ubiquitylating H2A. Further co-IP and *in vitro* ubiquitylation analyses demonstrated that H2A may be a novel ubiquitylation substrate of WWP2, yielding ub-H2A to mediate WWP2's effect on LN podocyte injury. Ub-H2A is a conserved and abundant H2A post-translational modification in vertebrates 23. Physiologically, ub-H2A governs cell identity maintenance and differentiation during embryonic development under the regulation of E3 ligase RING1A/B 11, 24, 33. However, its pathological role and regulatory mechanisms remain largely unclear. We detected ub-H2A in the yield of the WWP2-H2A *in vitro* ubiquitylation assay and found a colocalization of ub-H2A and WWP2 in the glomerulus of the LN model. Ub-H2A changed alongside WWP2 in MRL/lpr kidneys and HPC. These data indicated that ub-H2A may be WWP2's ubiquitylation product and subsequently mediates the effect of WWP2 on podocyte injury in LN. The increased Ub-H2A in the glomeruli was significantly correlated with proteinuria in patients with LN. Decreasing ub-H2A with H2A knockdown or RB-3, a selective inhibitor of RING1B 33, prevented podocyte injury in the inflammatory model and inhibited WWP2-related exacerbation of podocyte injury. These findings indicate that ub-H2A may functionally mediate WWP2's effect in exacerbating podocyte injury in LN as its ubiquitylation product.

Ub-H2A impedes DNA detachment from the nucleosome as a “bolt.” Depending on the binding quantity and gene regions, ub-H2A can bidirectionally regulate gene expression—suppressing it when accumulating on the promoter, and facilitating its activation when detached 11, 24, 33. Our CUT&Tag sequencing data revealed reduced ub-H2A binding, particularly at gene promoters, in cytokine-exposed HPCs, suggesting a potential activation of the affected genes. GO analysis highlighted a primary regulation by ub-H2A in the actin cytoskeletal stability pathway in cytokine cocktail-stimulated podocytes. The integrity and dynamics of the actin cytoskeleton are essential for podocytes to maintain the foot process, anchoring podocytes to the basement membrane and forming a slit diaphragm with neighboring podocytes. Disruption of podocyte actin dynamics underlies the pathogenic onset of proteinuria and nephrotic syndrome 5. Further Venn mapping and validation indicated that FAK may lie downstream of WWP2/ub-H2A in LN.

FAK is an essential kinase regulating focal adhesion formation and is activated in podocytes under pro-inflammatory stimulation 34. Blocking FAK by podocyte-specific FAK silencing or TAE226 significantly alleviated proteinuria in the NTS model 34. We found that FAK inhibition attenuated cytokine-induced podocyte injury, and abolished the aggravation of injury by WWP2. Furthermore, our novel WWP2 inhibitor 20 showed a similar therapeutic effect against proteinuria in the NTS model as TAE226. This finding supported that FAK lies downstream of WWP2 in LN and indicated the therapeutic potential of WWP2 inhibitor.

Our study has the following limitation. Firstly, we did not perform podocyte specific WWP2 manipulation in the MRL/lpr mice. Our data showed that knockdown WWP2 in the kidney significantly alleviated proteinuria and podocyte injury in the MRL/lpr mice, suggesting targeting WWP2 may be a novel therapeutic strategy against LN. Also, podocyte specific deletion of WWP2 alleviated NTS induced nephritis, indicating that targeting on WWP2 in the podocyte may dampen the pathological progression of nephritis caused by immune disorder. Secondly, LN affects the entire kidneys which primarily manifest glomeruli lesion and secondarily tubulointerstitial injury. WWP2 distributed in cells inside and outside of glomeruli. Currently, there is limited study that defines WWP2's effect on pathological changes in cells such as endothelial cells and mesangial cells in LN. Existing study showed that WWP2 was downregulated in diabetes-associated vascular endothelial injury, and WWP2 deletion aggravated diabetic associated vascular endothelial injury 38. However, since LN has unique etiology and pathology, it remains unclear whether WWP2's regulatory effect in LN-associated pathology was similar or distinct to the finding in diabetic condition. Further investigation using cell type-specific knockout mice and cell culture experiments may precisely delineate WWP2's implication in LN-associated pathology in different cell types and a more comprehensive mechanism underlying the therapeutic efficacy of WWP2 inhibition.

In conclusion, our study identified WWP2/ub-H2A/FAK signaling axis as a key promoter of podocyte injury in LN. Antagonizing this pathway may be a promising novel therapy for LN.

## Supplementary Material

Supplementary materials and methods, figures.

## Figures and Tables

**Table 1:** Clinical diagnosis and renal pathological parameters of patients with LN

Patient No.	Gender	Age	Clinical Diagnosis	Pathological diagnosis	24h proteinuria (g)
1	Female	9y	LN, SLE, URTI	LN III(A)	0.20625
2	Female	14y9m	SLE, LN	LN II	0.272
3	Female	11y8m	LN? SLE?	LN IV-G(A)	1.06175
4	Female	7y11m	SLE , LN	LN IV-G(A)	0.49
5	Female	11y9m	Purpura nephritis	LN IV-G(A)+V	1.752
6	Female	12y11m	LN	LN IV-G(A/C)	6.456
7	Female	13y7m	SLE, LN	LN II	0.968
8	Female	12y11m	SLE, LN	LN IV-G(A)	0.5454
9	Female	10y5m	SLE, LN, acute URTI	LN IV-G(A/C)	4.03225
10	Female	14y10m	SLE	LN IV-G(A)	1.4238
11	Female	4y1m	LN SLE	LN IV-G(A)	4.30939
12	Female	7y0m	LN, SLE (severe case), acute URTI	LN IV-G(A)	4.802
13	Female	9y11m	SLE	LN II	0.09
14	Female	6y10m	LN	LN IV(G)+V	0.145
15	Female	13y0	SLE, LN, bronchitis	LN III + V	1.65375
16	Female	7y10m	SLE, LN, bipneumonia	LN IV+V	5.058
17	Female	14y5m	LN, SLE	LN V+III	8.94

## Data Availability

Ubiquitylation omics data is available to the public (ProteomeXchange Dataset, PXD048540). CUT&Tag sequencing data supporting this study is available for the reviewers in GEO308439 dataset (https://www.ncbi.nlm.nih.gov/geo/query/acc.cgi?acc=GSE308439) with the token wrojyowijvcbjix. Original images of western blotting for all the replicates were provided as supplemental material.

## Abbreviations

AAV: adeno-associated virus
ACR: albumin/creatinine ratio
CUT&Tag: cleavage under targets and tagmentation
CKD: chronic kidney disease
FAK: focal adhesion kinase
GBM: glomerular basement membrane
GO: Gene Ontology
HPC: human podocyte culture
IC: immune complex
IP: immunoprecipitation
KEGG: Kyoto Encyclopedia of Genes and Genomes
LDH: lactate dehydrogenase
LN: lupus nephritis
NTS: nephrotoxic serum
PAS: periodic acid-Schiff
SLE: Systemic lupus erythematosus
TEM: transmission electron microscopy
ub-H2A: Monoubiquitylation of H2A at the K119 site
WT-1: Wilms' Tumor-1
WWP2: WW domain containing E3 ubiquitin protein ligase 2
WWP2 cKO: Podocyte-specific WWP2 knockout
