# Supplementary material for: Enhanced WWP2 Exacerbates Podocyte Injury in Lupus Nephritis through Monoubiquitylating H2A at K119
Source: Int J Biol Sci. 2026 Jul 22;22(13):6969–84. doi: 10.7150/ijbs.131475 (PMC13540443; doi:10.7150/ijbs.131475)

Supplemental Materials

Materials and Methods

Materials

WWP2 adeno-associated virus (AAV): the AAV2/9 carrying mouse *Wwp2* gene transcript (NM 025830.4) on the pAAV-CMV-Wwp2-3xFLAG-P2A-mNeonGreen-tWPA vector. The AAVs were provided by Obio, Shanghai, China.

WWP2 shRNA AAV: The shRNA targeting the mouse *Wwp2* gene transcript (XM 006531299.4) with a sequence of CCAATGTCCTGAAGAACAA cloned on pAV-U6-shRNA-CMV-GFP in AAV2/9. The AAVs were provided by WZ Biosciences, Shandong, China.

WWP2 lentivirus was the NM 001270454.2 transcript of the human WWP2 gene expressed on pSLenti-SFH-EGFP-P2A-Puro-CMV-WWP2-3xFLAG-WPRE provided by Obio, Shanghai, China.

WWP2 CRISPR/Cas 9 sgRNA targeting Exons 13 and 14 of human *WWP2* gene had sequences of TAACACTCGCACGACCCAGT GGG and TCCCATCCTGGGGGCAGAGC TGG, resulting in a 644 bp ablation in the *WWP2* gene. The sgRNA was constructed as pSpCas9(BB)-2A-Puro (PX459)-sgWWP2-1# and provided by GeneBay, Jiangsu, China.

Three siRNAs targeting human *H2AC20* were synthesized by Ribio, Guangdong,

China (stB0008523). The target sequence of the siRNAs were GCCGTTCTGTTACCAAAGA; GAAAGCCACAAAGCCAAAA; AGGCCGTTCTGTTACCAAA.

Patient Information

Seventeen pediatric patients with LN and 4 patients with para-carcinoma renal tissues but without chronic kidney diseases were enrolled at the Children's Hospital of Nanjing Medical University. The patients' Clinical parameters are shown in **Table 1**. These patients with LN were all female despite that we did not take sex as a variation, possibly due to the fact that SLE was female-prone. WWP2/ub-H2A abundance in the glomeruli was quantified using the immunofluorescent or immunohistochemical images, respectively, and correlated with patients' 24-hour proteinuria. The study protocol was approved by the Ethics Committee of Children's Hospital of Nanjing Medical University (Approval No. 202304069-1).

Animal study

The protocol for the animal study was approved by IACUC in Nanjing Medical University (Approval No. 2007001-3). The mice were randomly grouped based on their body weights, except when otherwise indicated for MRL/lpr mice. The handling and treatment of the mice were performed by an experienced operator who was blinded to the grouping. The study was conducted in accordance with the IACUC guidelines and under their surveillance.

Wwp2 genetic knockout mice strains

Podocyte-specific WWP2 knockout (WWP2 cKO) mice were bred using the loxp/Cre system by crossbreeding *Wwp2$^{flox/flox}$* mice (strain no. T005371, GemPharmatech, Jiangsu, China) and *Nphs2*-Cre mice (Stock No: 008205, Jackson Laboratory, California, USA), which had genetic background of C57BL/6J. *Wwp2$^{flox/flox}$* littermates served as controls. The breeding strategy was as previously reported [1]. The knockout efficiency of Wwp2 cKO mice was determined using western blotting of primary culture of podocytes, whose extract and culture procedure followed the reported protocol [2, 3]. The mice were bred, housed, treated, and operated on in the corresponding rooms in specific pathogen-free animal unit of Nanjing Medical University.

NTS model and administration

The establishment of the NTS model was similar as previously described [4, 5]. Briefly, 8-week-old male WWP2 cKO mice and their controls (*Wwp2$^{flox/flox}$* mice) received single injections of nephrotoxic serum (NTS, PTX-001AGBM, Probetex, USA), i.e., sheep anti-rat isolated glomerular basement membrane (GBM) serum, or control (sheep non-immune serum, PTX-000S, Probetex, Texas, USA) at the dosage of 18 μl/g body weight via intraperitoneal injection [4]. WWP2 inhibitor (H111-H7, CAS No. 220965-54-8, PMCID: 2819994, purchased from Maybridge, Thermo Fisher, USA) was dissolved in 5% dimethylsulfoxide saline and administrated by intraperitoneal injection to mice at a dosage of 1 mg/kg/d. TAE-226 (HY-13203,

Shanghai, China) was suspended in 0.5% methylcellulose and administrated to mice at 25 mg/kg/d via oral gavage, as previously described [6]. The vehicle control groups received both vehicles. The mice were pre-treated 7 days before NTS injection and received daily administration until the experimental endpoint in accordance with prior study [6]. The mice were euthanized for tissue harvesting after instant urine collection 4 weeks after serum injection.

Surgery and experimental procedure

MRL/lpr mice (Strain No. 000485) were purchased from Jackson Laboratory and bred in the Specific Pathogen Free animal unit at Nanjing Medical University. MRL/MPJ mice of the same sex and age served as genetic background control (WT). SLE is a female-prone disease and female MRL/lpr mice have accelerated progression and more severe symptoms than the males [7]; hence, we used the females in the experiment. Female MRL/lpr mice were semi-randomly grouped based on their albumin/creatinine ratio (ACR) in the instant urine before intra-renal AAV injection. To manipulate WWP2 gene expression in the kidney, we generated WWP2 overexpression or WWP2 shRNA AAV2/9 and performed intrarenal injection of 1 X 10^{12} AAV in 20 μl sterile saline per kidney (2×10^{12} per mouse) under anesthesia with isoflurane. Previous literature has reported the efficacy of intra renal perfusion of AAV2/9 in gene delivery in the kidney [8]. Instant urine was collected for ACR testing. Mice serum and kidneys were sampled at the experimental endpoint for further analysis.

Albumin and creatinine quantification

Albumin and creatinine concentrations in the instant urine were measured using the Mouse albumin ELISA kit (E99-134, Bethyl, Texas, USA) and creatinine quantification kit (C011-2-1, Jiangcheng, Nanjing, China), respectively.

Tissue processing and staining

The kidney samples were dissected and post-fixed in 4% paraformaldehyde for 72 h. Thereafter, the samples were dehydrated and parafilm-embedded before sectioning into 4 μm. Afterward, the tissue sections were rehydrated for further examination such as periodic acid-Schiff (PAS) staining, as previously reported [9]. The entire tissue of PAS staining sections were scanned and cropped for five non-overlapping fields with same scale and magnification (KF-PRO-020, KFBIO, Zhejiang, China) for the samples in **Figure 7T**. For the PAS staining in other experiments, tissue sections were imaged with a light microscope (Olympus, Japan) for five non-overlapping fields. Glomerular sclerosis scoring were performed by a blinded and skillful experimenter following the criteria in literature [10]. Briefly, we first score the sclerosis severity of each glomerulus based on the area of the sclerosis occupying glomerular tuft: grade 0: 0%, grade 1: 0-25%, grade 2: 25-50%, grade 3: 50-75%, grade 4: >75%. Then we calculate the glomerulosclerosis index with the equation: (1 × number of grade 1 glomeruli) + (2 × number of grade 2 glomeruli) + (3 × number of grade 3 glomeruli) + (4 × number of grade 4 glomeruli)/total number of glomeruli examined for each individual. Crescent proportion was quantified as

described in literature [11, 12]. Briefly, crescent is more than 2 cell layers with possible deposition of fibrin and fibrous matrix. A crescent glomerulus is defined if crescent involves 10% or more of the glomerular capsular circumference [11]. Tubular injury score was defined as the proportion of tubular injury area: 0: normal; 1: <10%; 2: 10-15%; 3: 25-50%; 4: 50-75%; 5: 75-100% [13]. The definition of tubular injury was described in [13].

Immunofluorescent Staining and Immunohistochemical Analysis

The protocol for immunofluorescent staining and immunohistochemical analysis was as previously described [9]. Briefly, in immunofluorescent staining, the parafilm sections were de-parafilm and rehydrated before being blocked with 5% normal goat serum and incubated overnight with 1:100 WWP2 antibody (12197-1-AP, Proteintech, Hubei, China), before secondary antibody recognition and counterstaining with DAPI [14]. The specificity of the WWP2 antibody has been demonstrated in knockout mouse kidneys in our previous study [14]. WWP2 Co-staining with 1:200 WT-1 antibody (ab267377, Abcam, Shanghai, China) or ub-H2A (8240, CST, Massachusetts, USA) was performed using TSA multiple staining kit (RK50026P, abclonal, Hubei, China) according to the manufacturer's protocol. The images of immunofluorescent staining were acquired using confocal microscopy (LSM700, Carl Zeiss, Germany) to image all the glomeruli in the biopsy samples of the patient and quantified for fluorescent intensity using Image J software (https://imagej.nih.gov/ij/).

In immunohistochemical analysis, the parafilm sections were deparafilmed,

rehydrated, and stained with 1:200 WT-1 antibody (ab267377, Abcam, Shanghai, China) or ub-H2A (8240, CST, Massachusetts, USA) using PV9000 kits (Zsbio, Beijing, China). The entire tissue of WT-1 immunohistochemical staining sections were scanned and cropped for five non-overlapping fields with same scale and magnification (KF-PRO-020, KFBIO, Zhejiang, China) for each sample in **Figure 7T**. The images of immunohistochemical analysis in other experiments were acquired using light microscopy (Olympus, Japan) for at least five non-overlapping fields. Optical density was quantified using ImageProPlus. The acquisition and quantification of the images was performed by an experienced and blinded experimenter as previously described [9].

Transmission Electron Microscopy

The kidney samples were dissected and fixed for transmission electron microscopy (TEM) examination to assess podocyte integrity. The procedure of TEM examination was similar to previous report [9]. Foot process width was determined by a blinded and experienced operator using Image J software (https://imagej.nih.gov/ij/), as reported in [15, 16].

Cell Culture and Treatment

Culture and treatment

The immortalized human podocyte culture (HPC) was generously provided by Prof. John Cijiang He (Icahn School of Medicine at Mount Sinai, New York, USA)

and cultured as described previously [17]. Briefly, the HPCs were maintained in 33 °C before being transferred to a 37 °C incubator for differentiation. On the 13th day of differentiation, HPC was exposed to a cytokine cocktail comprising human tumor necrosis factor-α (TNF-α, 210-TA, R&D Systems, Minneapolis, USA), interleukin-1β (IL-1β, 96-200-01B-2, Peprotech, Shanghai, China), interferon-α (IFN-α, 11105-1, R&D Systems, Minneapolis, USA), and IFN-γ (96-300-02-20, Peprotech, Shanghai, China) for 24 hours to mimic the pro-inflammatory response in LN *in vitro* [18]. The working concentration of each cytokine was 10 ng/ml [18]. Before the 24-h cytokine cocktail stimulation, the HPC were pretreated with 25 μM RB-3 (HY-139514, MCE, Shanghai, China) for 3 days or 5 μM TAE226 (HY-13203, MCE, Shanghai, China) for 2 h to inhibit ub-H2A [19] and focal adhesion kinase (FAK) activation [6], respectively, as previously reported.

Generation of WWP2 overexpression in HPC cell line

Undifferentiated HPCs were infected with WWP2 lentivirus, which carries the anti-puromycin gene. Seventy-two hours after infection, the uninfected cells were cleared with puromycin (60210ES60, Yeasen, Shanghai, China) in a complete medium. Afterward, the cells were tested for overexpression efficiency.

Generation of WWP2 knockout in HPC cell line

Undifferentiated HPCs were transfected with WWP2 sgRNA, which carries the anti-puromycin gene, using Polyjet transfection reagent (SL100688, SignaGen Laboratories, Rockville, USA). Twenty-four hours later, the non-transfected cells

were cleared with puromycin. Thereafter, monoclonal HPCs were selected and expanded for knockout efficiency validation. The validated knockout clone was used for further investigation.

siRNA Transfection of HPCs

HPCs were differentiated for 12 days before transfected with H2A siRNA by RNAiMax (13778150, Thermofisher). The transfection procedure followed the manufacturer's protocol.

Isolation of histone fraction from HPC and mice kidneys

The histone from HPC or mice kidneys were extracted with Histone isolation kit provided by Proteintech (PK10022, Shanghai, China) with the supplement of protease inhibitor and phosphotase inhibitor. The procedure followed the manufacturer's protocol.

lactate dehydrogenase assay

The supernatant of the HPC cell culture medium was acquired via centrifugation at 1500 × g for 5 min and analyzed for lactate dehydrogenase (LDH) using a biochemical analyzer, as previously described [9]. The value was normalized with the protein mass of the HPC cell homogenate.

Co-immunoprecipitation assay

HEK293T cells expressed with WWP2-Flag for 24 h before harvesting for protein samples with ice-cold immunoprecipitation (IP) lysis buffer (P0013, Beyotime,

Shanghai, China). Equal amount of protein samples was pull-down with Flag (70569, CST, Massachusetts, USA) or IgG-coated beads (rabbit IgG antibody: B900610, Proteintech, Hubei, China; mouse IgG antibody: ab190475, Abcam, Massachusetts, USA) with a co-IP kit (26149, Pierce, Rockford, USA) following the manufacturer's protocol. The input, IP, and IgG samples were examined by western blotting analysis.

Ubiquitylation omics analysis

The ubiquitylation omics data from WWP2 overexpression in tubular epithelial cells (PXD048540, [9]) were re-analyzed by performing Kyoto Encyclopedia of Genes and Genomes (KEGG) analysis of upregulated proteins in ubiquitylation.

***In vitro* ubiquitylation assay**

In vitro ubiquitylation assay was performed in a cell-free system, as previously described but with minor modification [20]. Fifty milliliters of the reaction buffer comprised 40 mM Tris-Hcl 1.6 ml, 2 mM DTT 0.1 ml, 2 mM $MgCl_2$ 0.1 ml, MQ water 47.575 ml, and 2.5 mM ATP 0.625 ml. Each reaction required 48.885 μl reaction buffer to dissolve the recombinant proteins of His6-E1 (E-304-050, R&D systems, Minneapolis, USA), GST-E2 (E2-620-100, R&D systems, Minneapolis, USA), WWP2-GST (P104935, Gene Universal, Anhui, China)—0.2 μg each—together with ubiquitin (Ub, U5382, Sigma-Aldrich, Missouri, US) and substrate, recombinant proteins of H2A-His6 (P102262-1, Gene Universal, Anhui, China) or PTEN (ab157087, Abcam, Massachusetts, USA), 1 μg each. The negative control was the same as the reaction between WWP2 and H2A but without ATP.

WWP2 and PTEN reaction served as a positive control, as previously reported [20]. The reactions were thoroughly mixed and incubated for 30 min at 37 °C. Thereafter, the reaction yields were mixed with loading buffer and denatured at 100 °C for 5 min to ensure examination of the reaction using western blotting analysis.

Western blotting analysis

Western blotting analysis were performed as previously described [9]. The primary antibodies were 1:1000 of the following: WWP2 (sc-398090, Santa Cruz, Texas, USA), mNeonGreen (29523-1-AP, Proteintech, Hubei, China), podocin (ab181143, Abcam, Massachusetts, USA), Wilms' Tumor-1 (WT-1, ab267377, Abcam, Massachusetts, USA), H2A (2578, CST, Massachusetts, USA), ub-H2A (8240, CST, Massachusetts, USA), H3 (17168-1-AP, Proteintech, Hubei, China), cleaved caspase-3 (9664, CST, Massachusetts, USA), Ub (3936S, Abcam, Massachusetts, USA), FAK (12636-1-AP, Proteintech, Hubei, China), p-FAK Tyr 397 (8556, CST, Massachusetts, US), GAPDH (60004-1-Ig, Proteintech, Hubei, China), and β-actin (66009-1-Ig, Proteintech, Hubei, China).

The products of the in vitro ubiquitylation assay were detected with western blotting. After electropherosis in SDS-PAGE gel and transferring to PVDF membrane, the 5% milk-blocked membrane were incubated with primary antibody of ub-H2A or PTEN for their indicated proteins, respectively (8240 and 9188S, respectively, both from CST, Massachusetts, USA), Flag (F1804, Sigma, Missouri, US) for ubiquitylated yields, 6x His tag (66005-1-Ig, Proteintech, Hubei, China) for the

simultaneous detection of H2A and E1 (predicted MW 15 and 121 kDa, respectively), GST tag (2622, CST, Massachusetts, USA) for the simultaneous detection of E2 (43 kDa) and WWP2 (130 kDa). Alternatively, we also detected the reactions with anti-H2A (2578, CST, Massachusetts, USA) and anti-WWP2 (sc-398090, Santa Cruz, Texas, USA) as confirmation.

Cleavage under targets and tagmentation sequencing analysis

Cleavage under targets and tagmentation (CUT&Tag) assay was performed using the commercially available kit (TD903, Vazyme, Jiangsu, China) following the manufacturer's protocol. Briefly, the cells were harvested and bound with ub-H2A primary antibody (8240, CST, Massachusetts, USA) overnight at 4 °C. Thereafter, the samples were recognized with secondary antibody followed by hyperactive pA/G-transposon interaction. Afterward, the samples were fractionated and extracted for DNA. The DNA samples were amplified for sequencing on Illumina NovaSeq 6000 (California, USA).

Supplemental Results

WWP2 Exacerbated Proteinuria and Podocyte Injury in MRL/lpr Mice

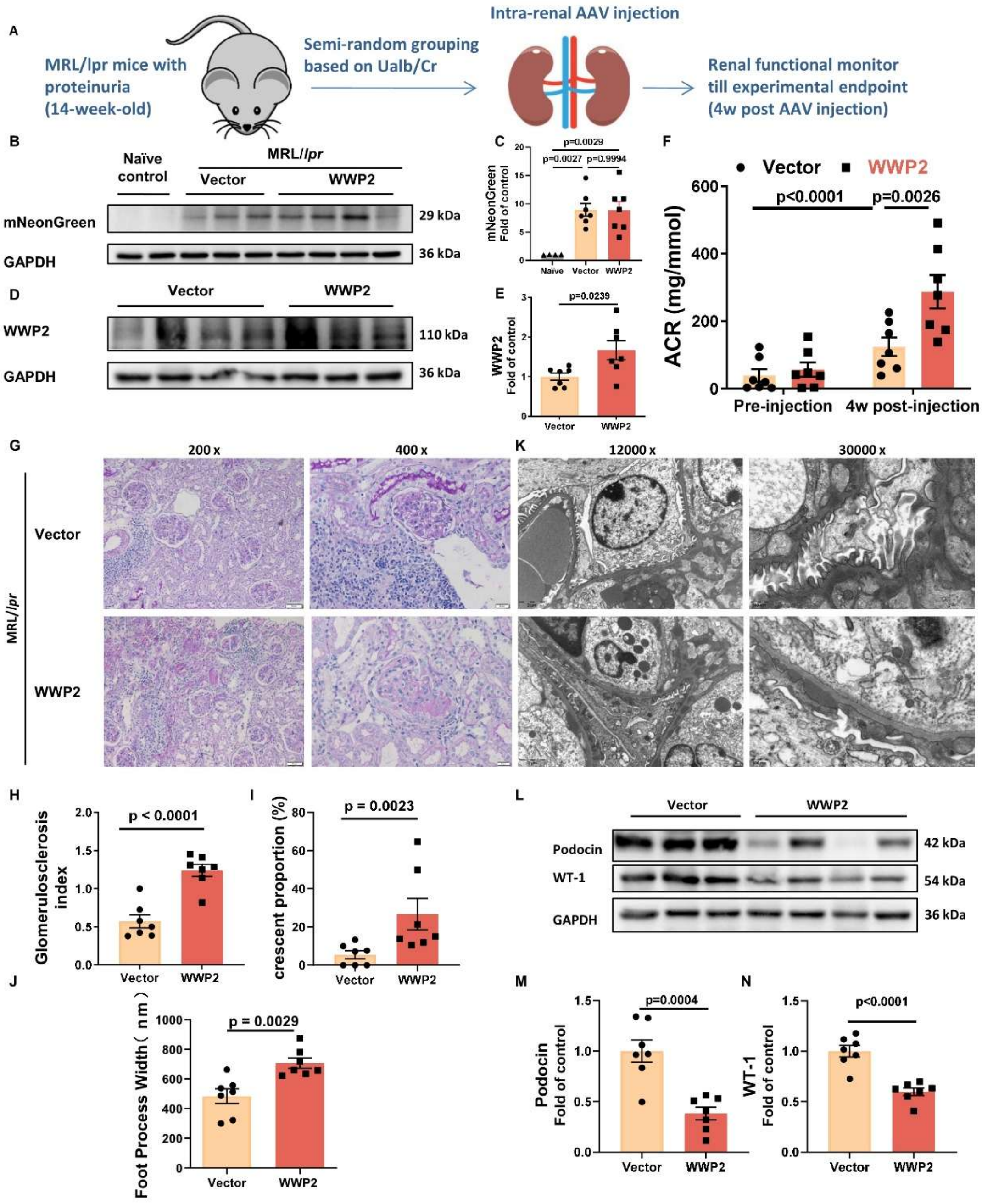

Figure S1 WWP2 Exacerbated Proteinuria and Podocyte Injury in MRL/lpr Mice (A) The experimental scheme. Fourteen-week-old MRL/lpr mice received intra-renal injection of WWP2 AAV2/9 or vector AAV9. The WWP2 AAV9 contained

WWP2 overexpression plasmid constructed on a vector with mNeonGreen. WWP2 co-express with mNeonGreen in a non-fused manner. The mice were semi-randomly assigned to two groups based on their kidney function tested before injection. The kidney function of the mice was indicated by the ACR in the instant urine before AAV injection and at experimental endpoint, i.e., 4 weeks after the AAV injection. The mice were then euthanized for sampling and analysis. n=7 for each group. (**B-E**) Overexpression efficacy on the kidney tissue collected 4 weeks post-injection showed that mNeonGreen, the exogenous tag-gene from AAV2/9 (**B-C**) and WWP2 (**D-E**) significantly overexpressed in the mice kidneys. **B&D**: Representative blots of mNeonGreen and WWP2 in the kidneys of MRL/lpr mice injected with vector or WWP2 AAV9; Naïve control means kidney samples collected from mice that did not receive AAV9 injection, n=4 for Naïve control group and n=7 for the rest two groups; **C&E**: Quantification. (**F**) WWP2 overexpression significantly increased the ACR in the instant urine collected from MRL/lpr mice 4 weeks post AAV injection. (**G-I**) WWP2 aggravated the glomerular lesion as represented by glomerulosclerosis (**H**) and crescent formation (**I**) in the kidneys of MRL/lpr mice. **G**: Representative images of PAS staining. Left: 200 X magnification, scale bars show 50 μm; right: 400 X magnification, scale bars show 20 μm. **H-I**: WWP2 further increased glomerulosclerosis index (**H**) and crescent proportion (**I**). (**J-K**) WWP2 aggravated podocyte injury as represented by foot process loss and effacement in the kidneys of MRL/lpr mice. **J**: Foot process width quantification. **K**: Scale bars in the left and right panels represent 2 μm (12,000 X) and 500 nm (30,000 X), respectively. (**L-N**) WWP2

significantly decreased podocyte markers, such as podocin and WT-1 in the kidneys of MRL/lpr mice as examined by Western blotting analysis. **L**: Representative blots; **M-N**: Quantification results. Bar charts are presented as mean + SEM. P-values for two- or multiple-group comparisons were calculated by Two-tailed unpaired Student's *t*-test or Mann-Whitney test (**I**) and ANOVA followed by Tukey's (**C**) or Sidak's (**F**) post-hoc examination, respectively.

WWP2 knockdown alleviated, while overexpression aggravated tubular injury in MRL/lpr mice

Tubulointerstitial injury, characterized by tubular injury and interstitial inflammation and fibrosis, is an essential pathology in LN kidneys, which may be secondary to proteinuria and glomerular lesion. In the lpr mice kidneys, we found that WWP2 knockdown with AAV significantly decreased NGAL and tubulointerstitial injury score quantified by the PAS staining shown in **Figure 2E**; WWP2 overexpression increased NGAL and tubulointerstitial injury score (PAS images shown in **Figure S1G**). These results were consistent with those in glomerular lesions, demonstrating WWP2's pathological implication in LN.

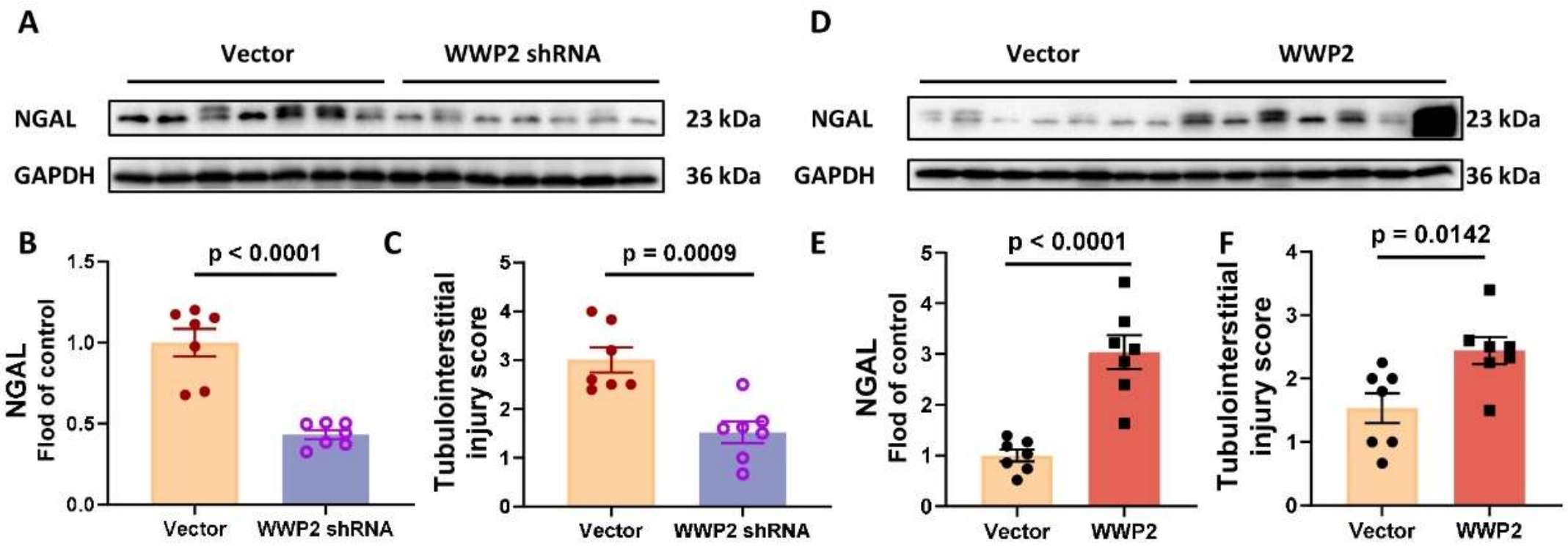

Figure S2 WWP2 knockdown alleviated, while overexpression aggravated tubular injury in MRL/lpr mice Kidney tissue homogenate or PAS staining images from WWP2 shRNA or WWP2 AAV injected MLR/lpr mice as indicated in **Figure 2 & S1** were analyzed for NGAL and tubulointersitial injury pathology. n=7. (**A-C**) WWP2 knockdown decreased NGAL and tubulointerstitial injury score in MRL/lpr mice. (**D-F**) WWP2 increased NGAL and tubulointerstitial injury score in MRL/lpr mice. Two-tailed *t*-test were used for statistical analysis.

H2A did not change in MRL/lpr mice kidneys, nor in HPC upon WWP2 manipulation

We isolated the histone fraction from the kidneys of MRL/lpr mice and the genetic background control. Ponceau S staining of the uncropped full membrane showed protein bands in the 15-17 kDa. Antibody probing showed that H2A did not change in the MRL/lpr mice kidneys (**Figure S3A-B**). We also isolated the histone fraction from the HPC and found that H2A did not change in those with WWP2 KO or overexpression (**Figure S3C-E**).

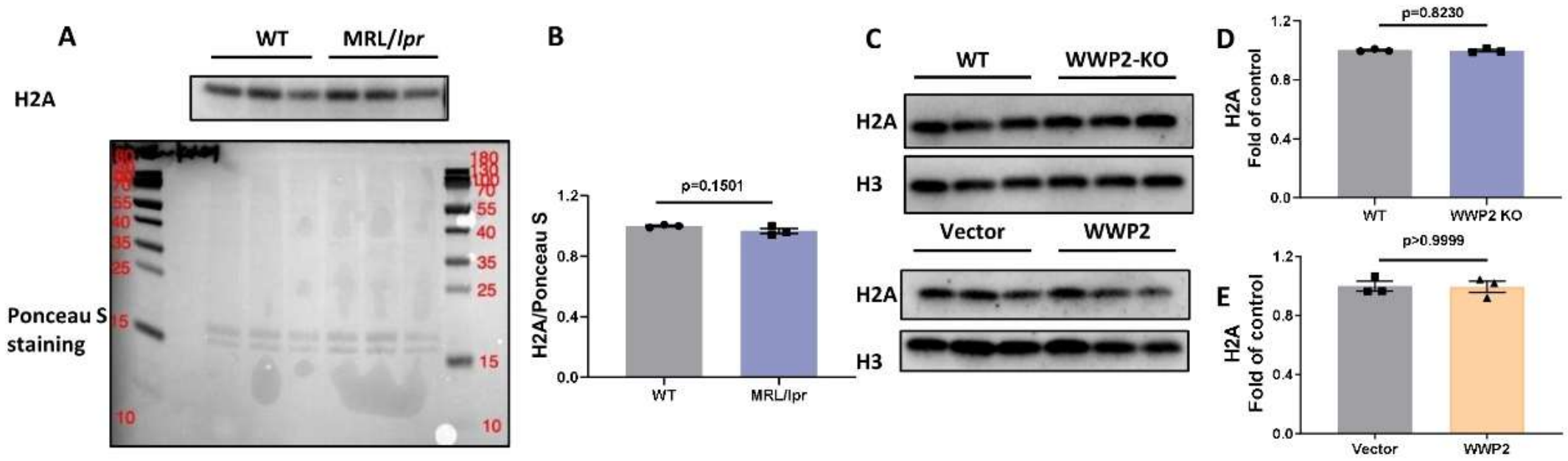

Figure S3 H2A did not change in MRL/lpr mice kidneys, nor in HPC upon WWP2 manipulation. (**A-B**) H2A did not change in MRL/lpr mice kidneys. Histone fraction was isolated from the MRL/lpr mice and MRL/MPJ mice, the genetic background. Ponceau S staining on the freshly transmembrane after electropherosis showed that the protein bands were predominantly located in 15-17 kDa. H2A antibody detection showed no change in H2A in MRL/lpr mice. n=3. (**C-E**) H2A did not change in WWP2 KO or overexpression HPCs. n=3 biological replicates. P-values for **B** & **D** were calculated by Two-tailed unpaired Student's t-test. P-value for **E** were by Non-parametric Mann-Whitney test.

H2A siRNA knockdown H2A in HPC

H2A siRNA were transfected in the 12-day differentiated HPC and the histones were extracted for knockdown efficiency examination by western blotting analysis (Figure S4).

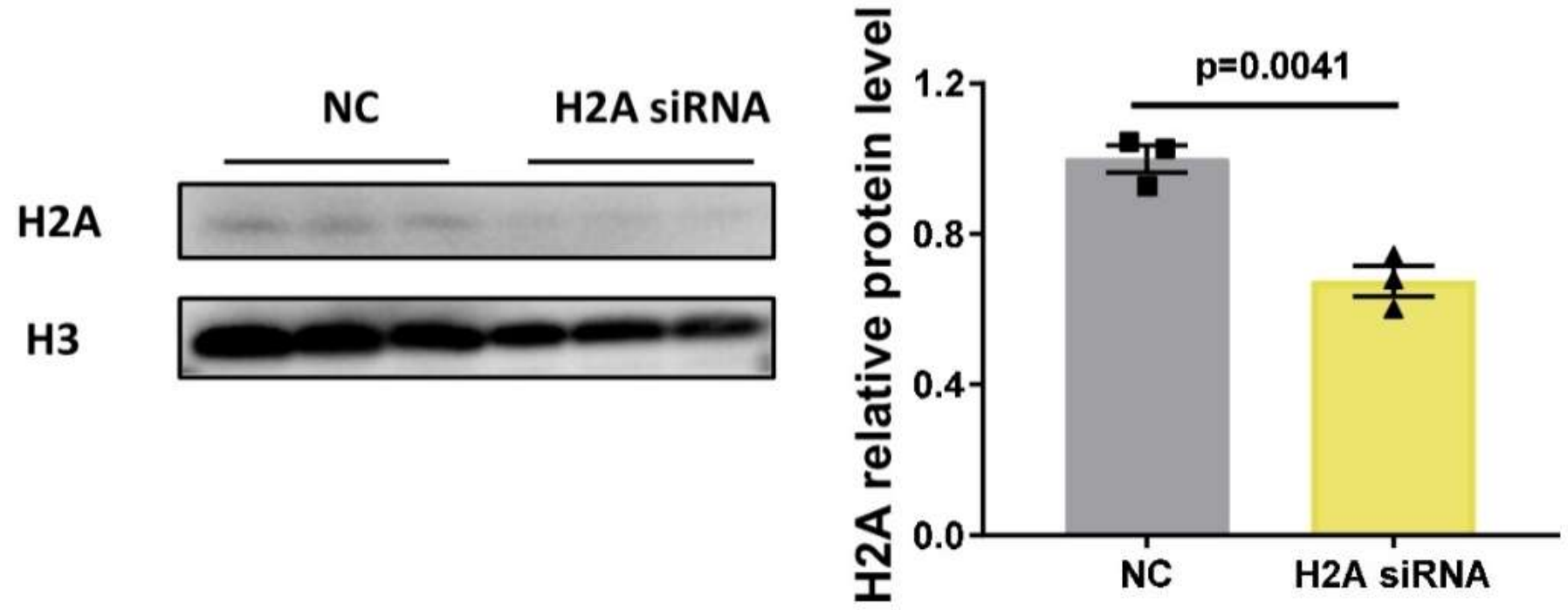

Figure S4 H2A siRNA knockdown H2A in HPC HPC were differentiated for 12 days before transfected with H2A siRNA and the histone fraction was isolated for knockdown efficiency validation using western blotting analysis. n=3 biological replicates. Two-tailed unpaired Student's t-test were used for statistical analysis.

References

1. Bai M, Wu M, Jiang M, He J, Deng X, Xu S, et al. LONP1 targets HMGCS2 to protect mitochondrial function and attenuate chronic kidney disease. EMBO Mol Med. 2023; 15: e16581.

2. Smeets B, Kabgani N, Moeller MJ. Isolation and Primary Culture of Murine Podocytes with Proven Origin. Methods Mol Biol. 2016; 1397: 3-10.

3. Katsuya K, Yaoita E, Yoshida Y, Yamamoto Y, Yamamoto T. An improved method for primary culture of rat podocytes. Kidney Int. 2006; 69: 2101-6.

4. Chung JJ, Goldstein L, Chen YJ, Lee J, Webster JD, Roose-Girma M, et al. Single-Cell Transcriptome Profiling of the Kidney Glomerulus Identifies Key Cell Types and Reactions to Injury. J Am Soc Nephrol. 2020; 31: 2341-54.

5. Hoppe JM, Vielhauer V. Induction and analysis of nephrotoxic serum nephritis in mice. Methods Mol Biol. 2014; 1169: 159-74.

6. Ma H, Togawa A, Soda K, Zhang J, Lee S, Ma M, et al. Inhibition of podocyte FAK protects against proteinuria and foot process effacement. J Am Soc Nephrol. 2010; 21: 1145-56.

7. Laboratory J. Phenotypes of MRL-lpr & MRL Mice.

8. Furusho T, Das R, Hakui H, Sairavi A, Adachi K, Galbraith-Liss MS, et al. Enhancing gene transfer to renal tubules and podocytes by context-dependent selection of AAV capsids. Nat Commun. 2024; 15: 10728.
9. You R, Li Y, Jiang Y, Hu D, Gu M, Zhou W, et al. WWP2 deletion aggravates acute kidney injury by targeting CDC20/autophagy axis. J Adv Res. 2024.
10. Rodriguez-Iturbe B, Quiroz Y, Shahkarami A, Li Z, Vaziri ND. Mycophenolate mofetil ameliorates nephropathy in the obese Zucker rat. Kidney Int. 2005; 68: 1041-7.
11. Bajema IM, Wilhelmus S, Alpers CE, Bruijn JA, Colvin RB, Cook HT, et al. Revision of the International Society of Nephrology/Renal Pathology Society classification for lupus nephritis: clarification of definitions, and modified National Institutes of Health activity and chronicity indices. Kidney Int. 2018; 93: 789-96.
12. Li F, Liang Z, Zhong H, Hu X, Tang Z, Zhu C, et al. Group 3 Innate Lymphoid Cells Exacerbate Lupus Nephritis by Promoting B Cell Activation in Kidney Ectopic Lymphoid Structures. Adv Sci (Weinh). 2023; 10: e2302804.
13. Tain YL, Hsieh CS, Chen CC, Sheen JM, Lee CT, Huang LT. Melatonin prevents increased asymmetric dimethylarginine in young rats with bile duct ligation. J Pineal Res. 2010; 48: 212-21.
14. Wu M, Miao M, Li Y, Fan J, Zhu W, Niu Y, et al. Targeting renal tubular WWP2 to restore mitochondrial OXPHOS integrity retards the AKI-to-CKD transition. Mol Ther. 2025.
15. Yu F, Haas M, Glassock R, Zhao MH. Redefining lupus nephritis: clinical implications of pathophysiologic subtypes. Nat Rev Nephrol. 2017; 13: 483-95.
16. Ichinose K, Kitamura M, Sato S, Fujikawa K, Horai Y, Matsuoka N, et al. Podocyte foot process width is a prediction marker for complete renal response at 6 and 12 months after induction therapy in lupus nephritis. Clin Immunol. 2018; 197: 161-8.
17. Chen A, Feng Y, Lai H, Ju W, Li Z, Li Y, et al. Soluble RARRES1 induces podocyte apoptosis to promote glomerular disease progression. J Clin Invest. 2020; 130: 5523-35.
18. Wright RD, Beresford MW. Podocytes contribute, and respond, to the inflammatory environment in lupus nephritis. Am J Physiol Renal Physiol. 2018; 315: F1683-F94.
19. Shukla S, Ying W, Gray F, Yao Y, Simes ML, Zhao Q, et al. Small-molecule inhibitors targeting Polycomb repressive complex 1 RING domain. Nat Chem Biol. 2021; 17: 784-93.

20. Maddika S, Kavela S, Rani N, Palicharla VR, Pokorny JL, Sarkaria JN, et al. WWP2 is an E3 ubiquitin ligase for PTEN. Nat Cell Biol. 2011; 13: 728-33.

Western blotting summary

Figure 1E

WWP2 (110 kDa)

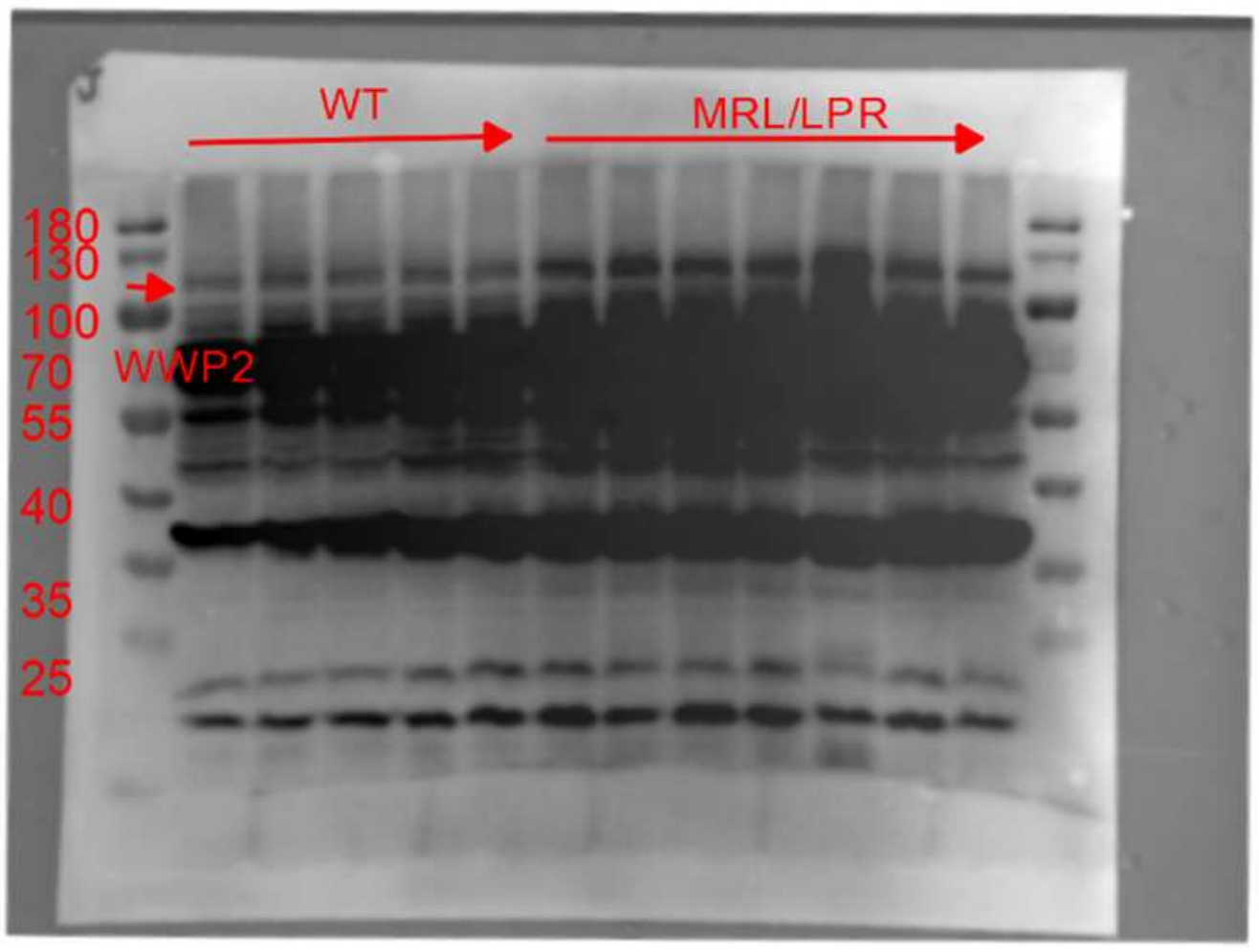

GAPDH (36 kDa)

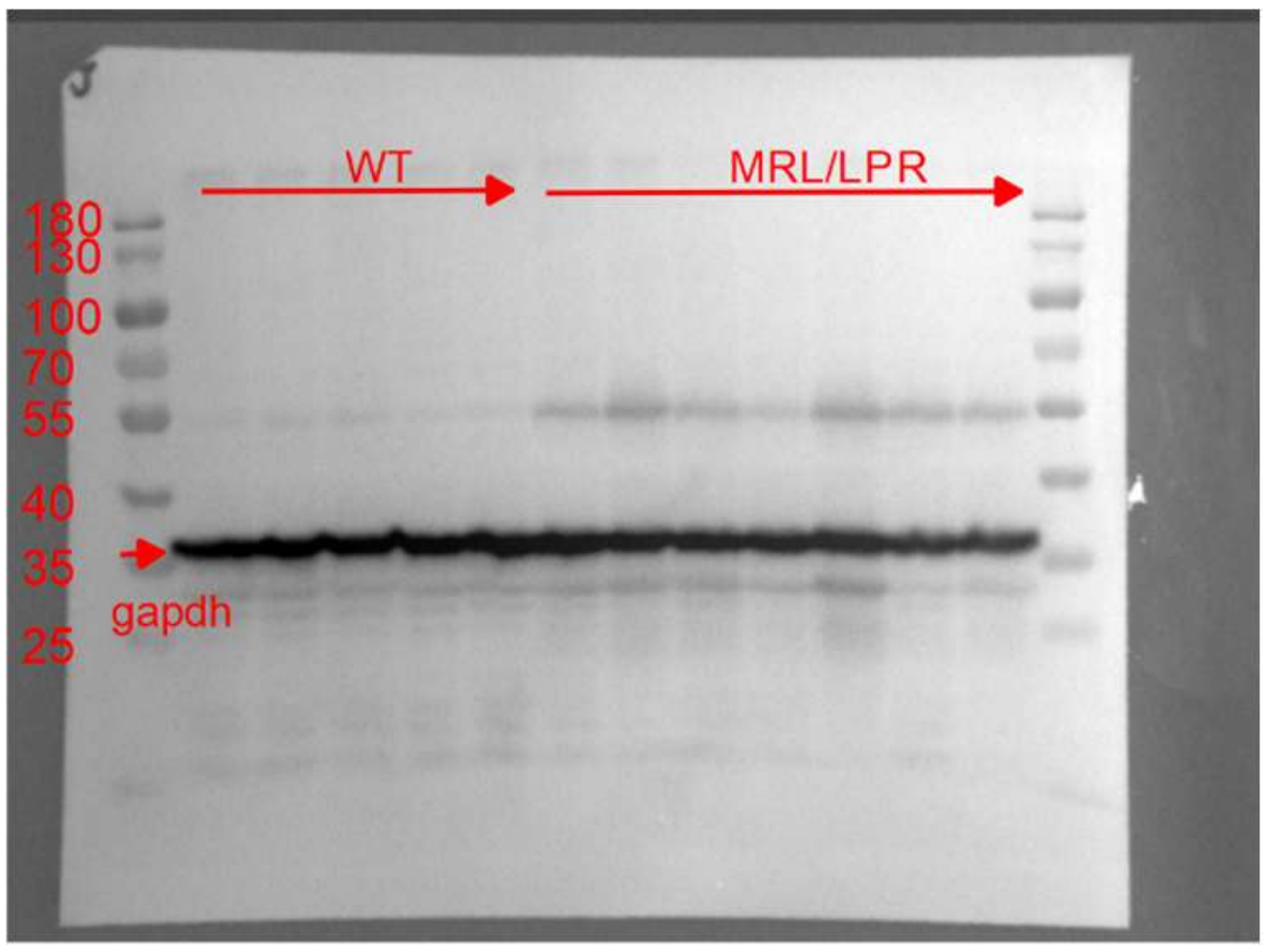

Figure 1G

WWP2 (110 kDa)

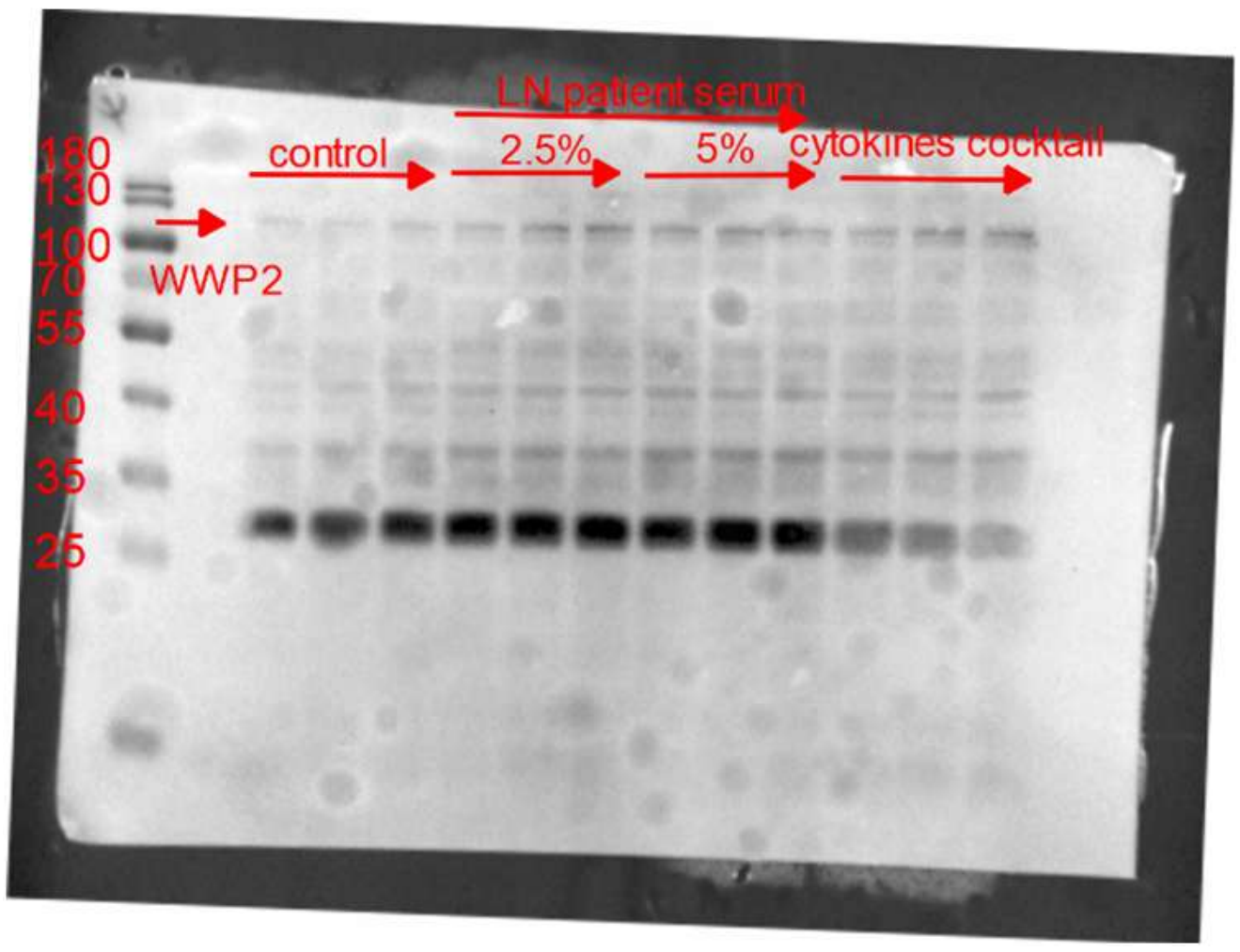

β-actin (42 kDa)

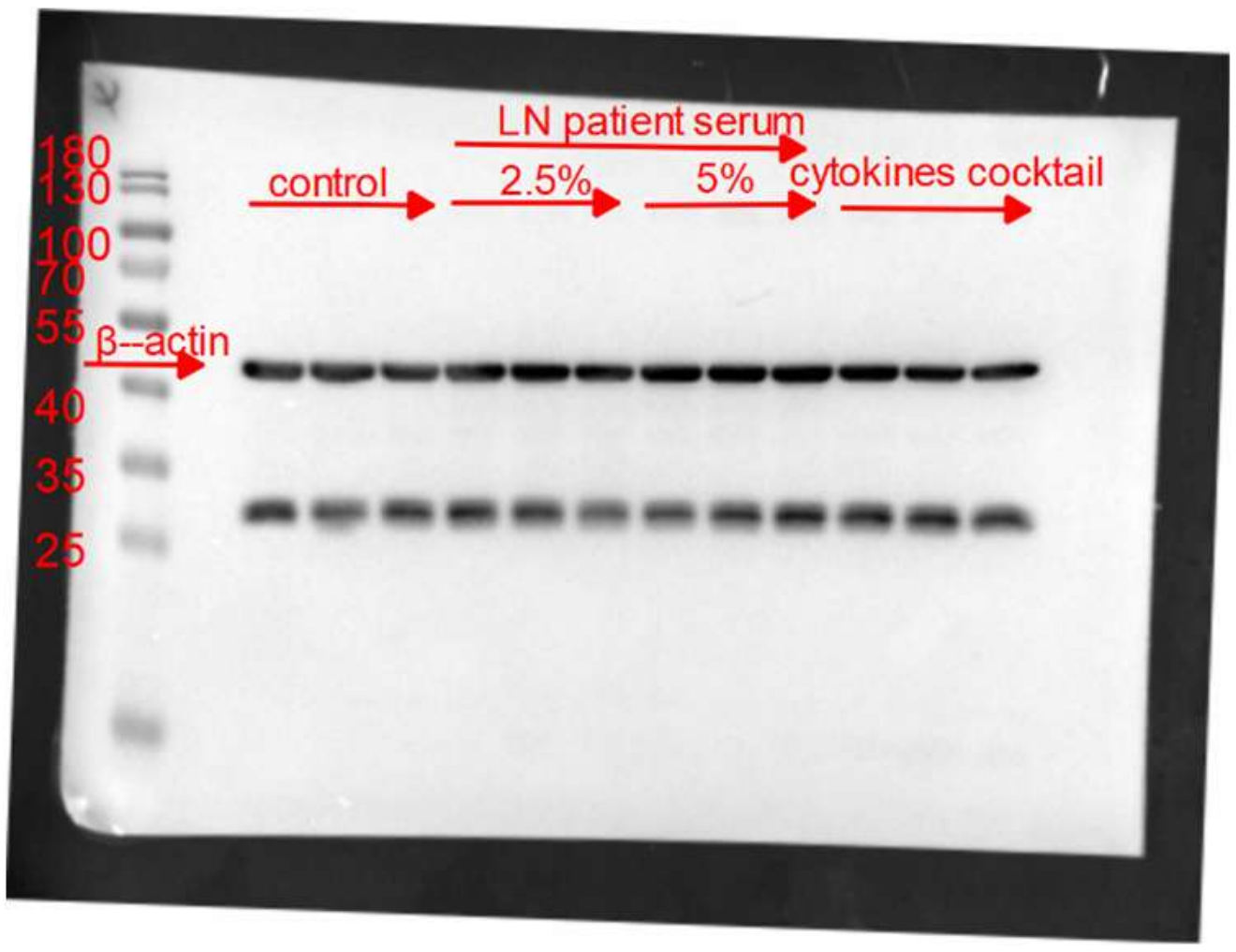

Figure 2B&J

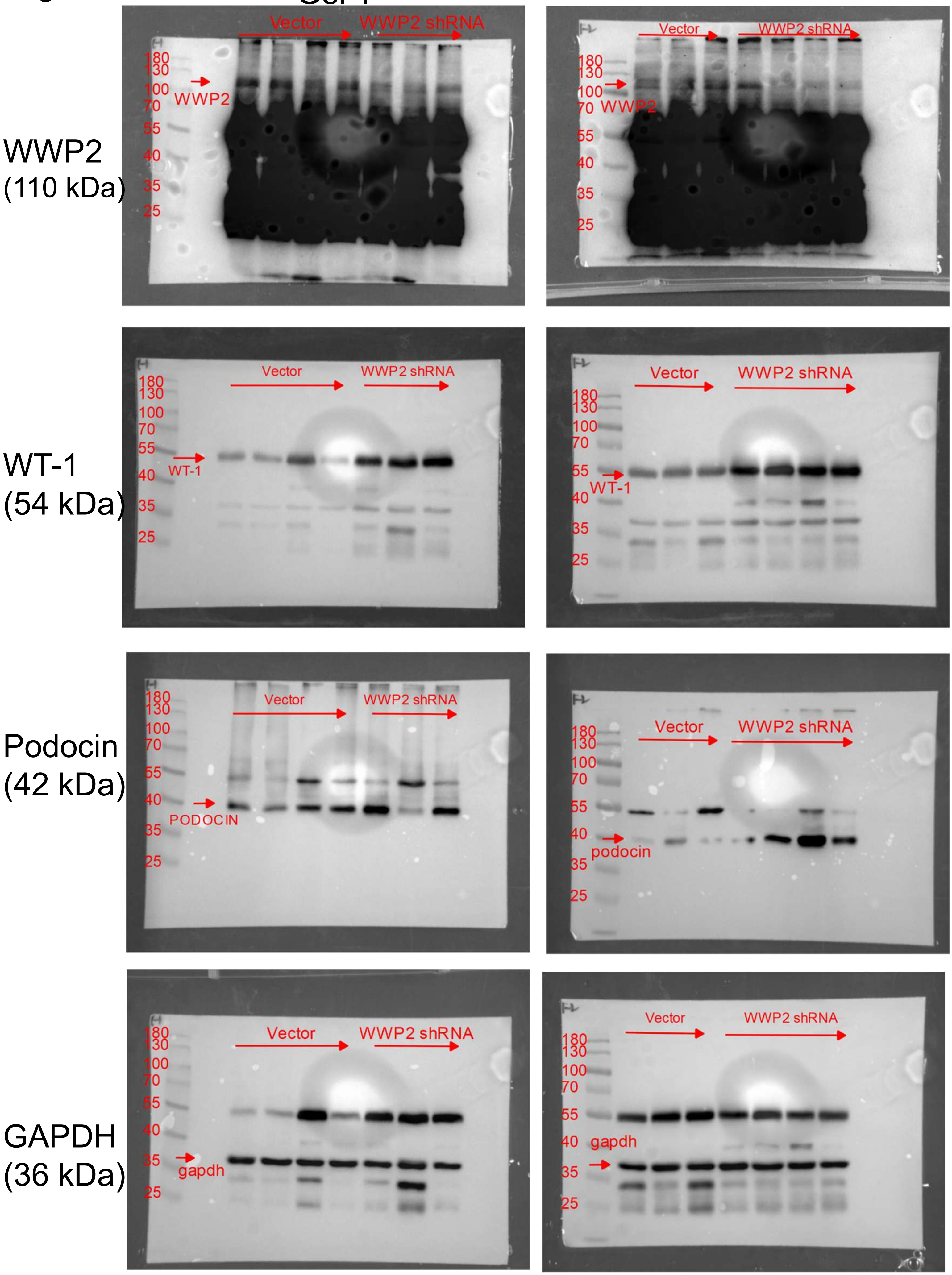

Gel 1
Gel 2
WWP2
(110 kDa)
WT-1
(54 kDa)
Podocin
(42 kDa)
GAPDH
(36 kDa)
Vector
WWP2 shRNA
180
130
100
70
55
40
35
25
WWP2
WT-1
PODOCIN
podocin
gapdh

Figure 3A WWP2 (100 kDa)

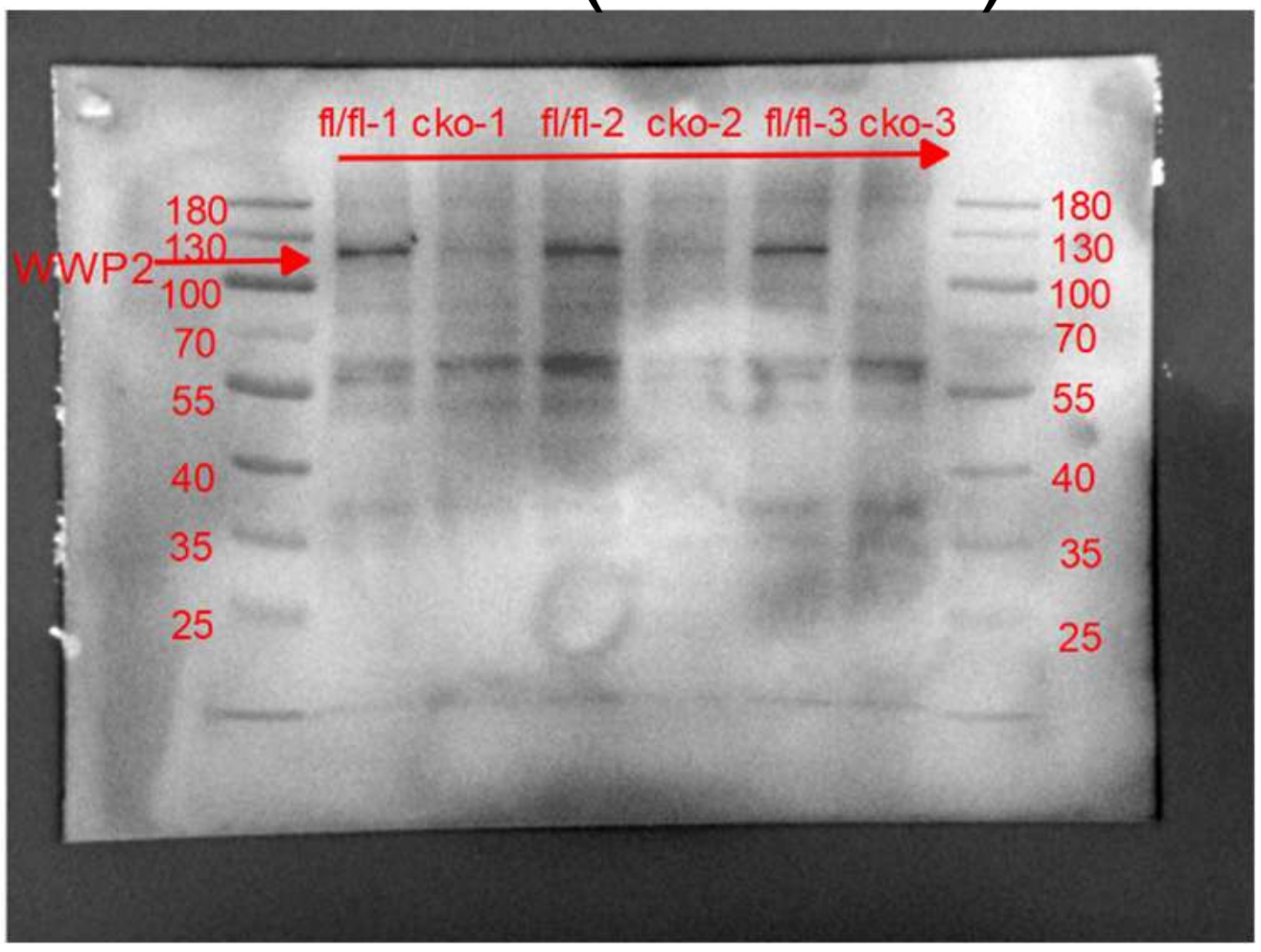

β-actin (42 kDa)

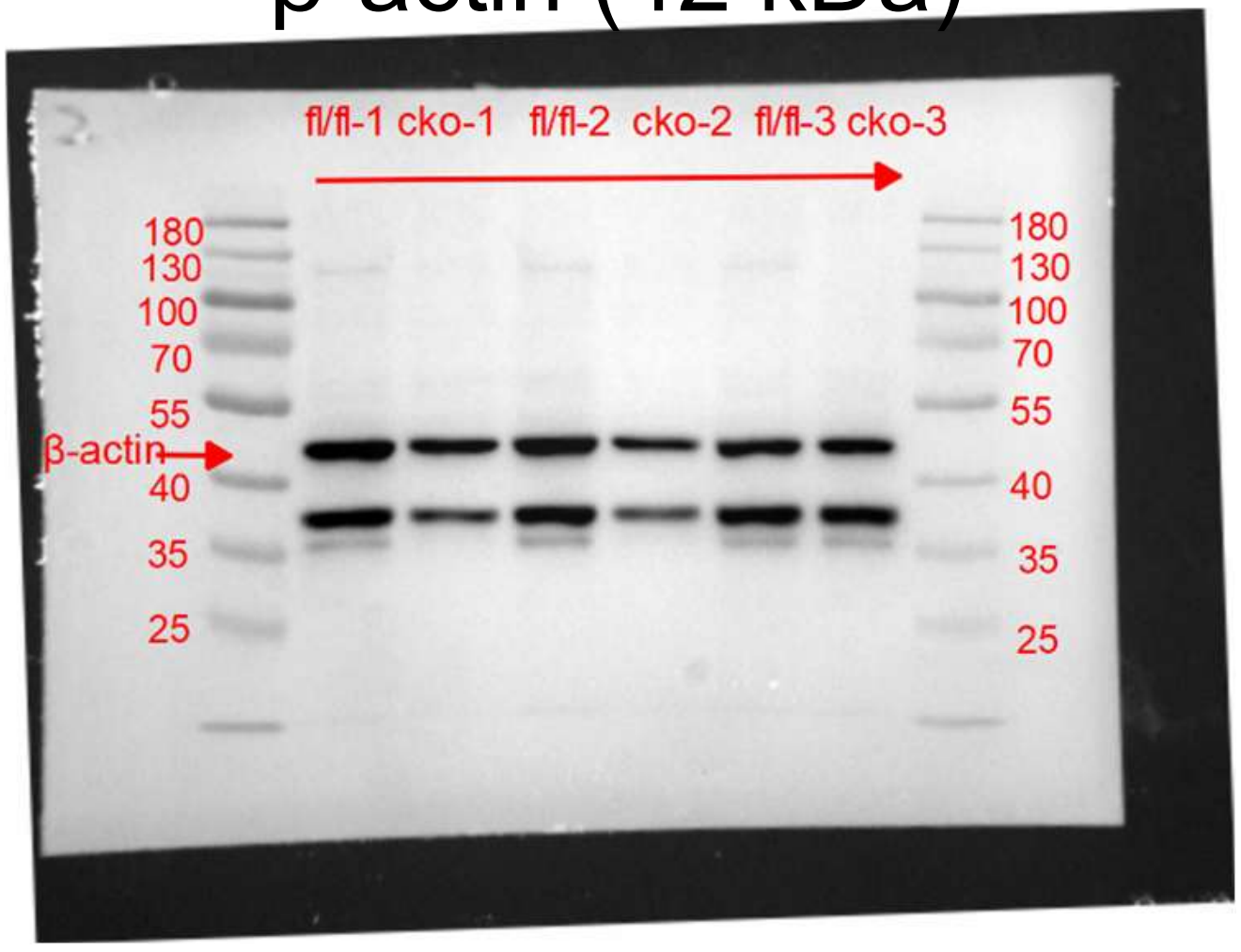

Figure 4A-D WWP2 (110 kDa)

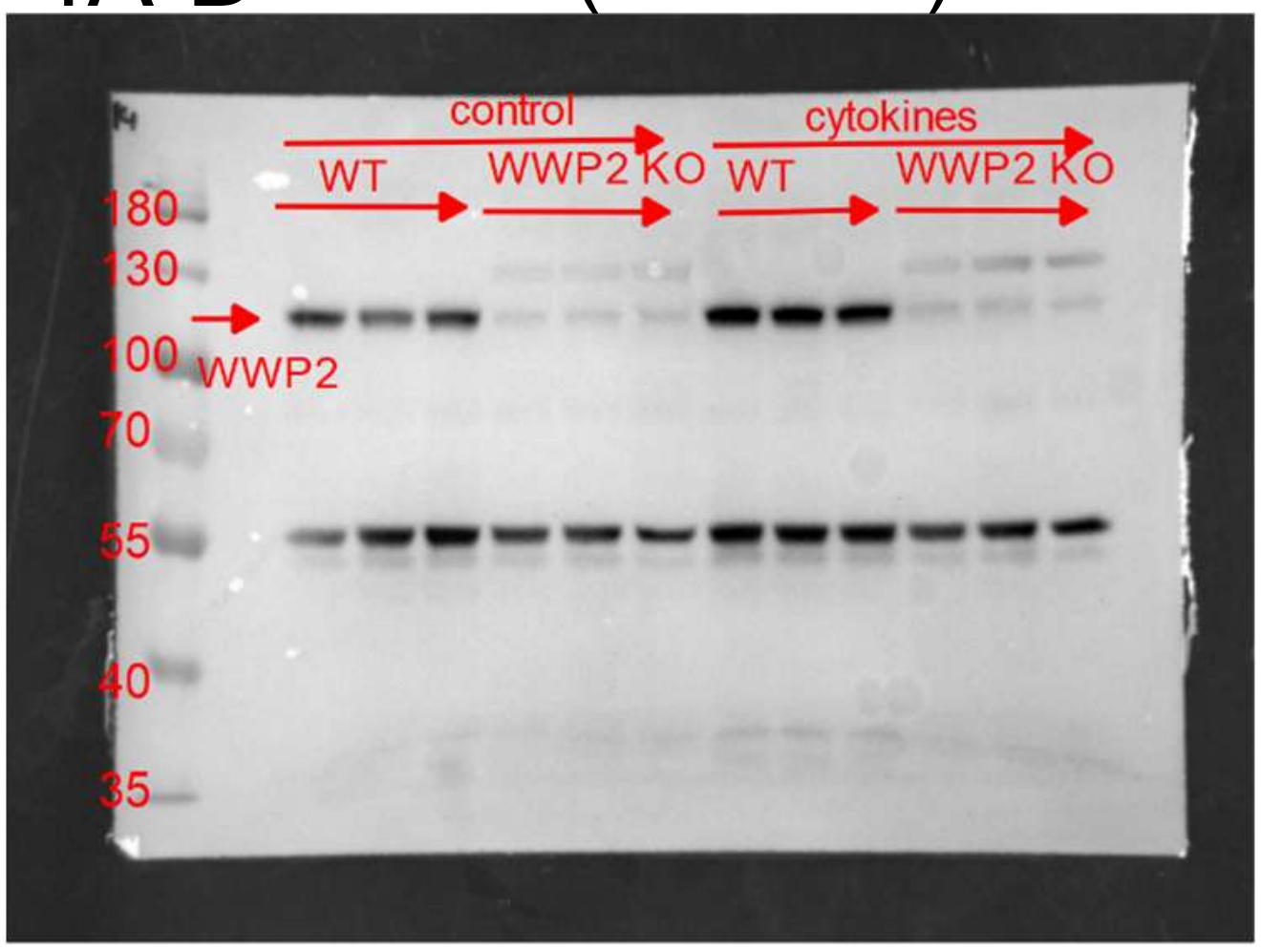

GAPDH (36 kDa)

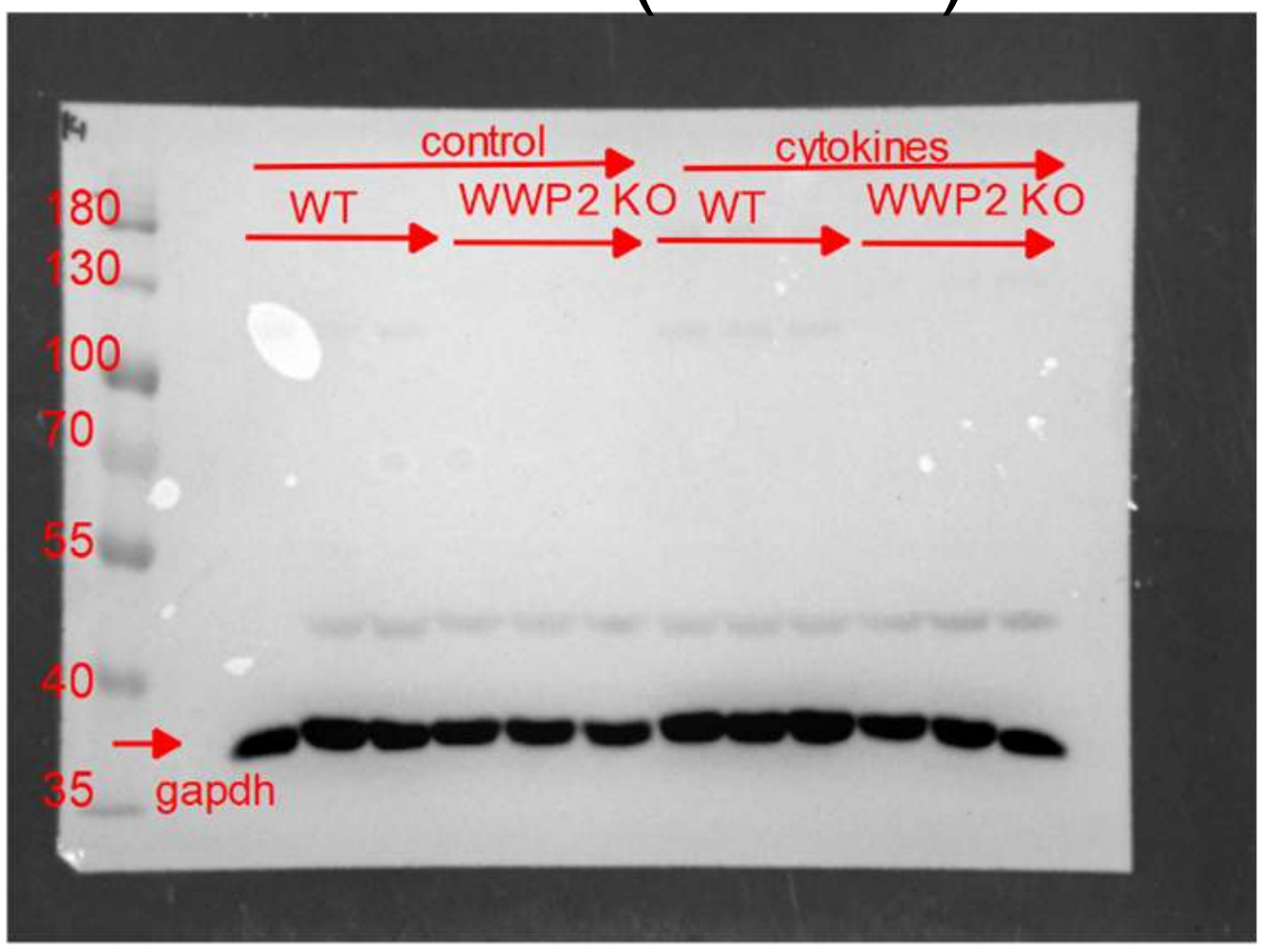

WT-1 (54 kDa)

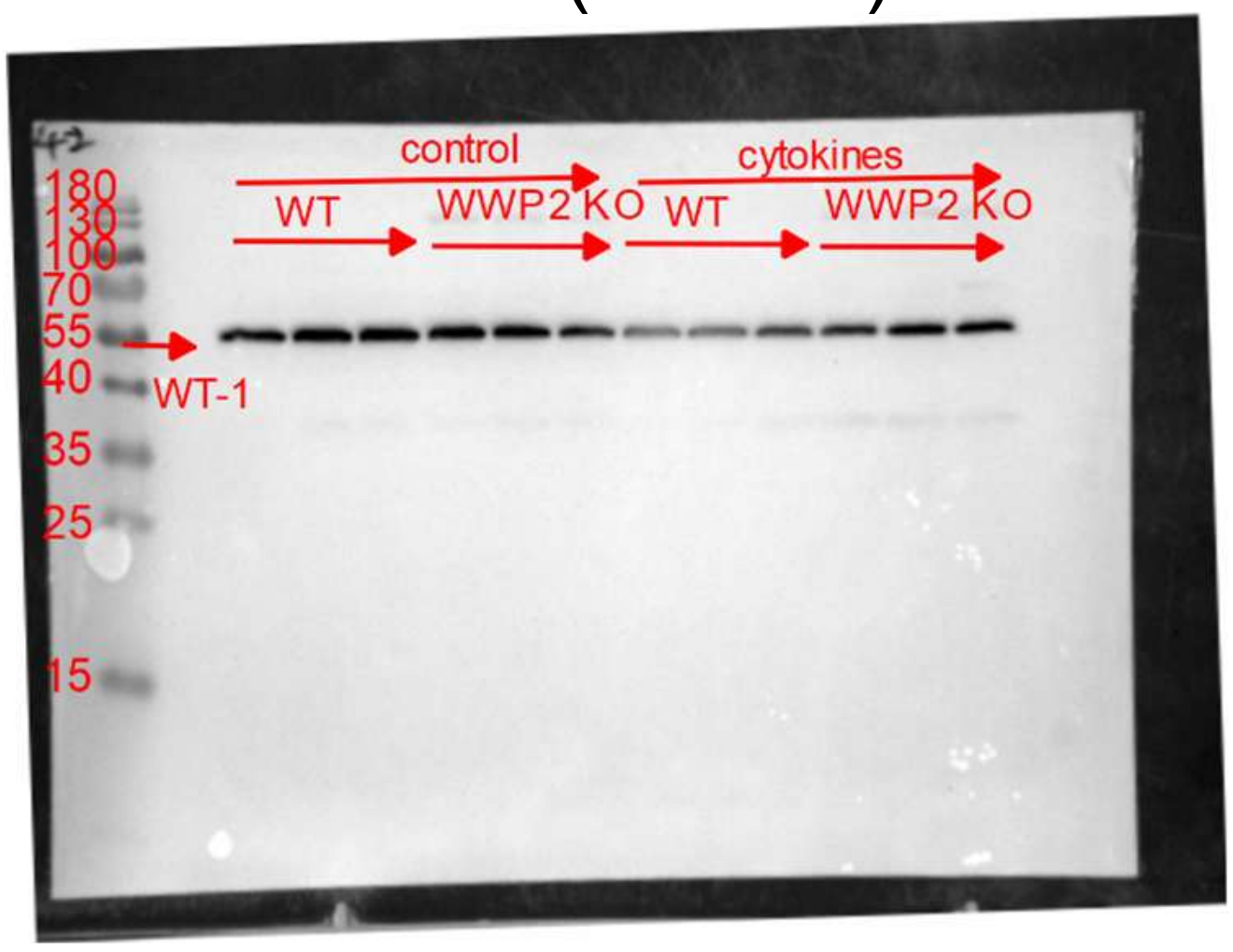

GAPDH (36 kDa)

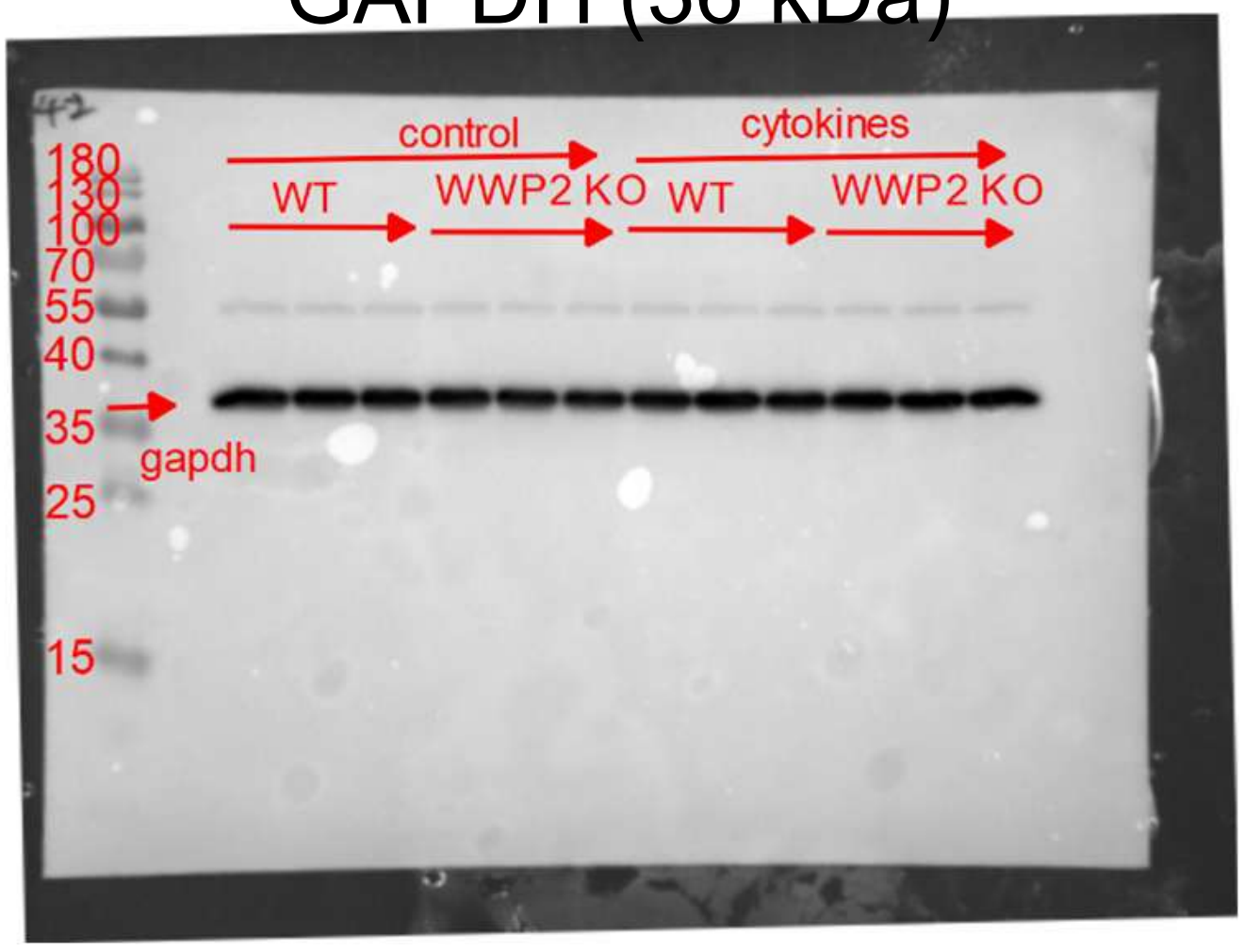

Cleaved caspase-3 (17 kDa)

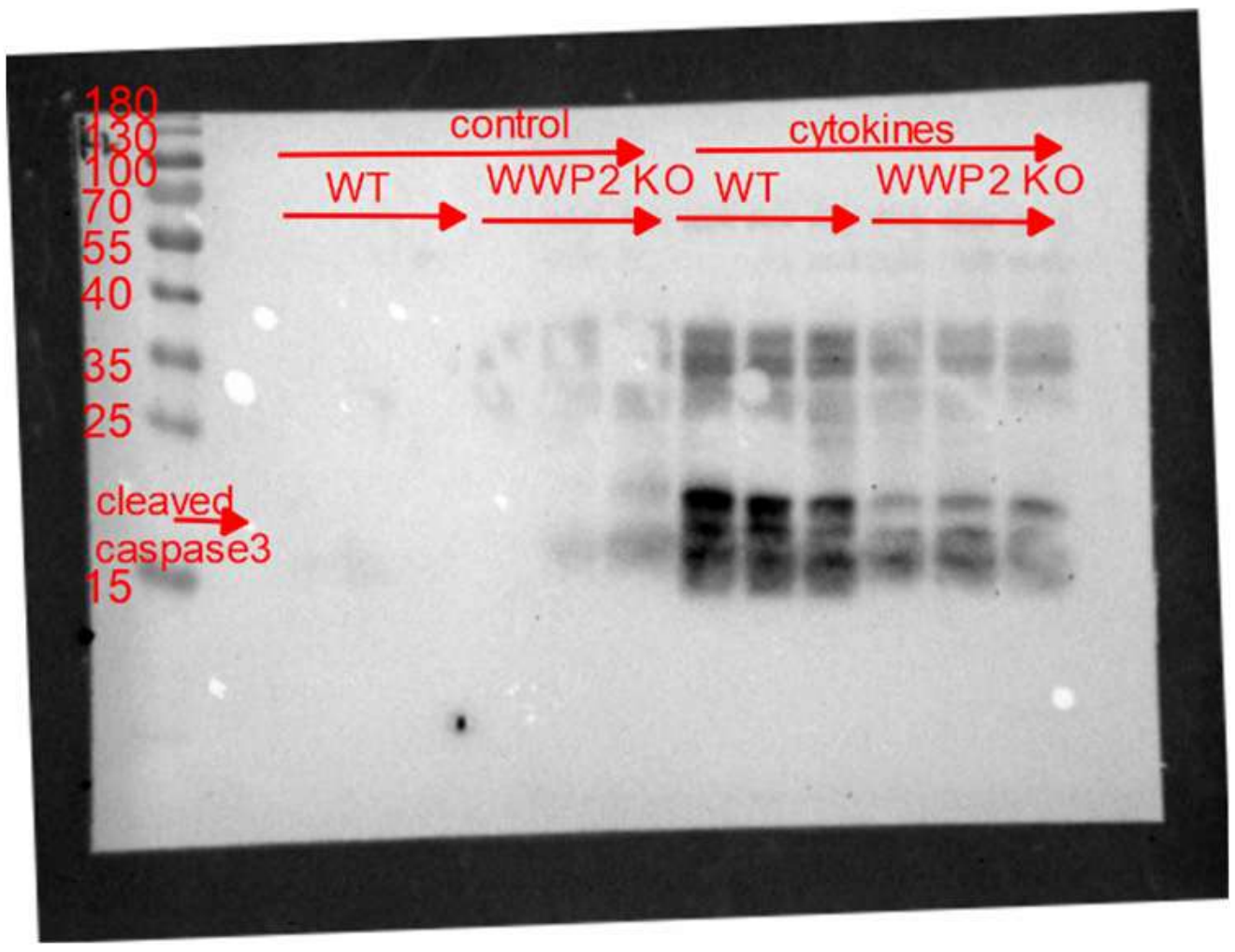

GAPDH (36 kDa)

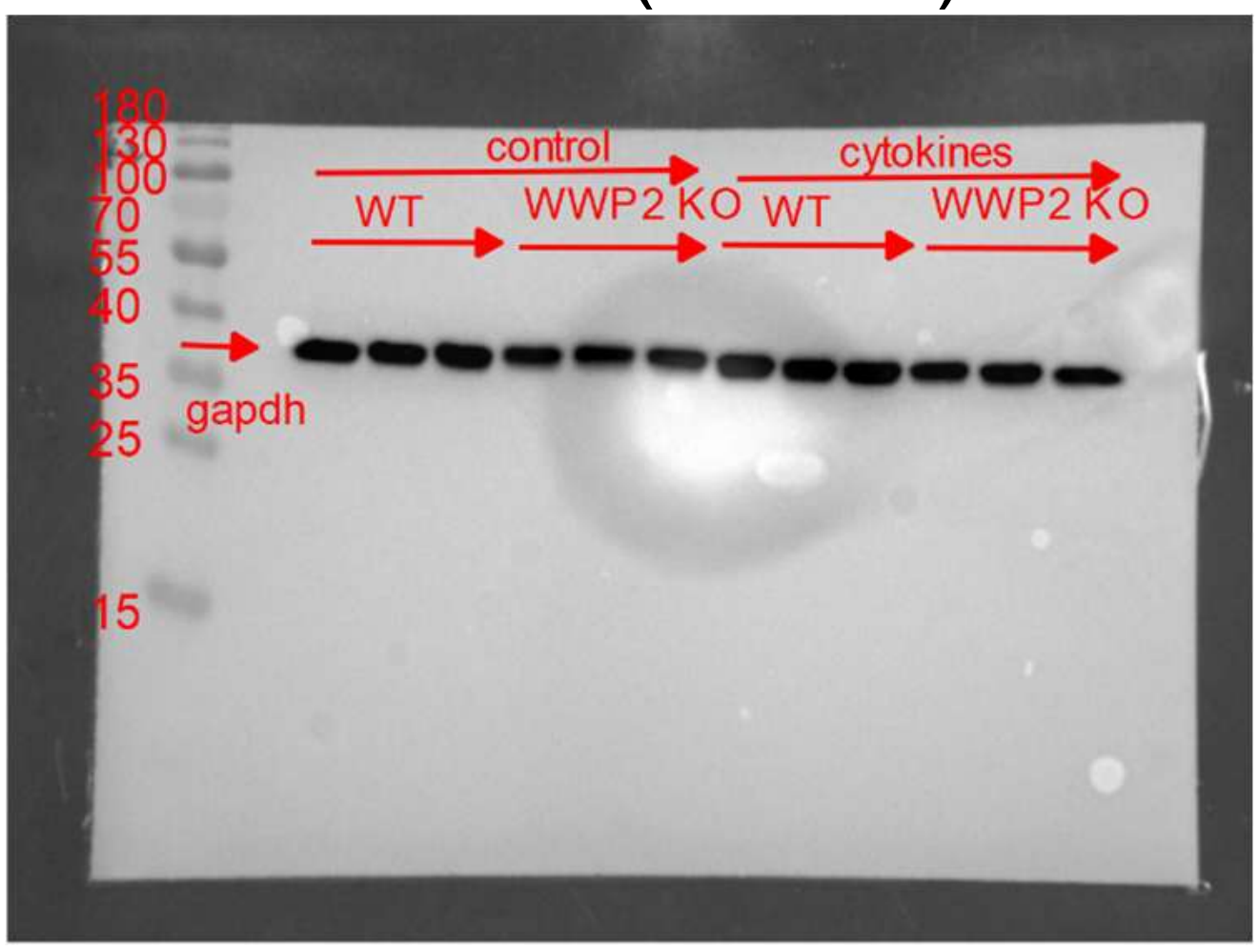

Figure 4F

WWP2 (110 kDa)

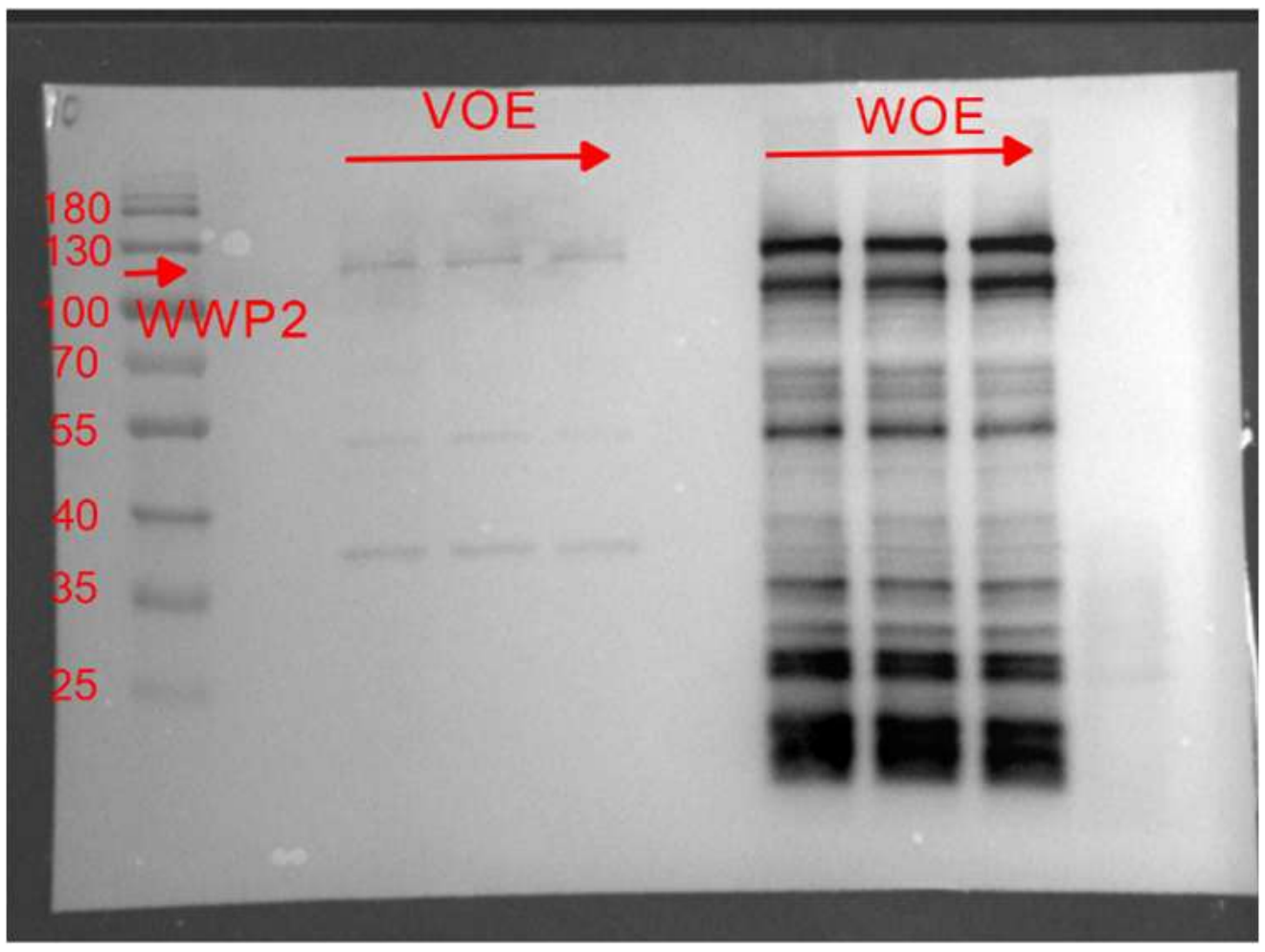

GAPDH (36 kDa)

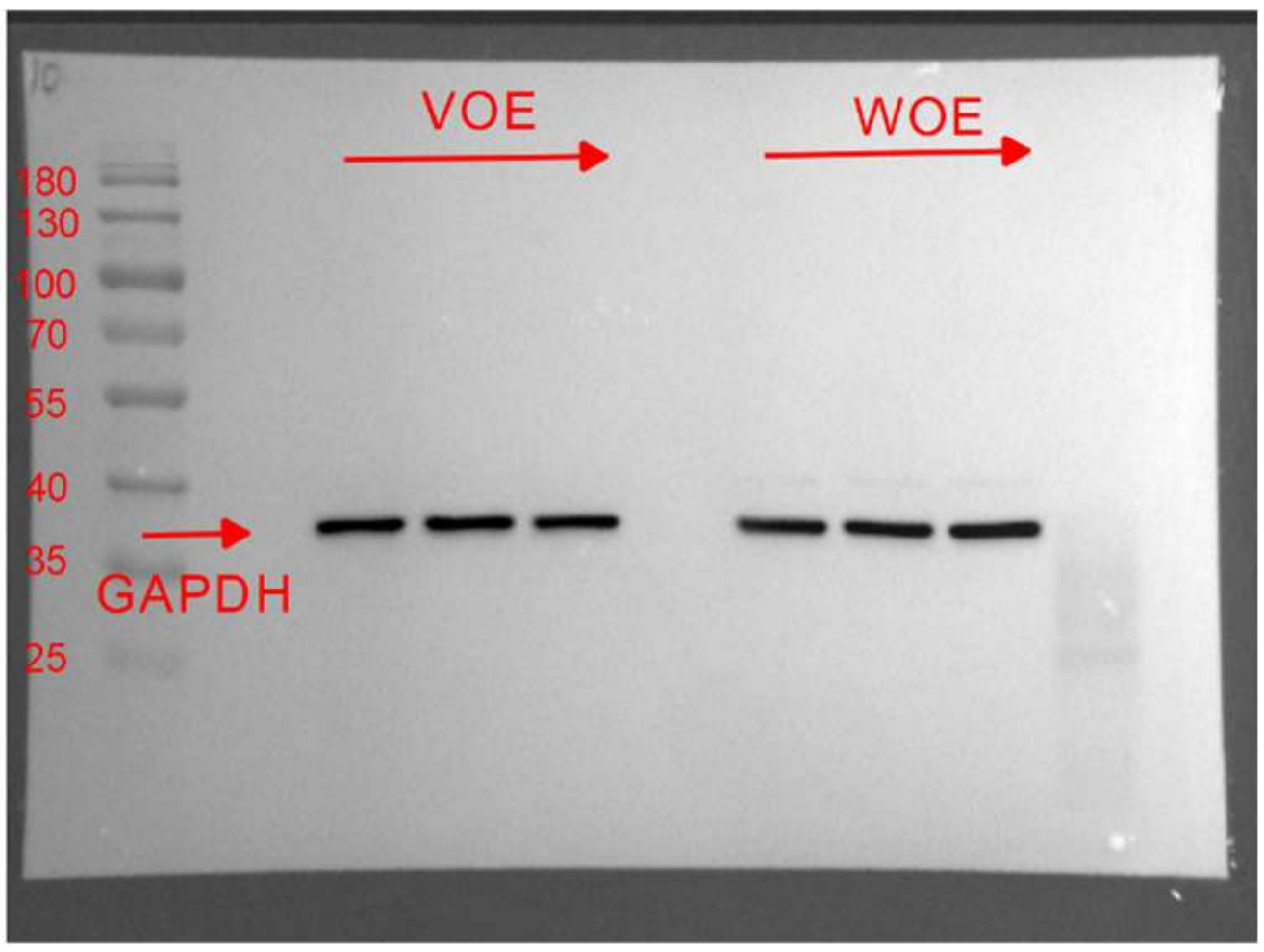

Figure 4H

WT-1 (54 kDa)

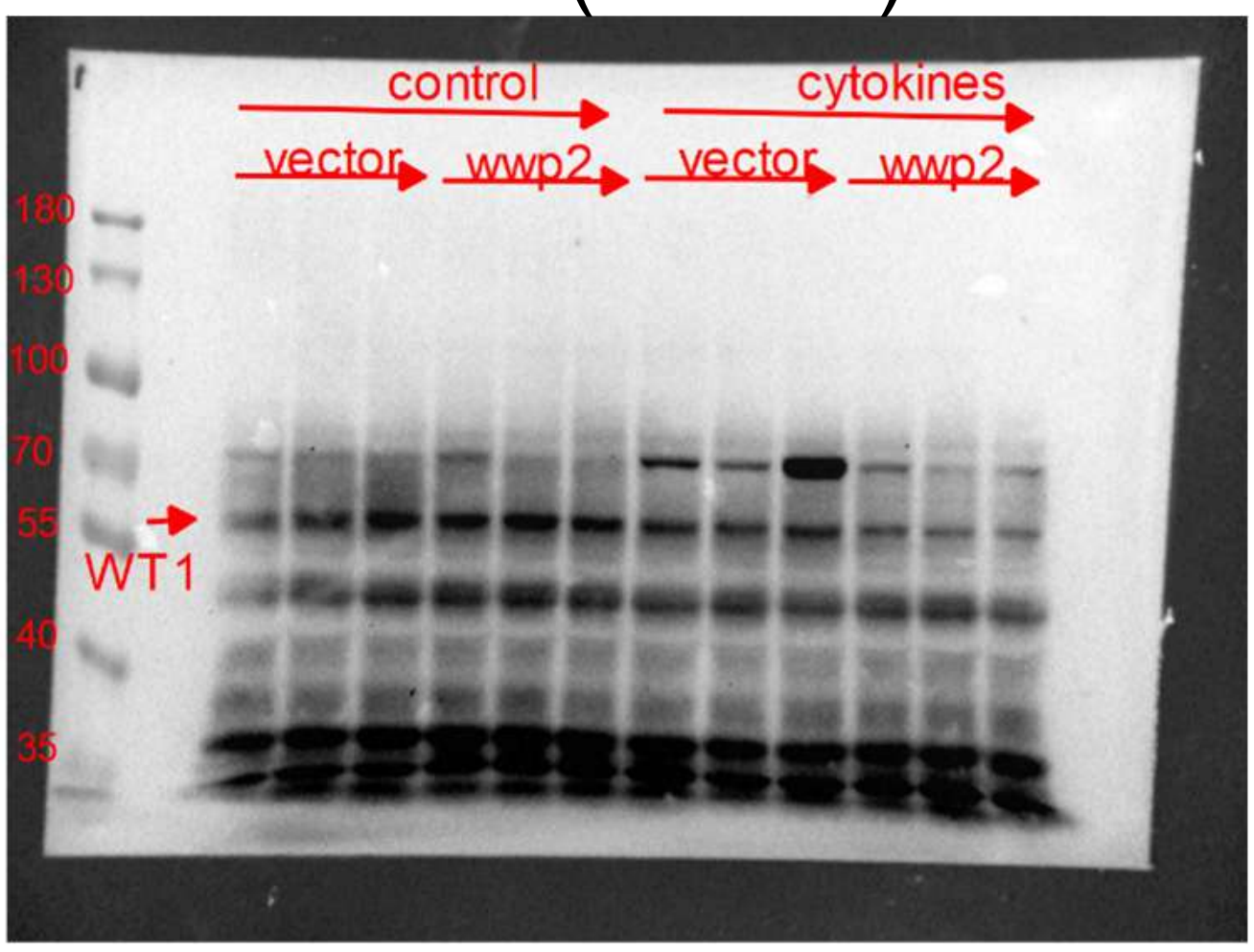

GAPDH (36 kDa)

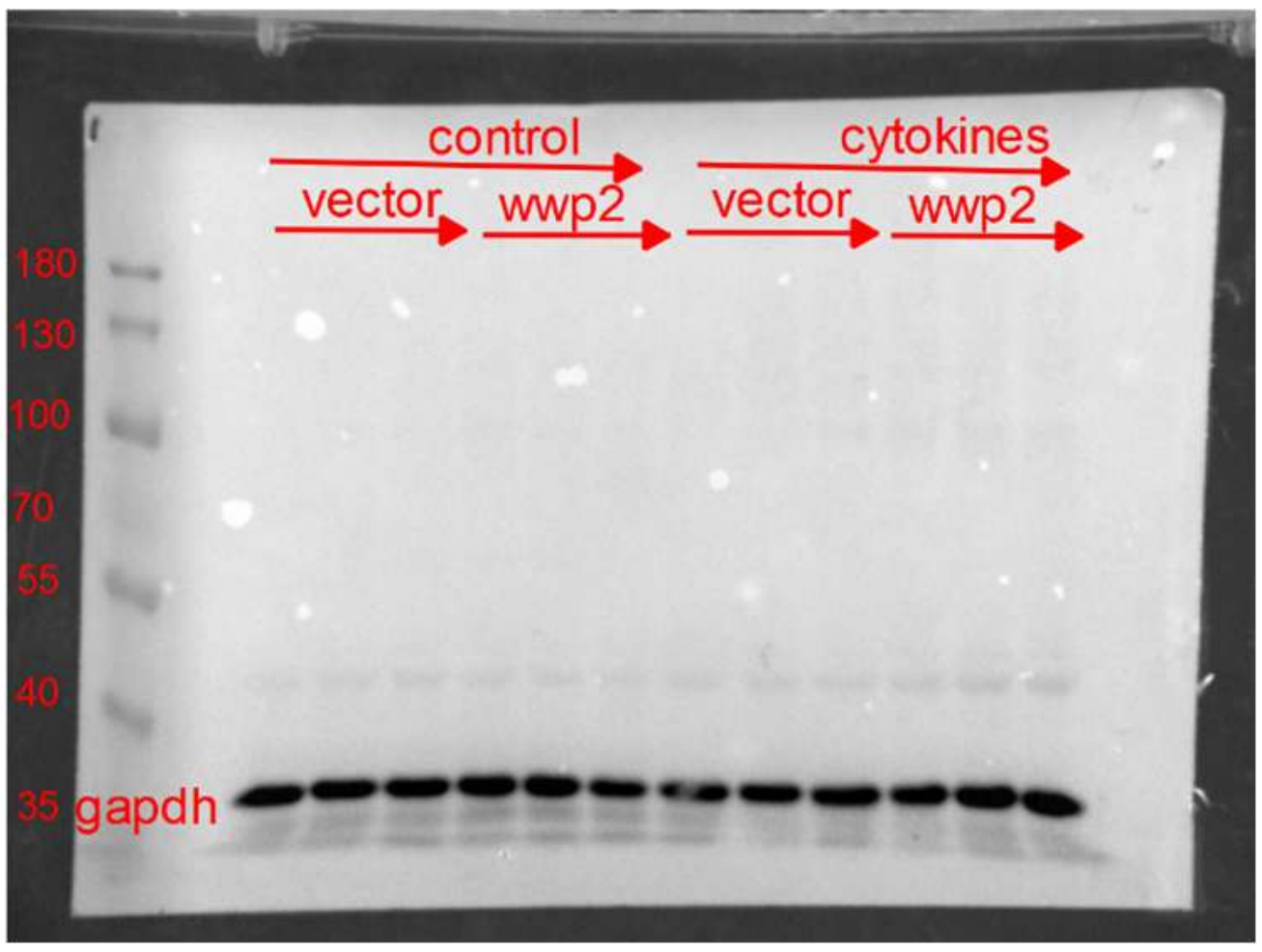

Cleaved caspase-3 (17 kDa)

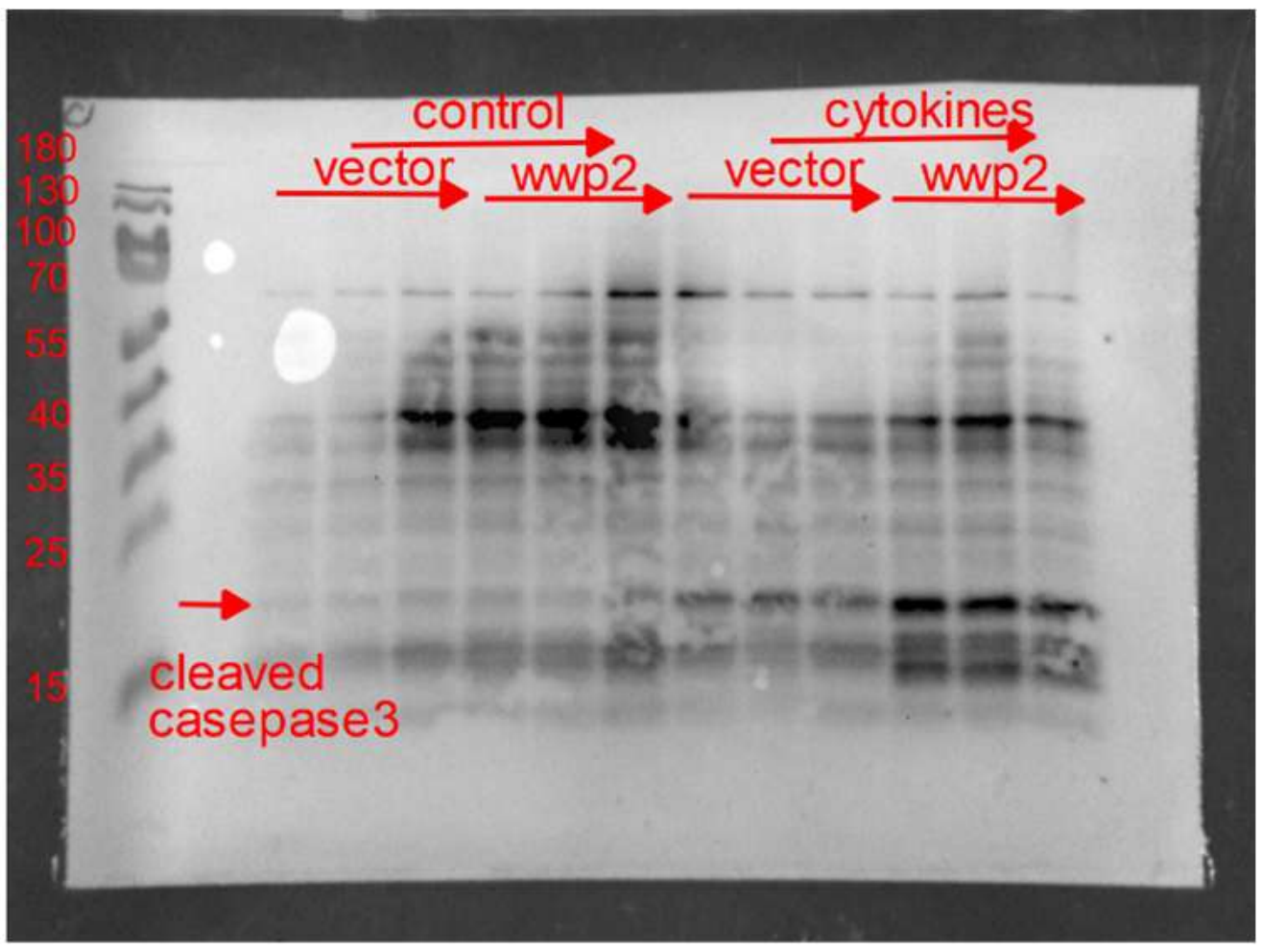

GAPDH (36 kDa)

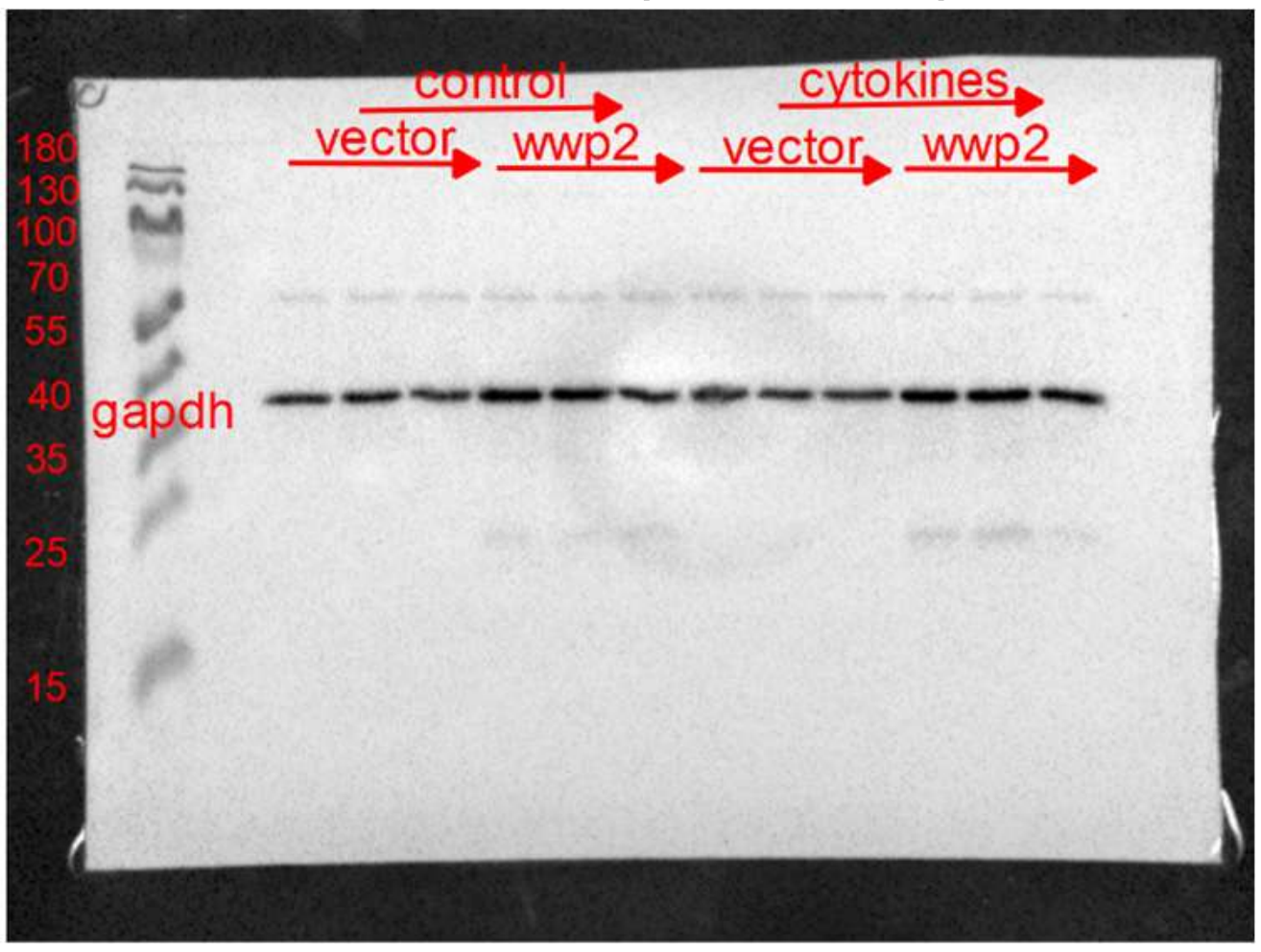

Figure 5D

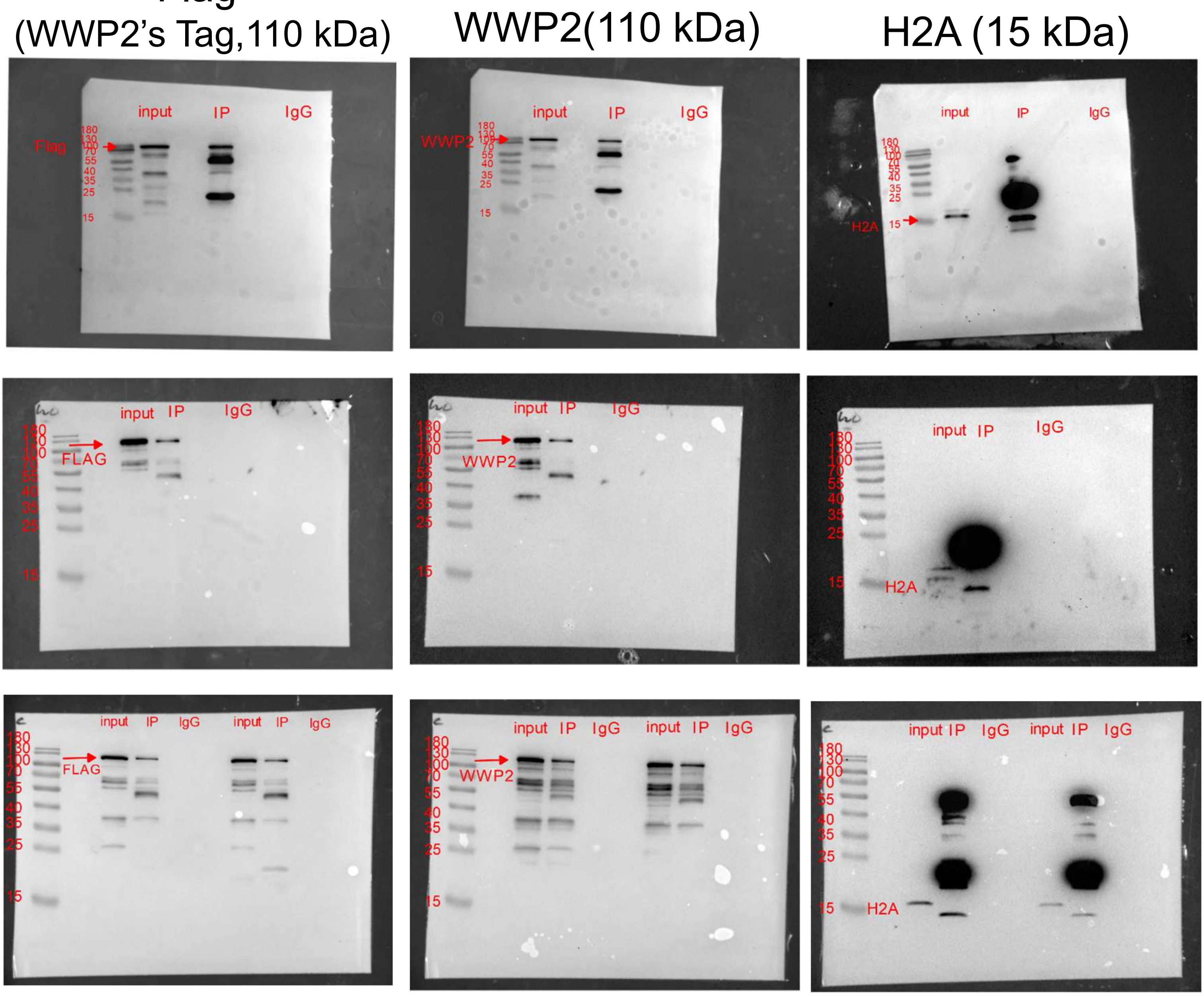

Figure 5E in vitro ubiquitylation assay

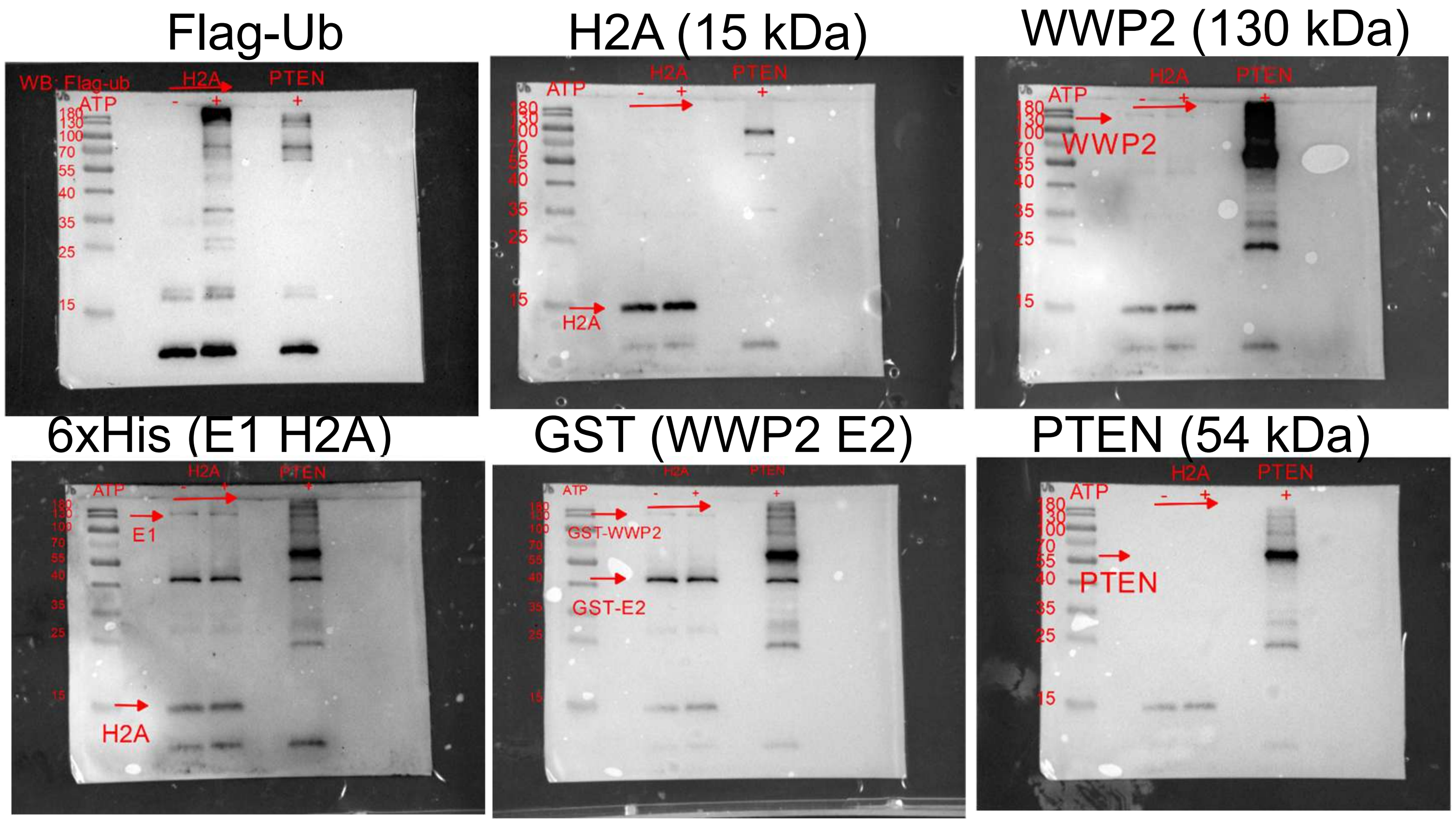

Figure 5E&F in vitro ubiquitylation assay

Flag-Ub-H2A-6xHis
(25 kDa)

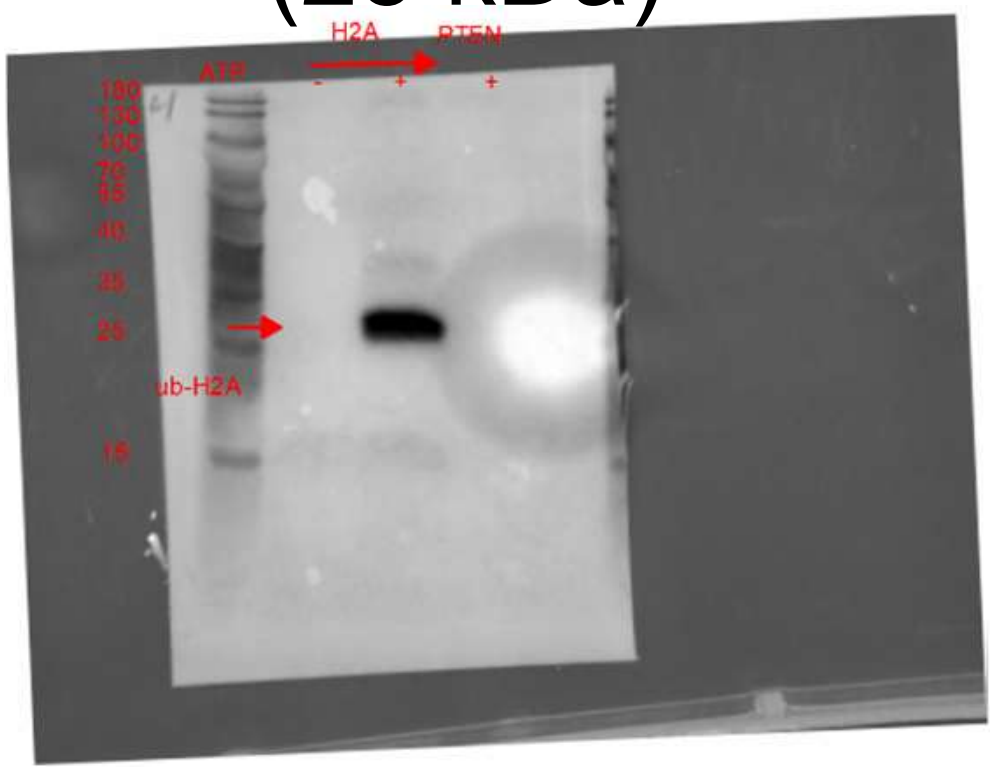

Flag-Ub

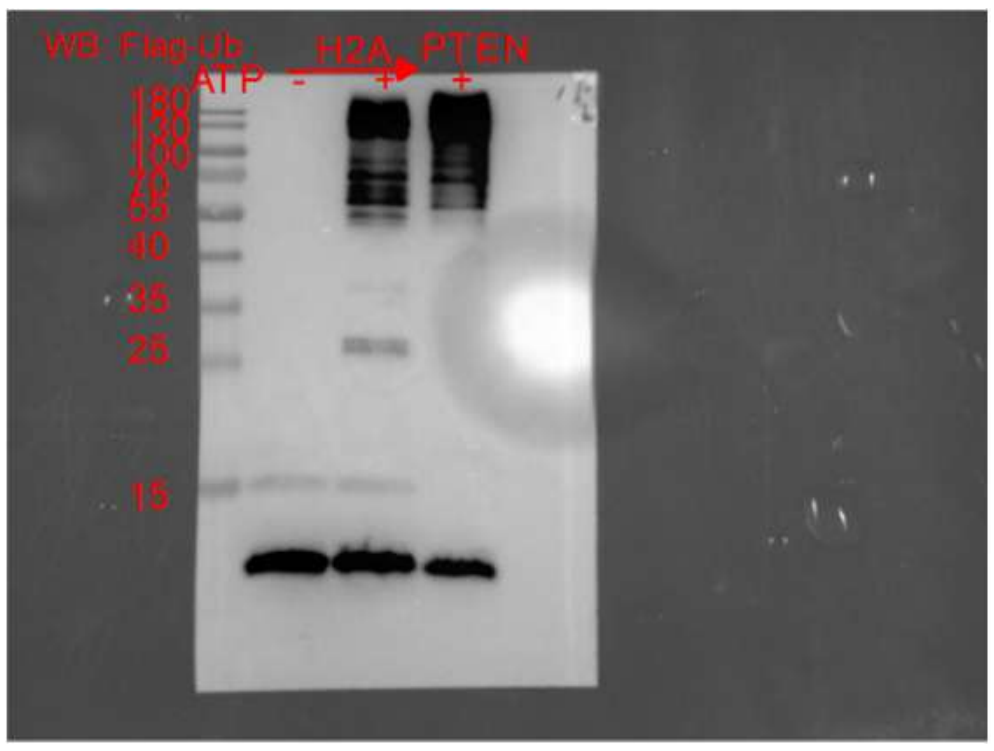

PTEN (54 kDa)

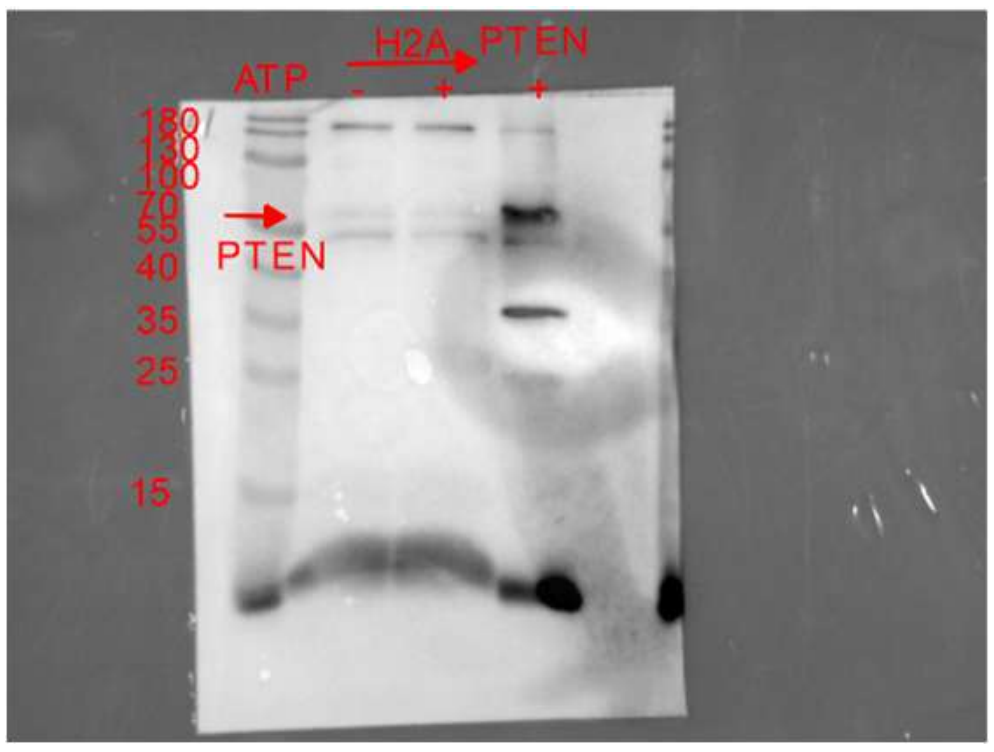

6xHis
(E1 121 kDa, H2A 15 kDa)

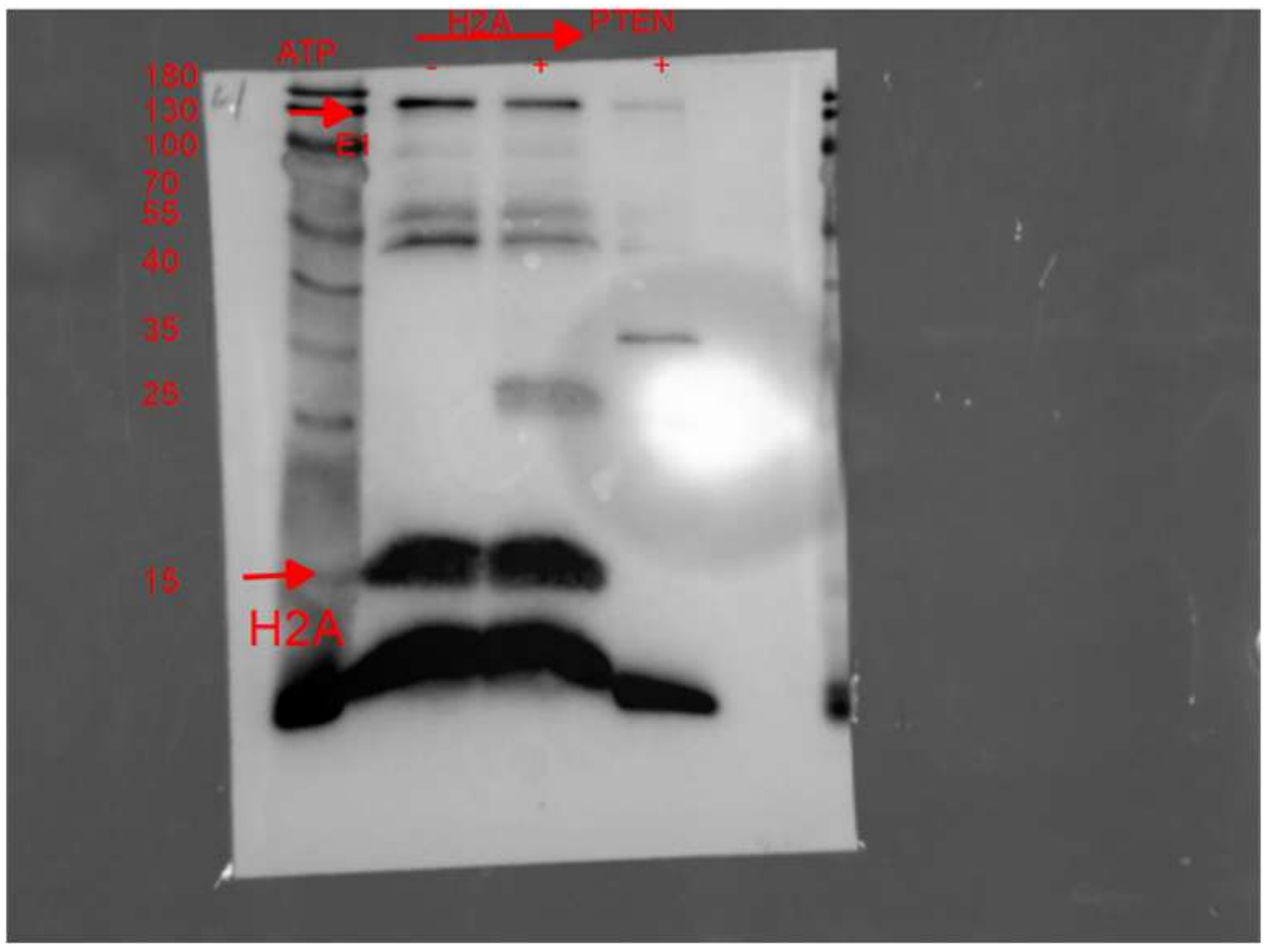

GST
(WWP2 130 kDa, E2 43 kDa)

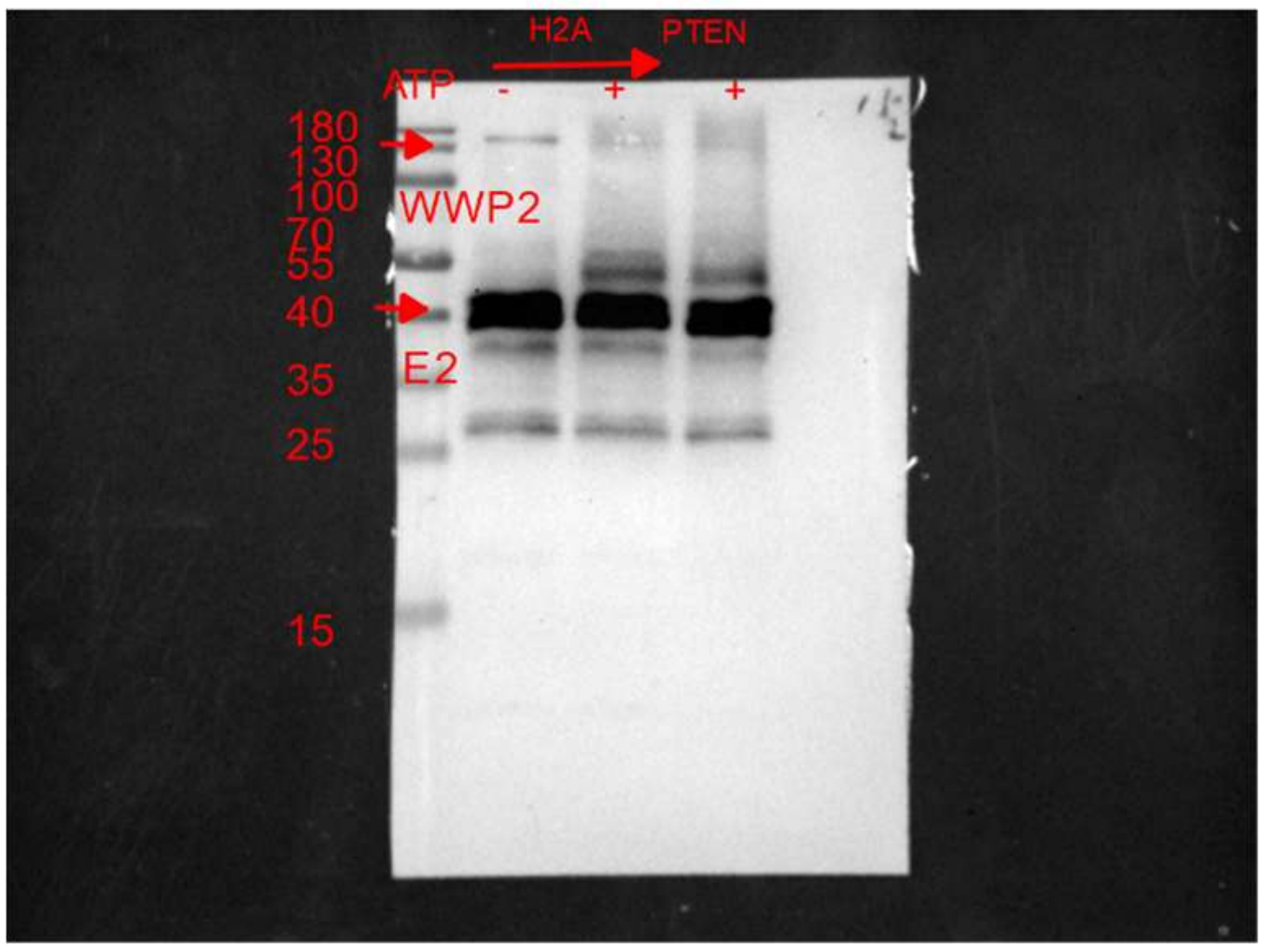

Flag-Ub-H2A-6xHis
(25 kDa)

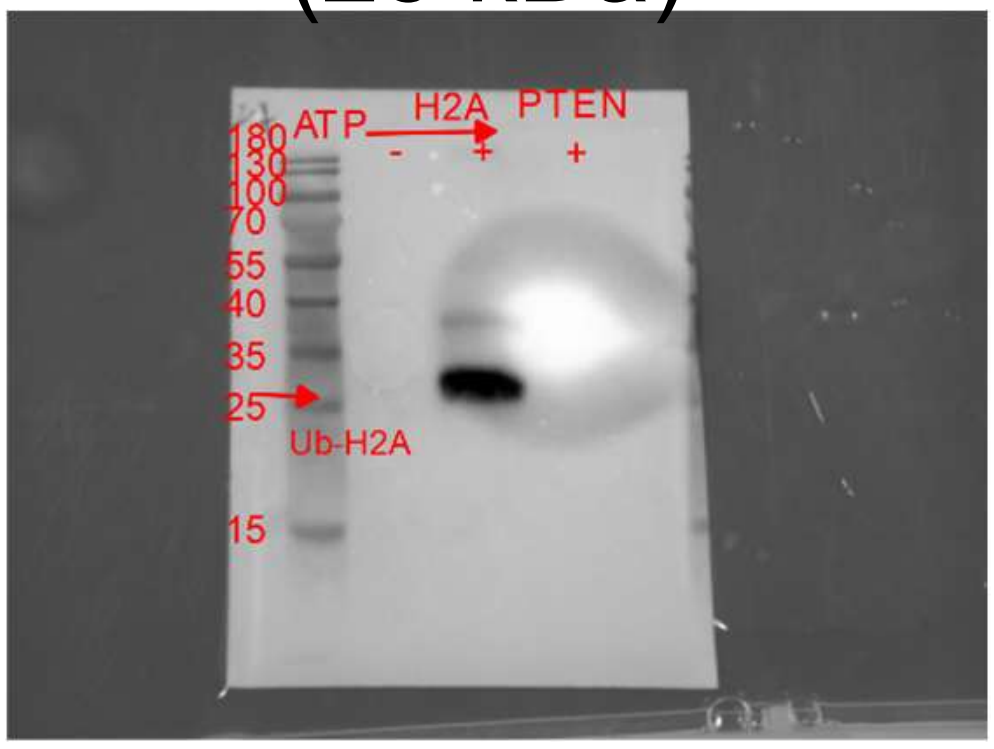

Flag-Ub

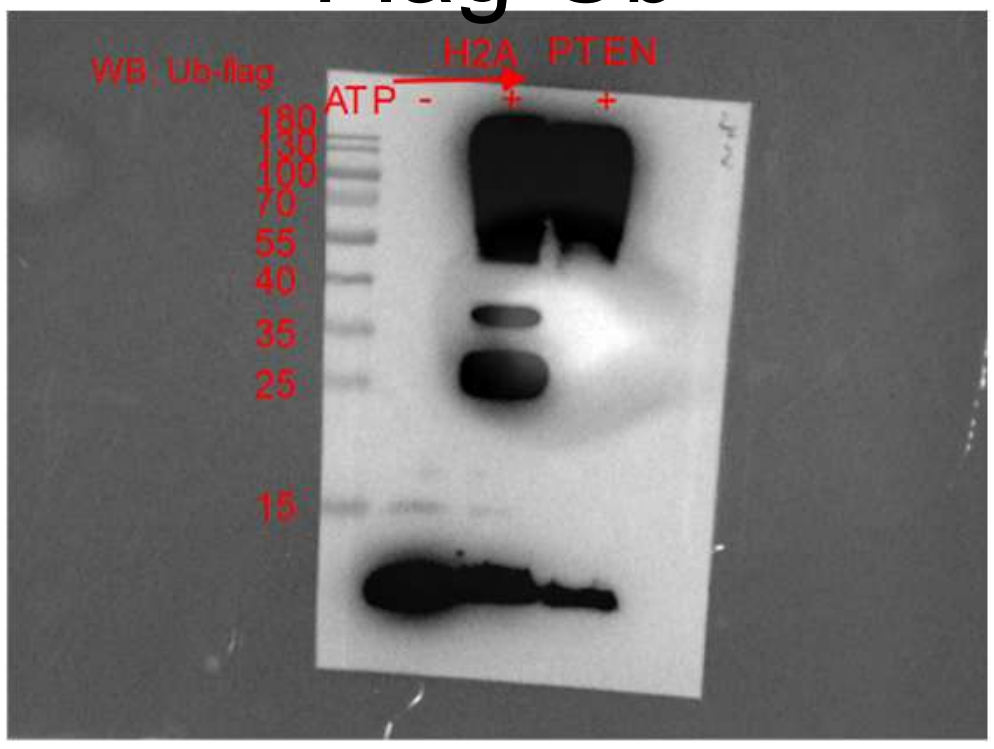

PTEN (54 kDa)

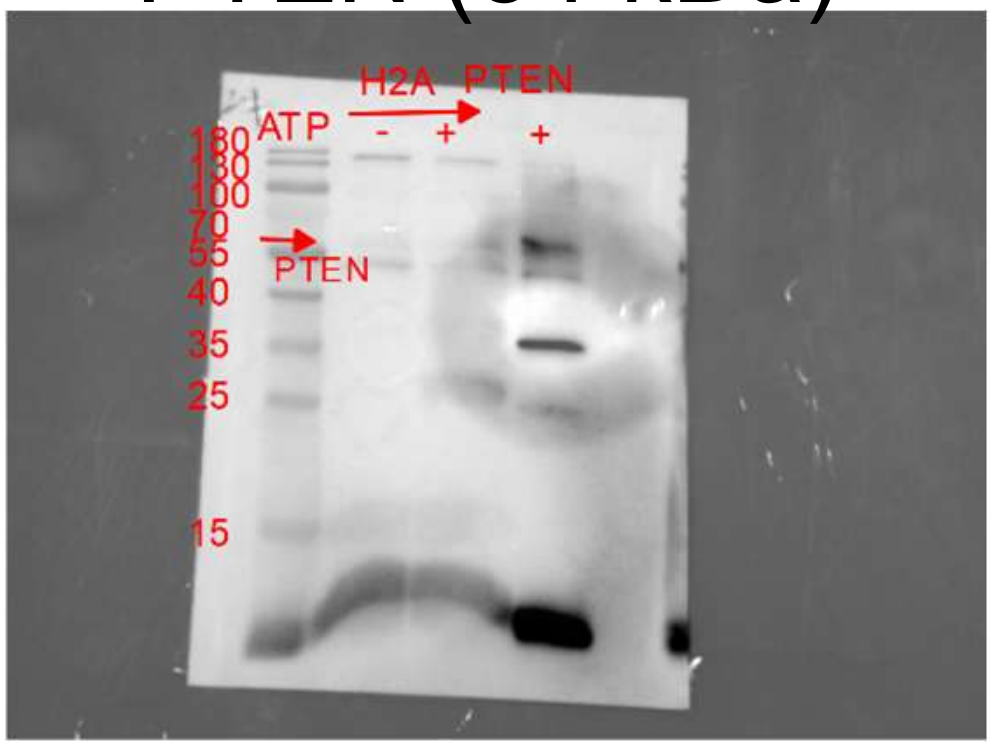

6xHis
(E1 121 kDa, H2A 15 kDa)

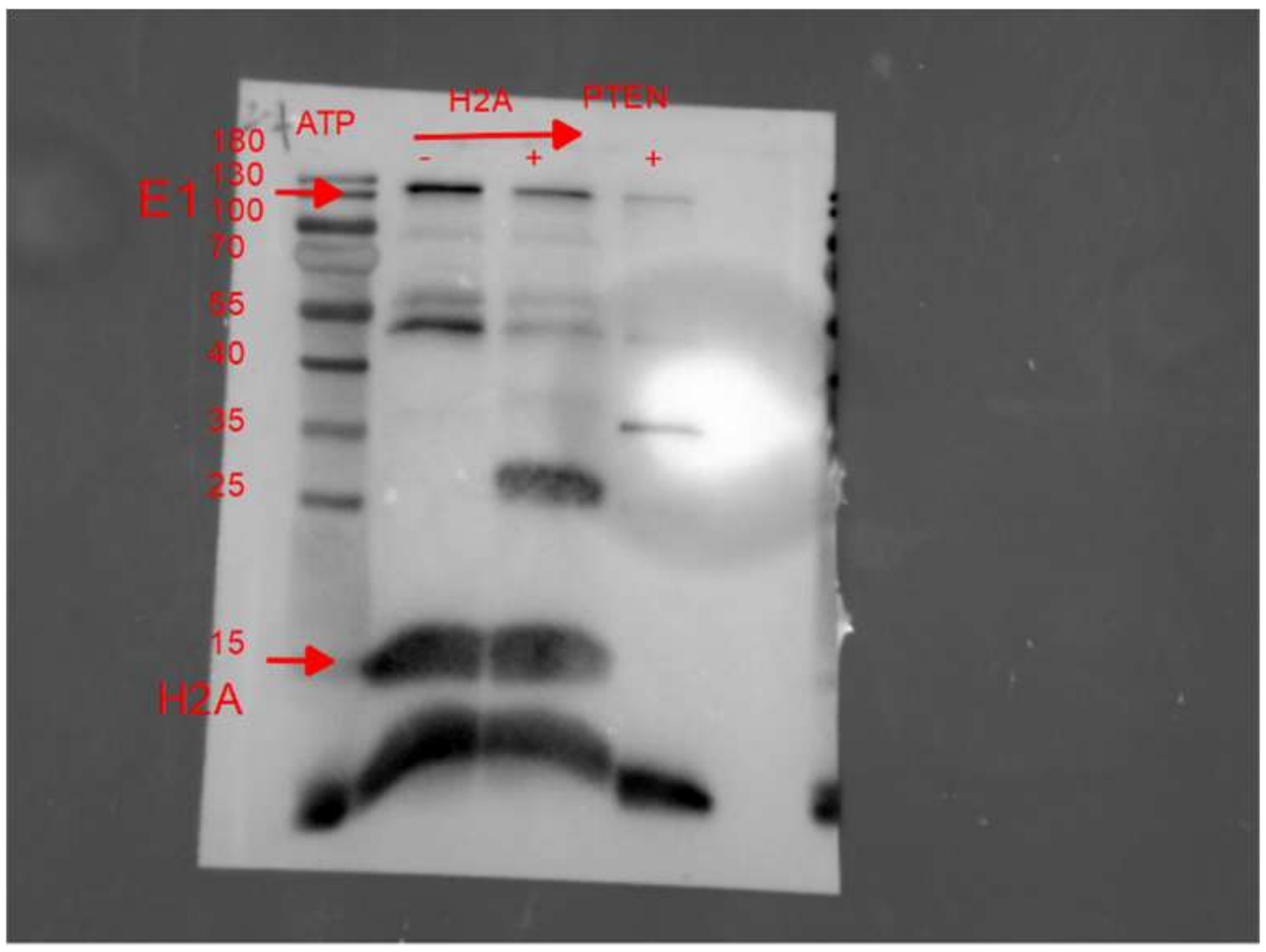

GST
(WWP2 130 kDa, E2 43 kDa)

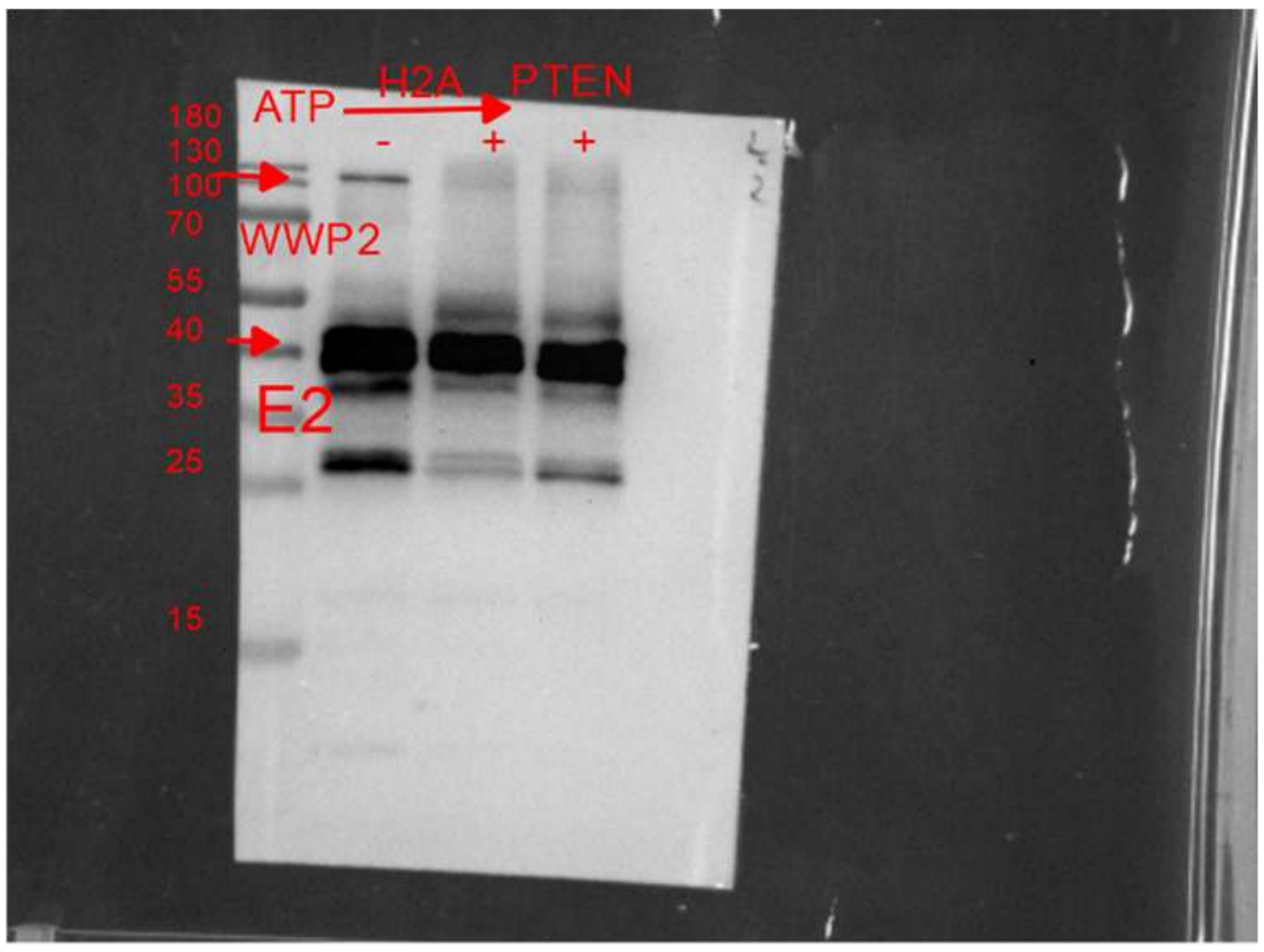

Flag-Ub-H2A-6xHis
(25 kDa)

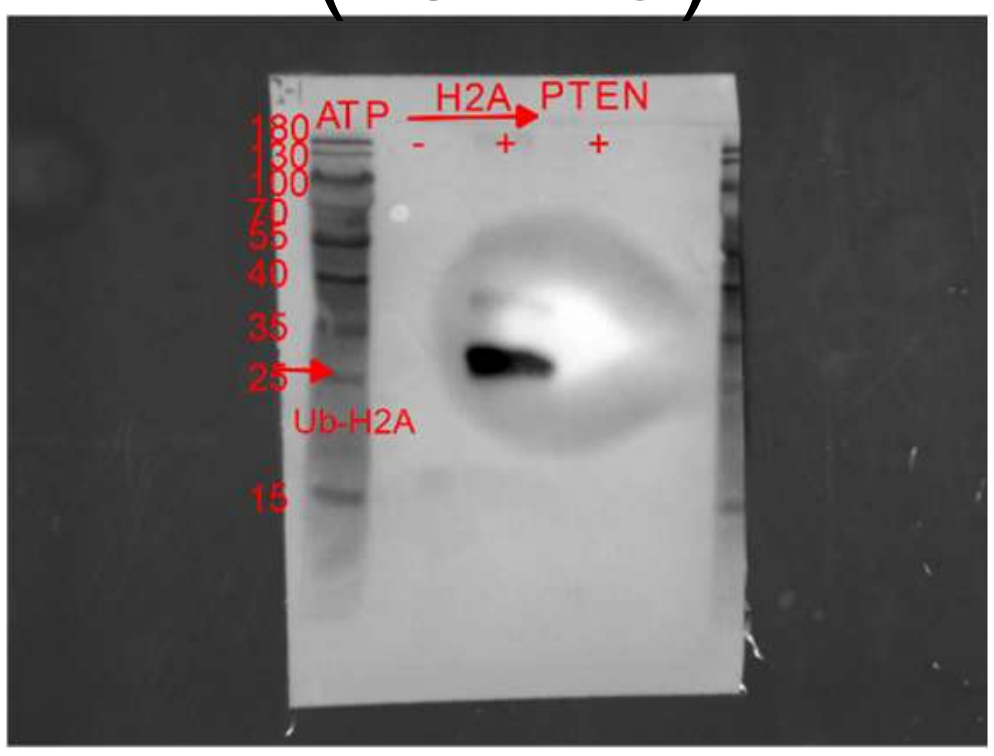

Flag-Ub

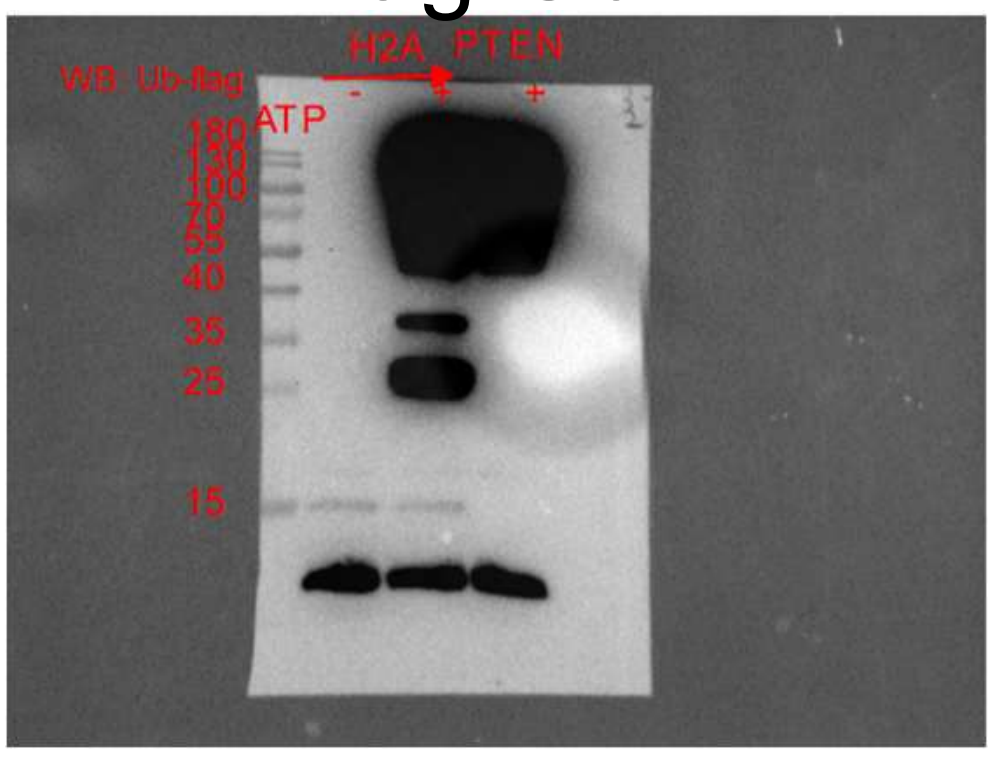

PTEN (54 kDa)

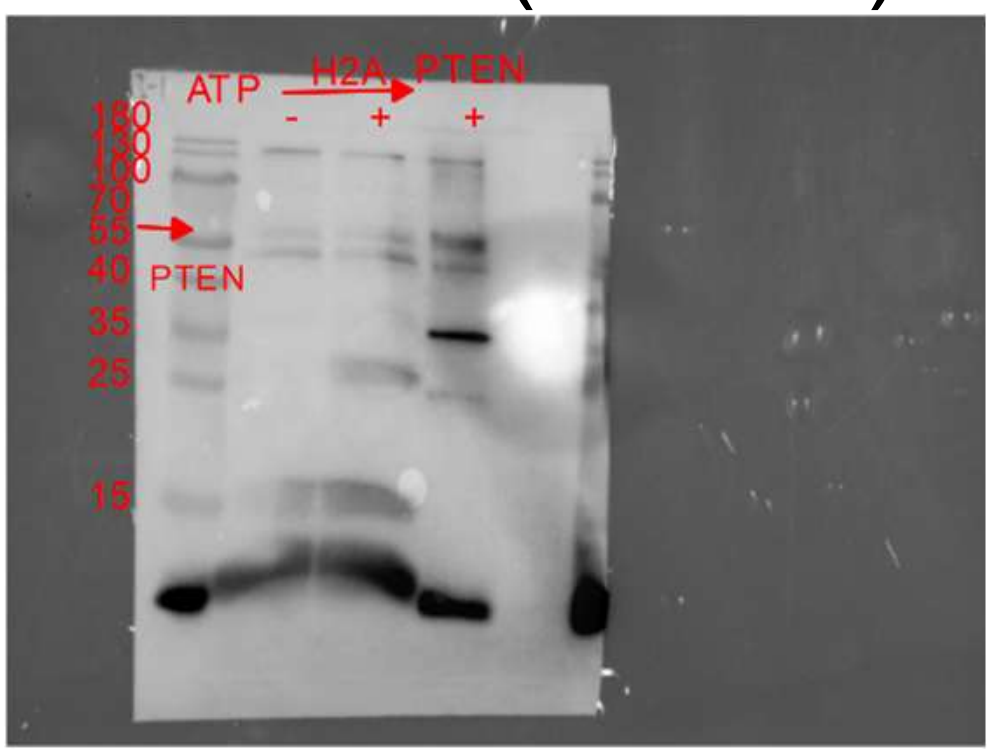

6xHis
(E1 121 kDa, H2A 15 kDa)

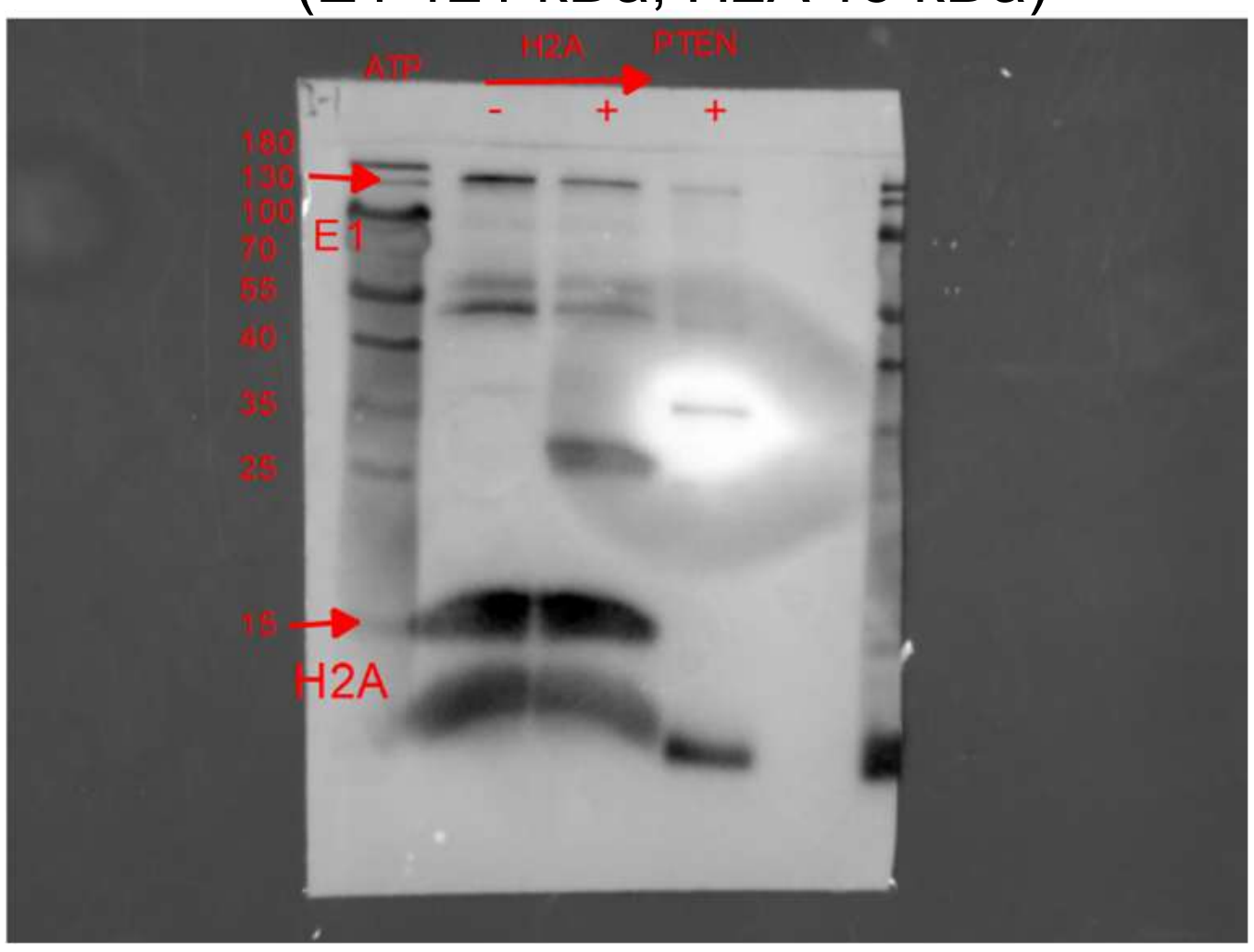

GST
(WWP2 130 kDa, E2 43 kDa)

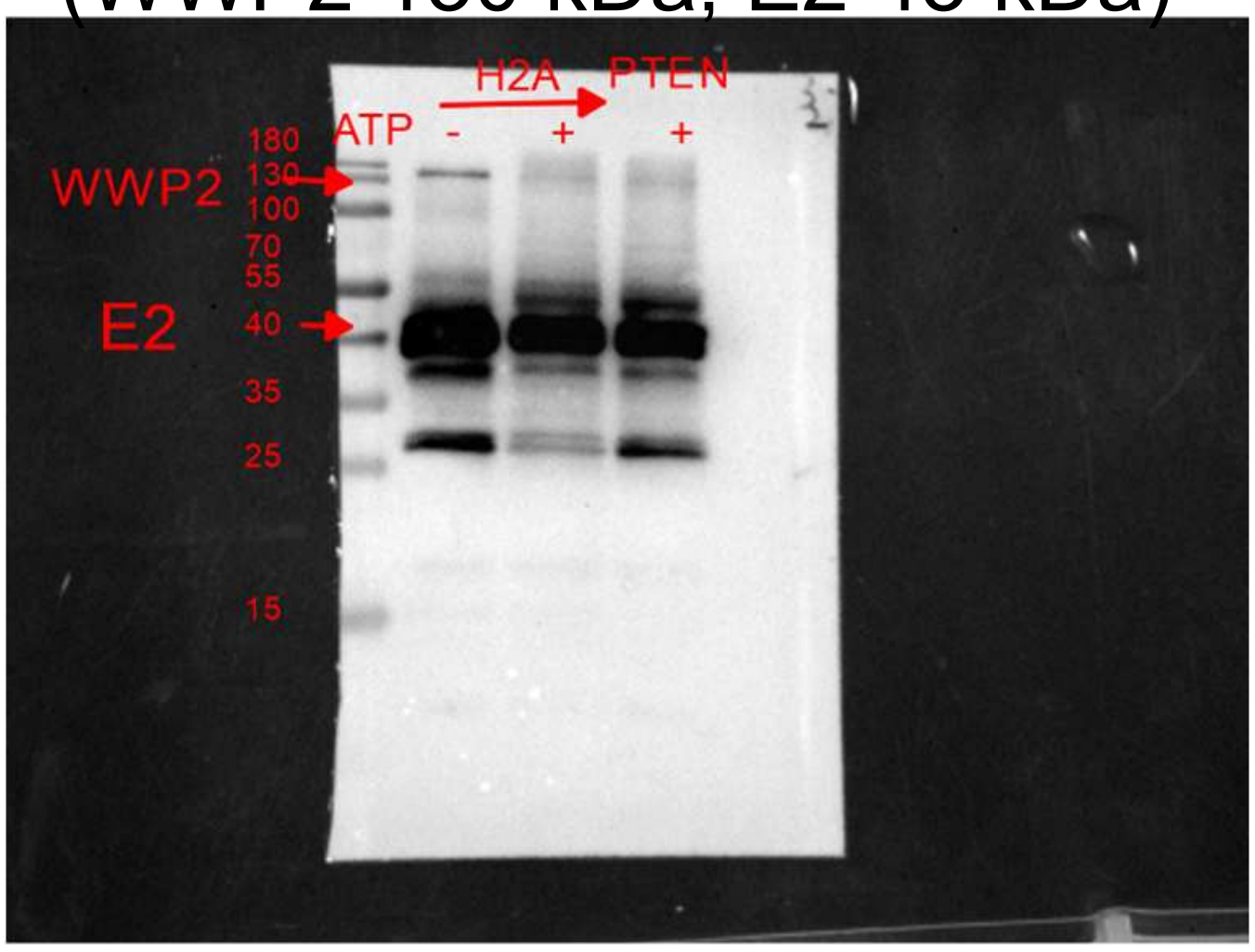

Figure 5H Ub-H2A (23 kDa) GAPDH (36 kDa)

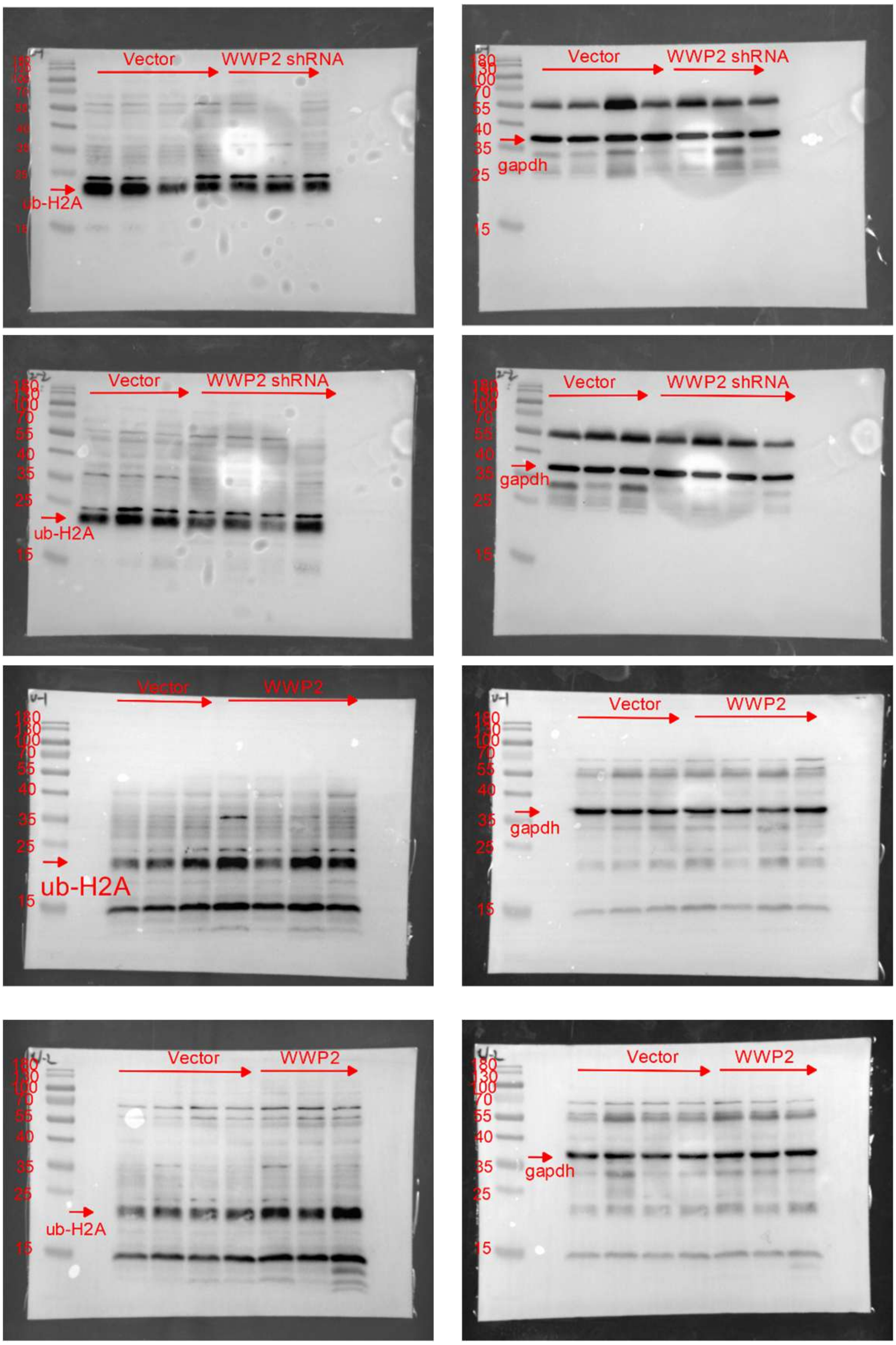

Figure 5K

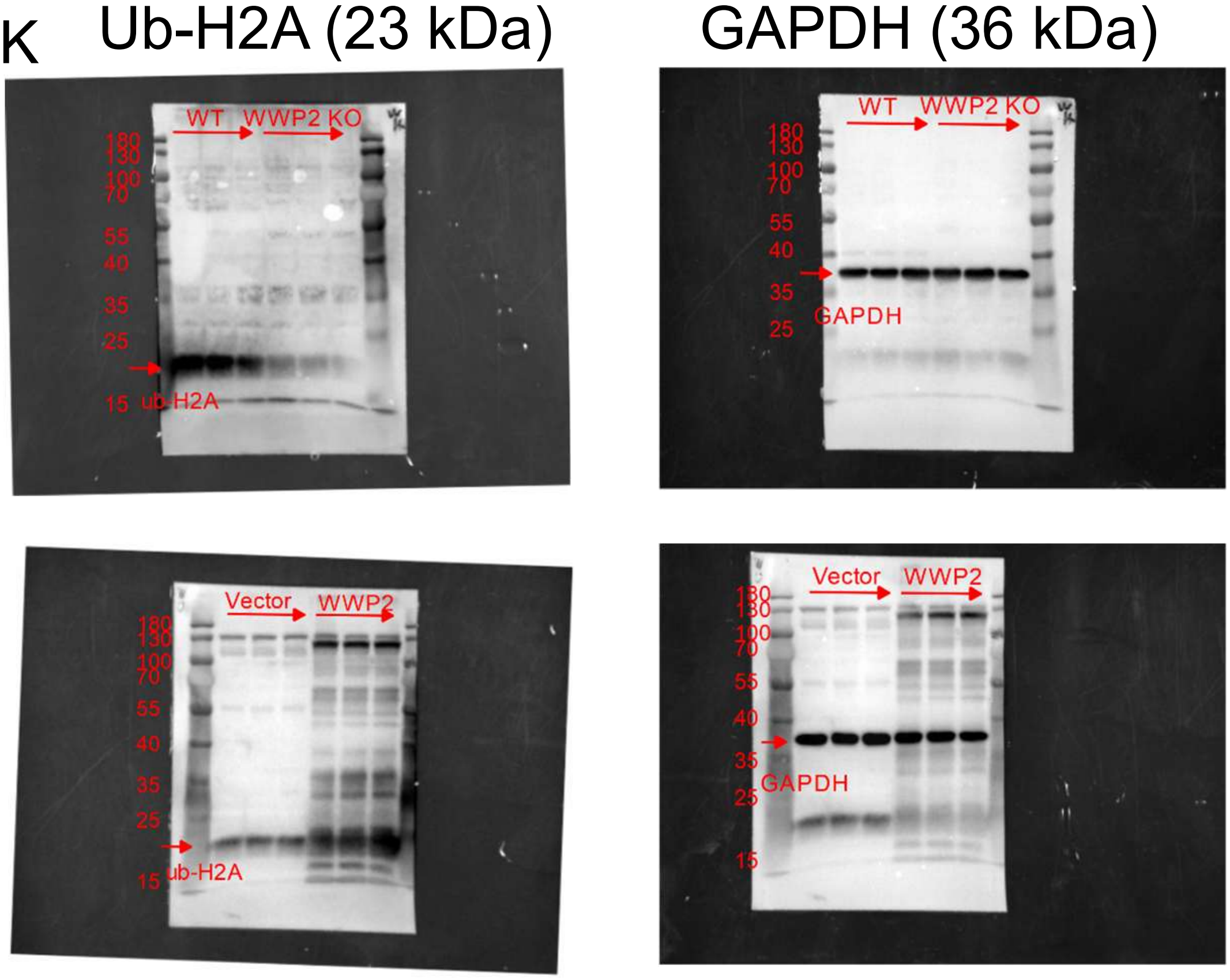
Ub-H2A (23 kDa)
GAPDH (36 kDa)
WT
WWP2 KO
180
130
100
70
55
40
35
25
15
ub-H2A
GAPDH
Vector
WWP2

Figure 6F

Ub-H2A (23 kDa)

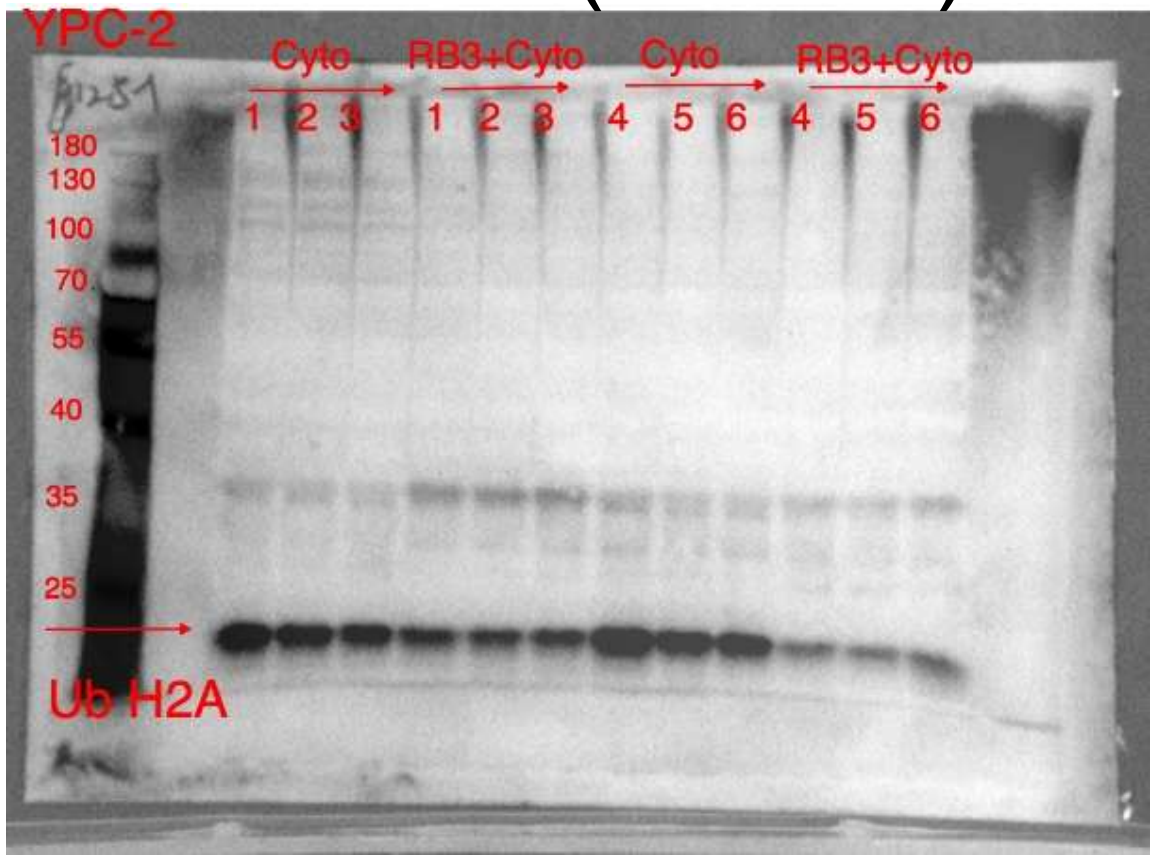

β-actin (42 kDa)

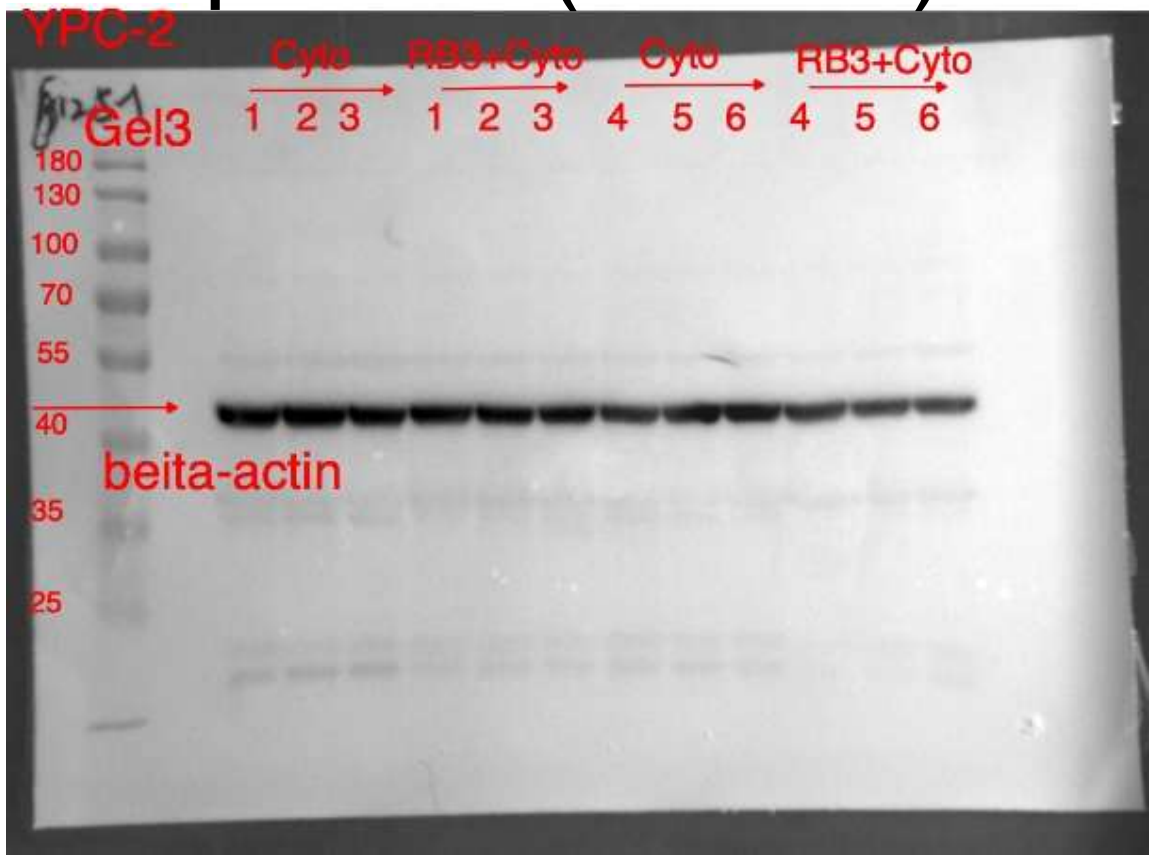

WT-1 (54 kDa)

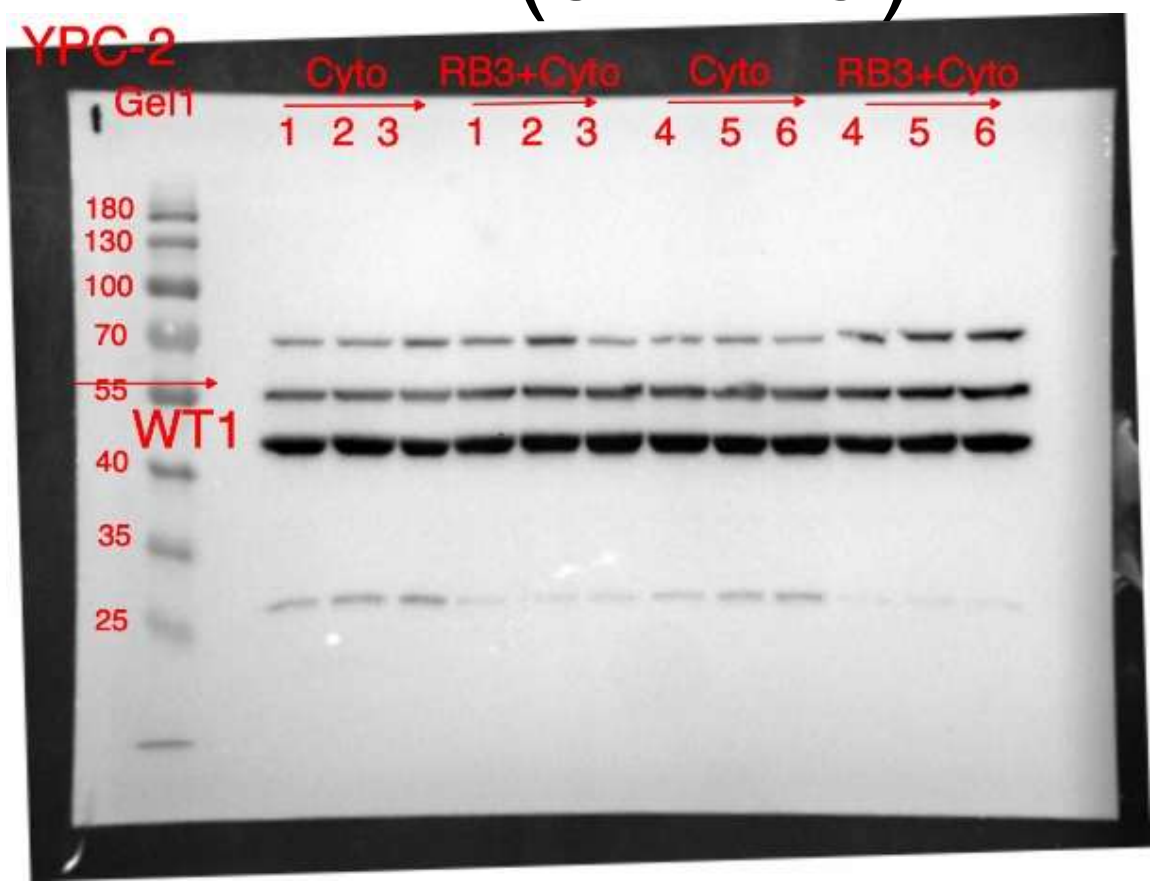

GAPDH (36 kDa)

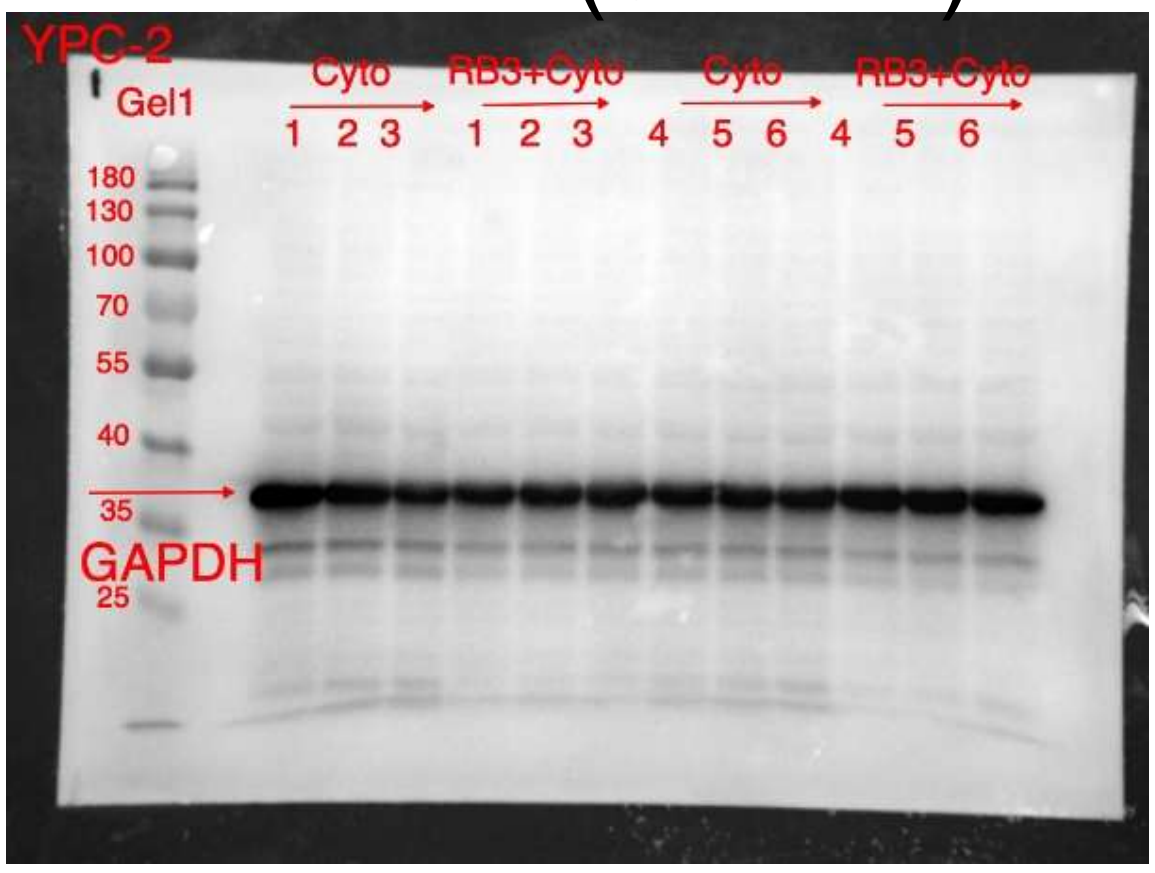

Figure 6I

Ub-H2A (23 kDa)

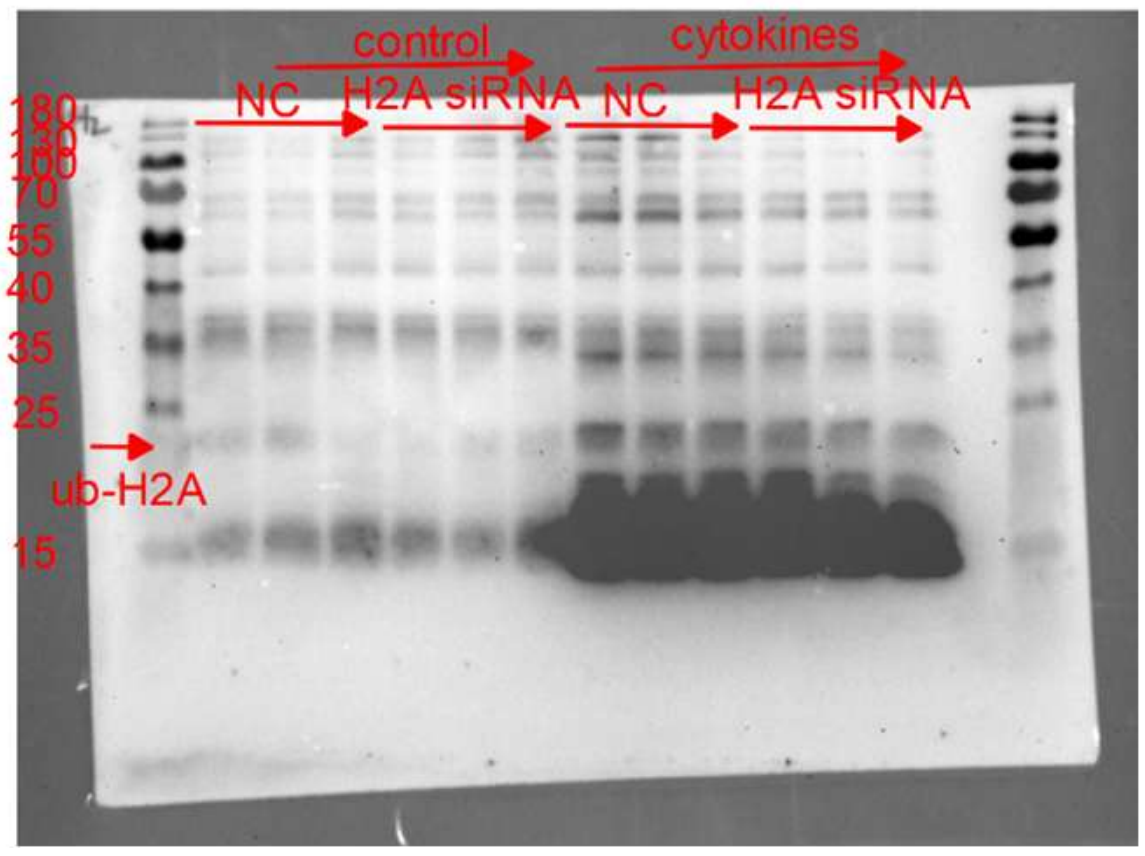

GAPDH (36 kDa)

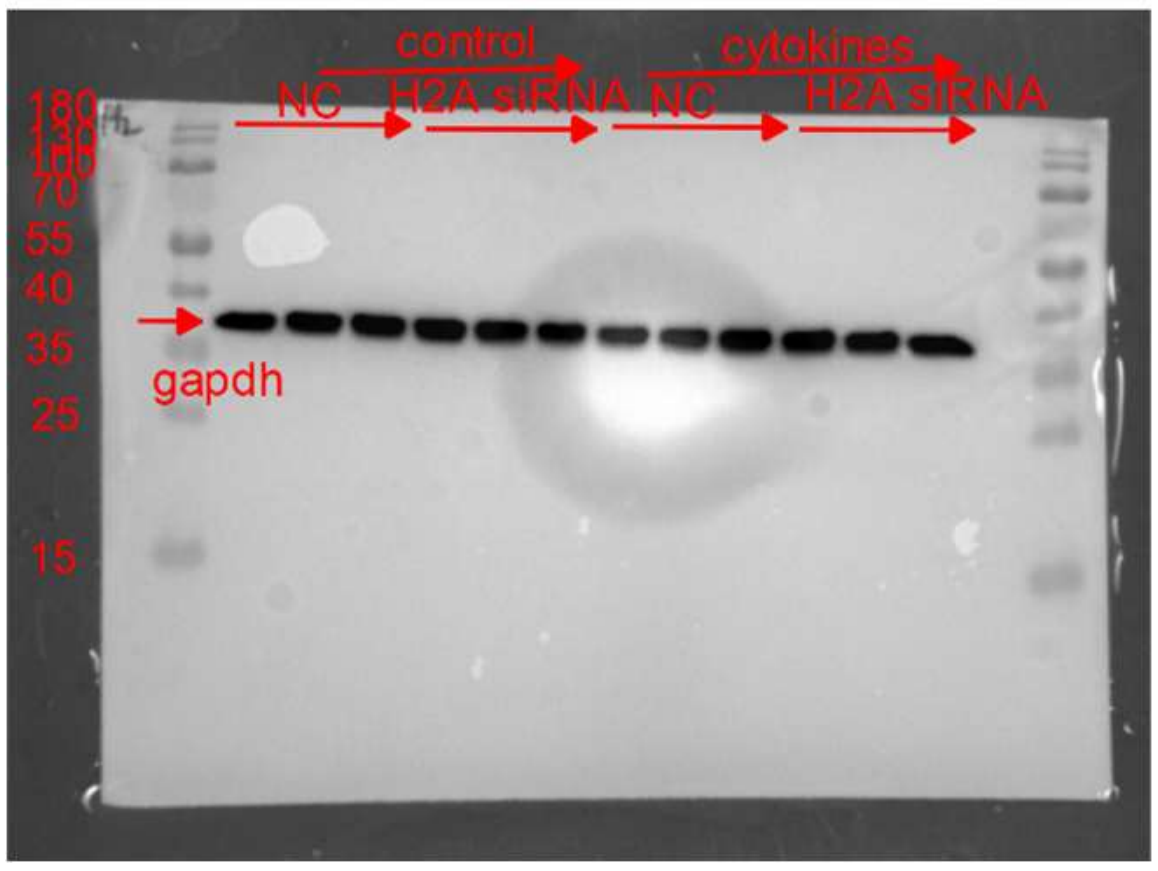

WT-1 (54 kDa)

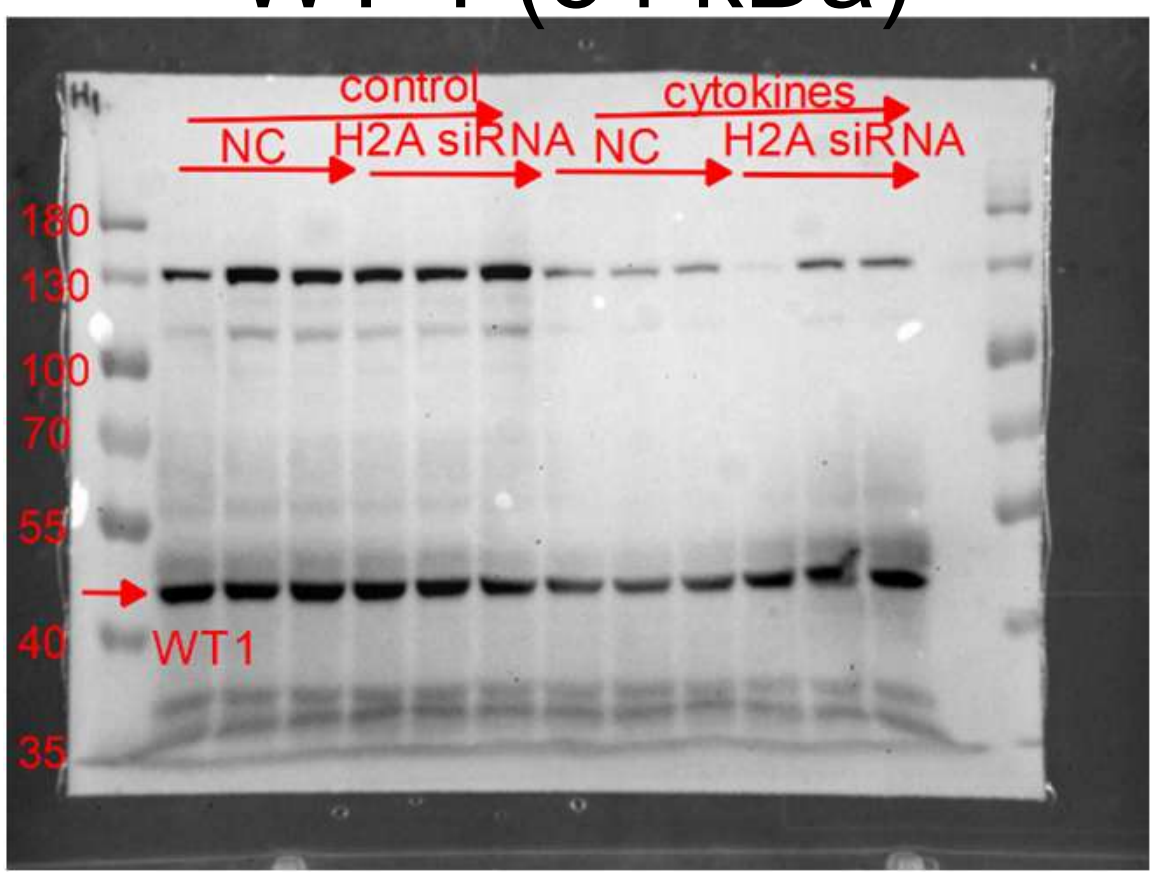

GAPDH (36 kDa)

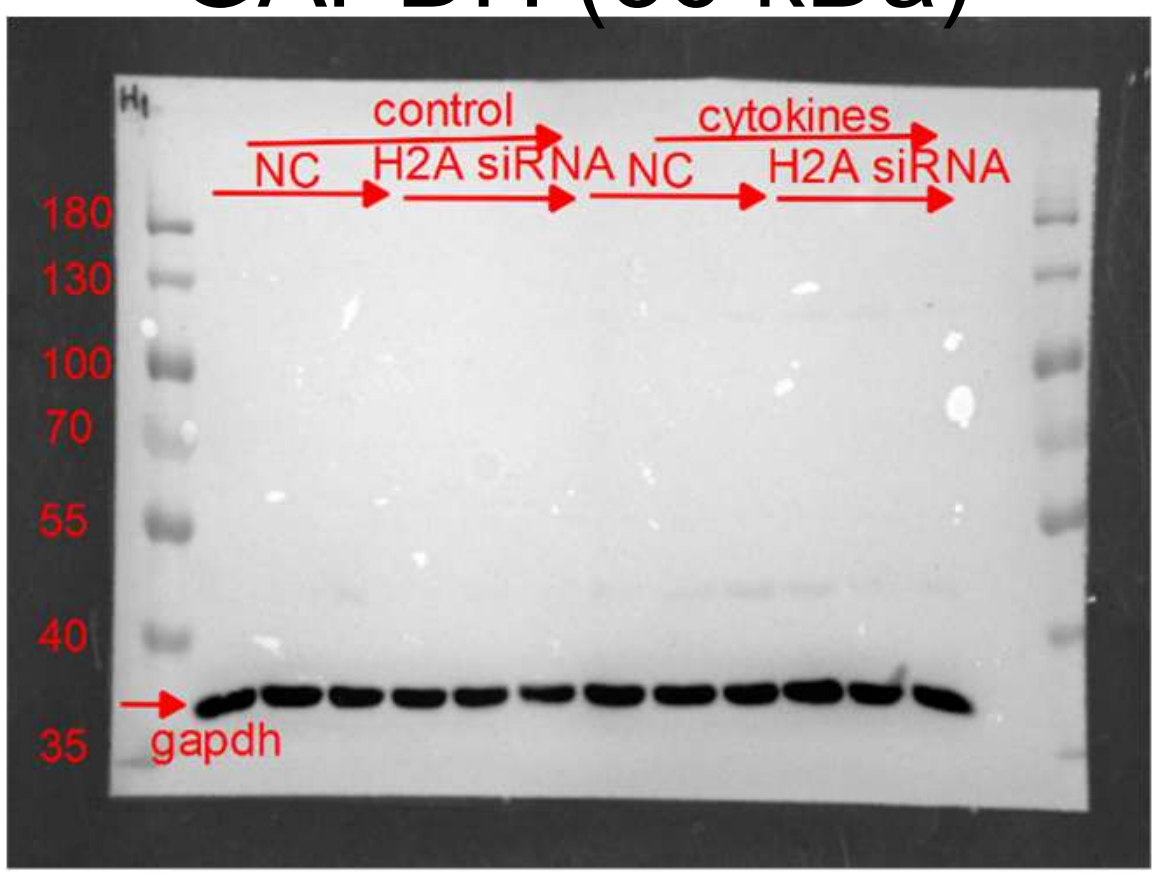

Figure 6L

WT-1 (54 kDa)

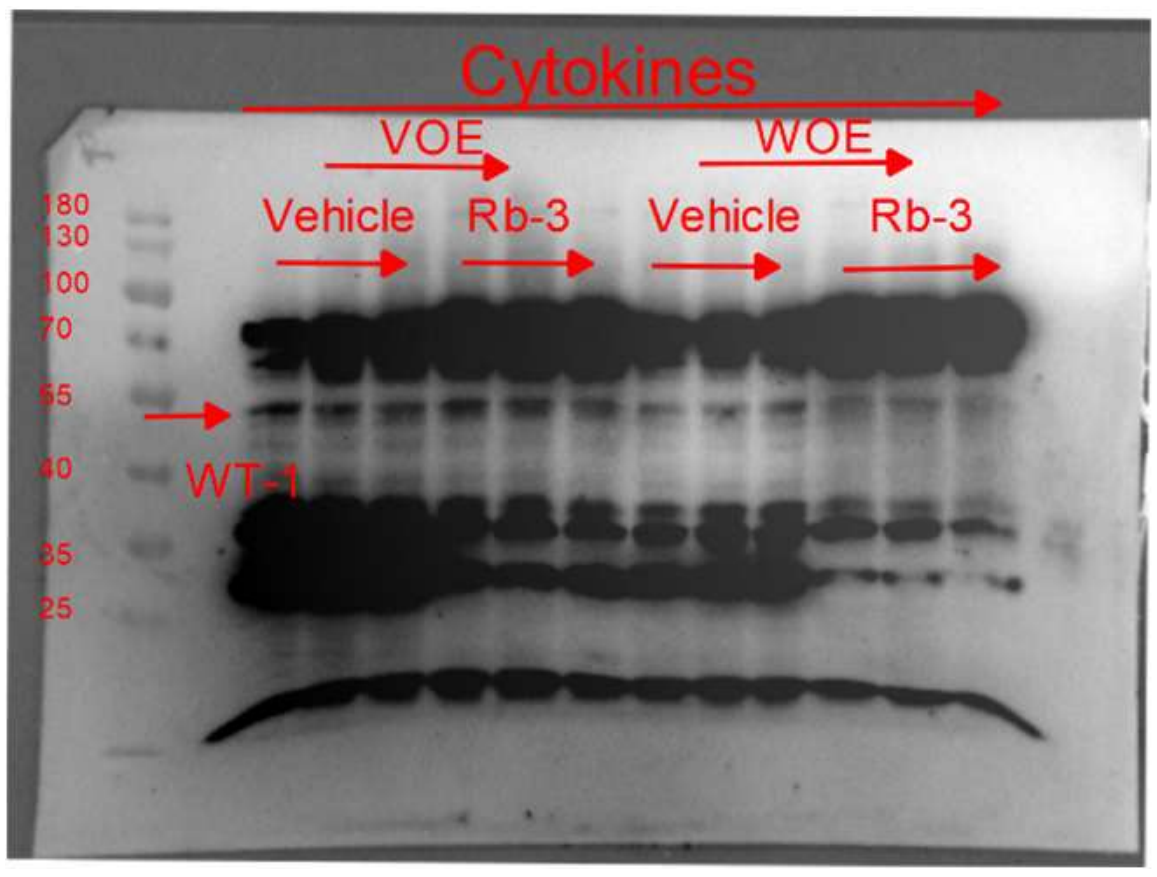

GAPDH (36 kDa)

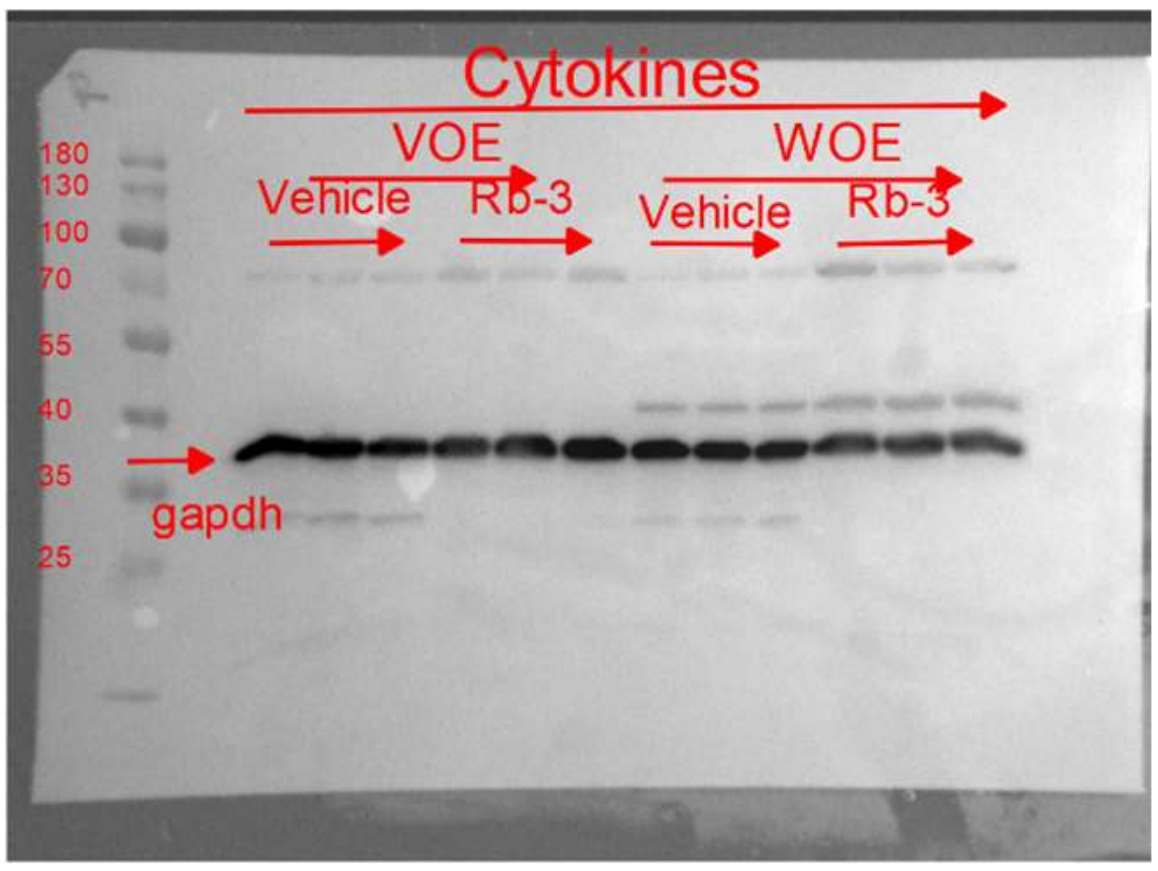

Nephrin (180 kDa)

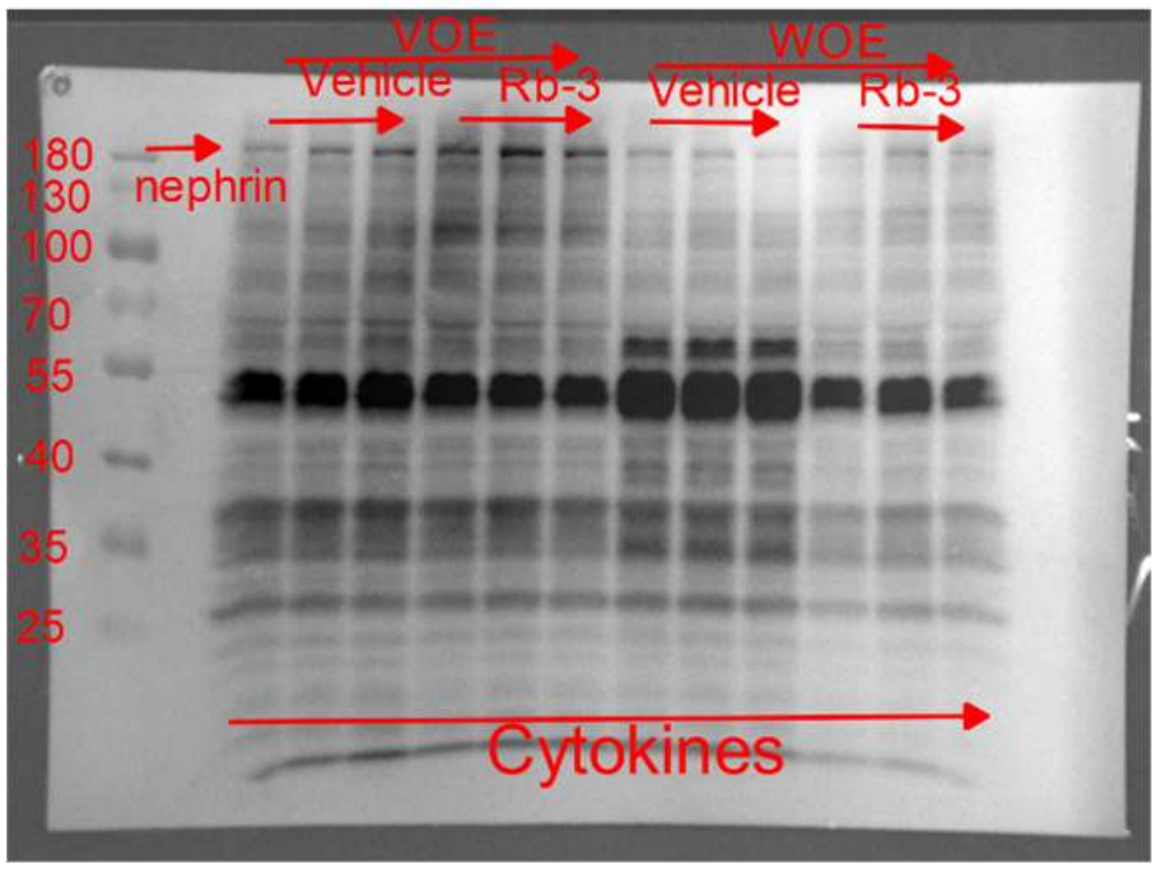

GAPDH (36 kDa)

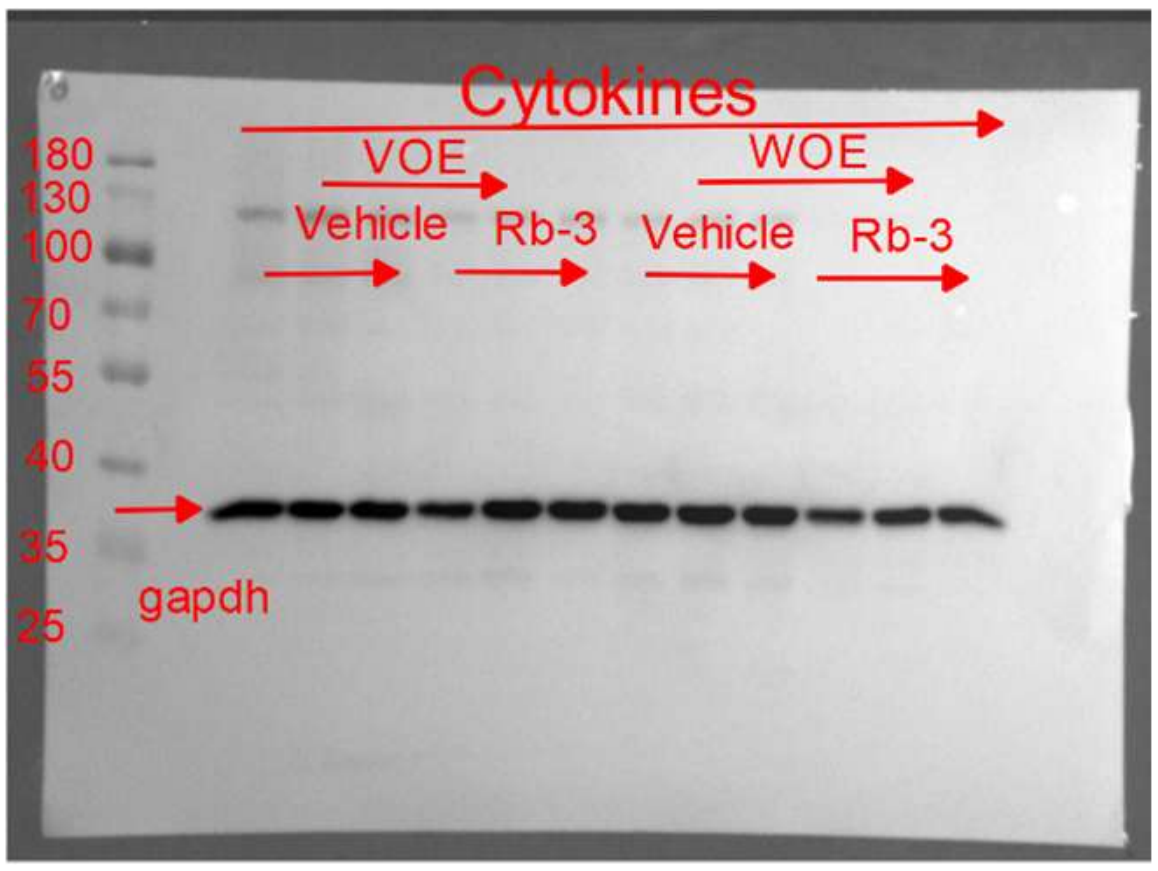

Figure 7I

FAK (110 kDa)

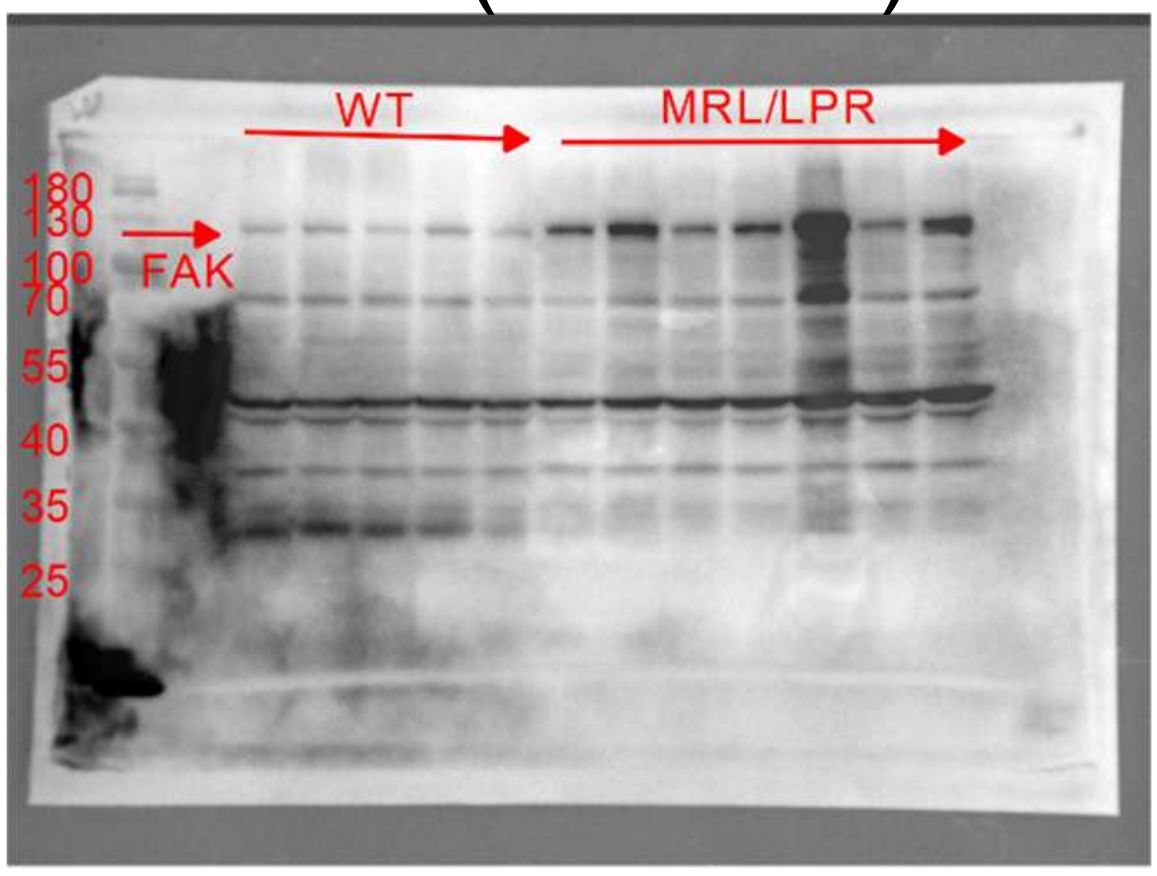

GAPDH (36 kDa)

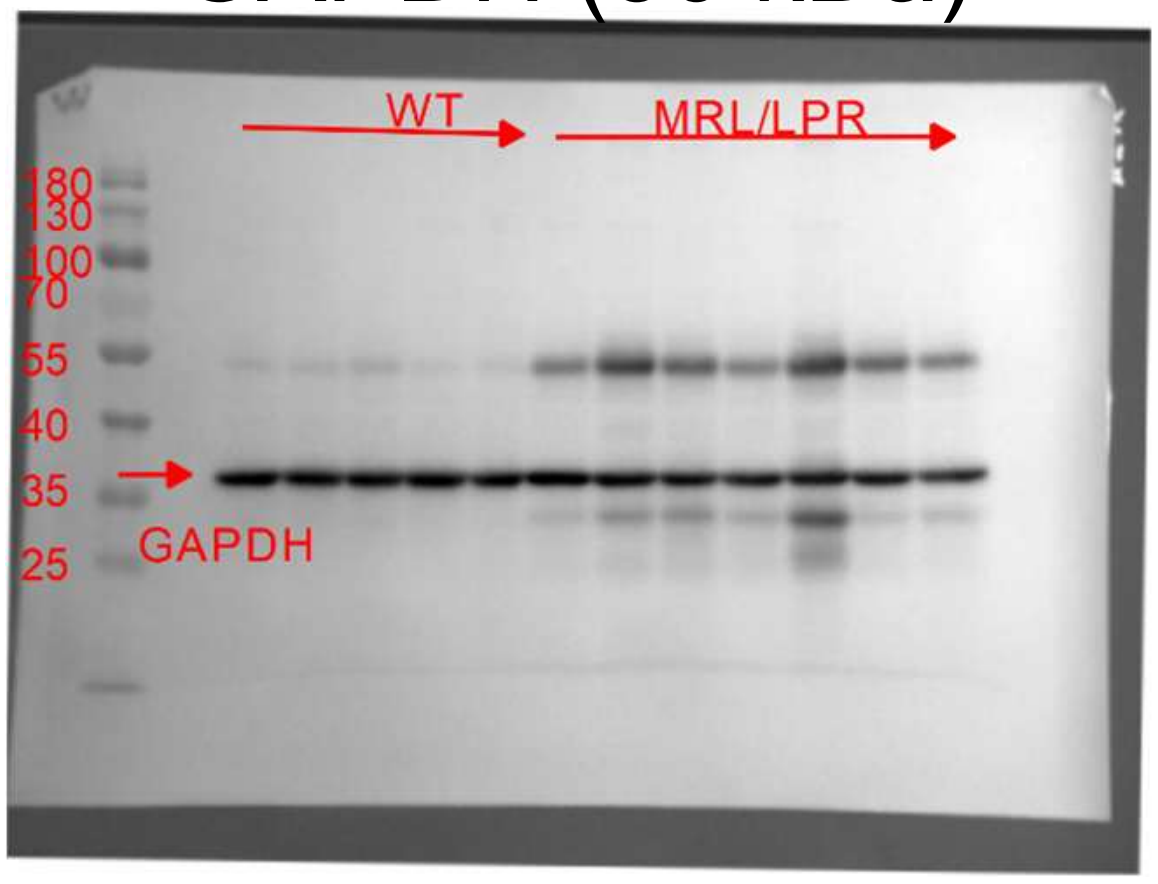

Figure 7K

FAK (110 kDa)

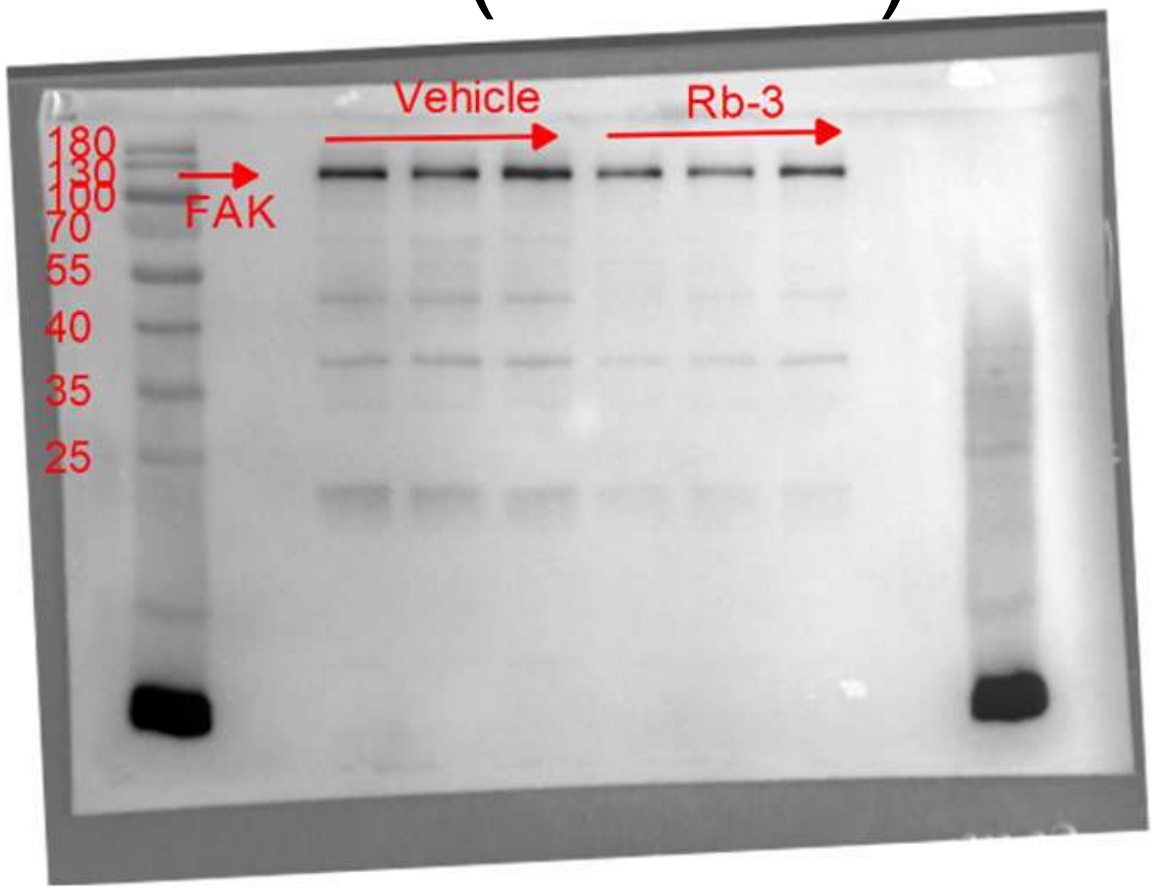

GAPDH (36 kDa)

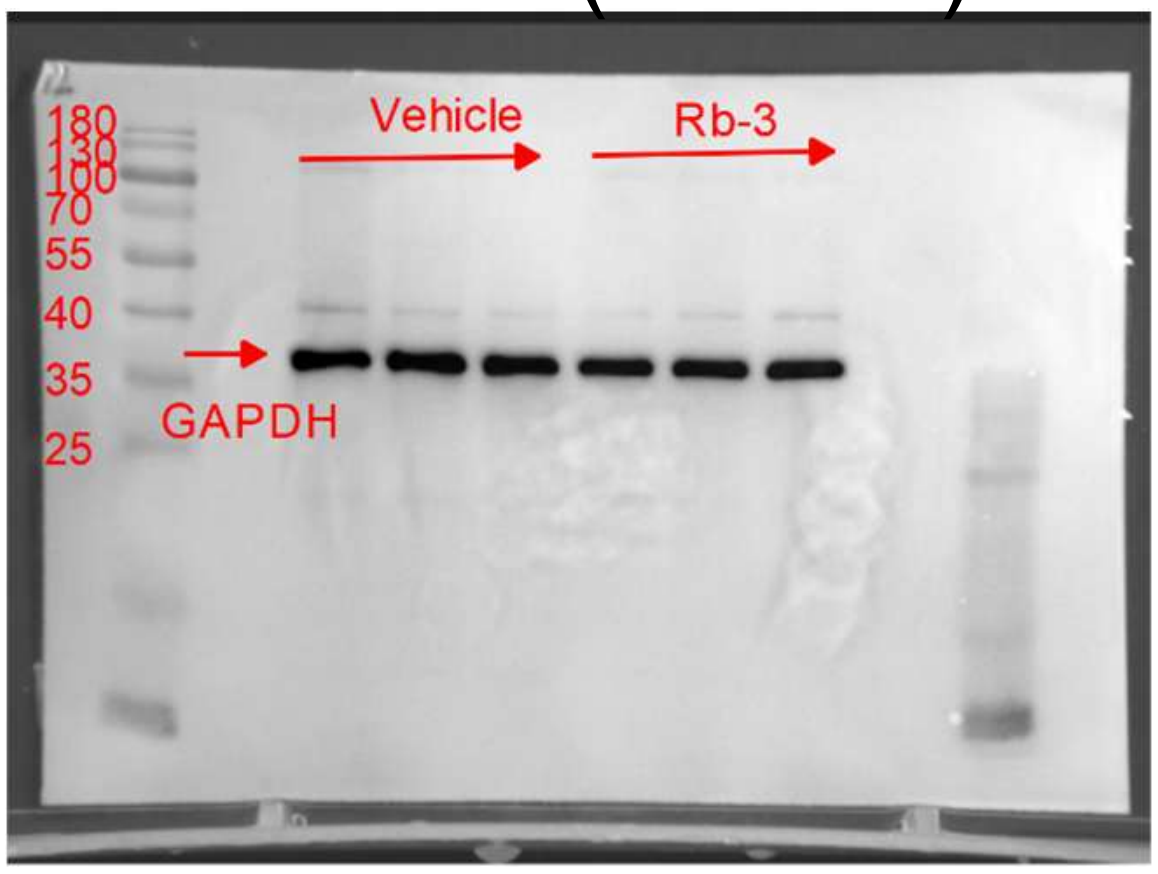

Figure 7M

p-FAK (125 kDa)

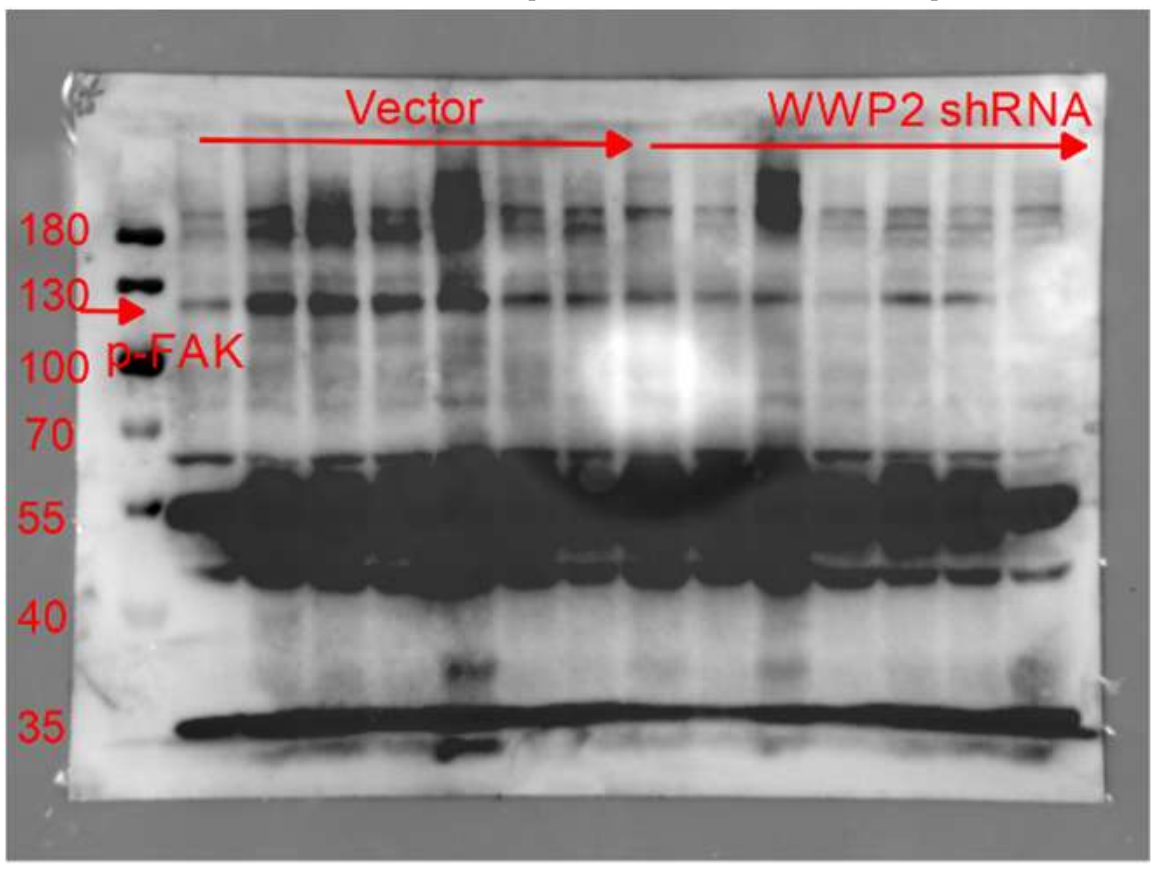

β-actin (42 kDa)

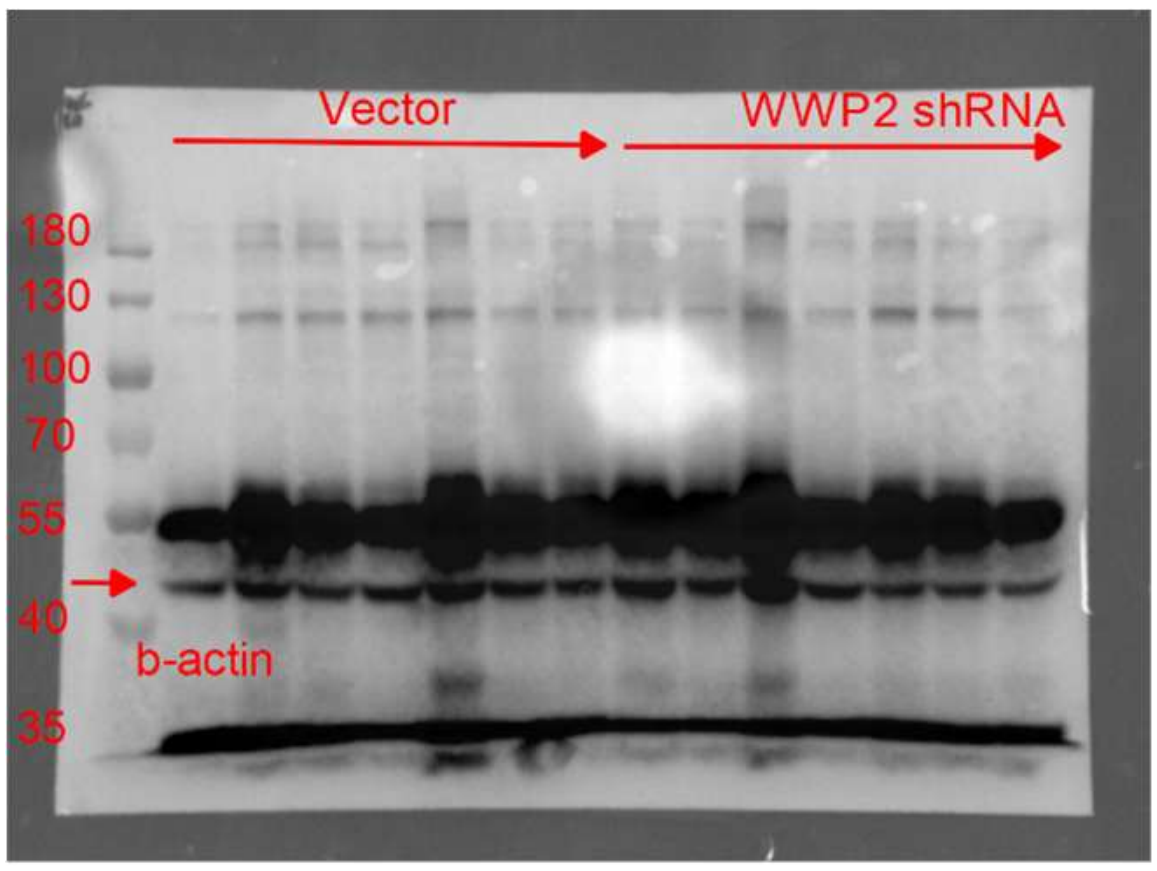

Figure 7O

WT-1 (54 kDa)

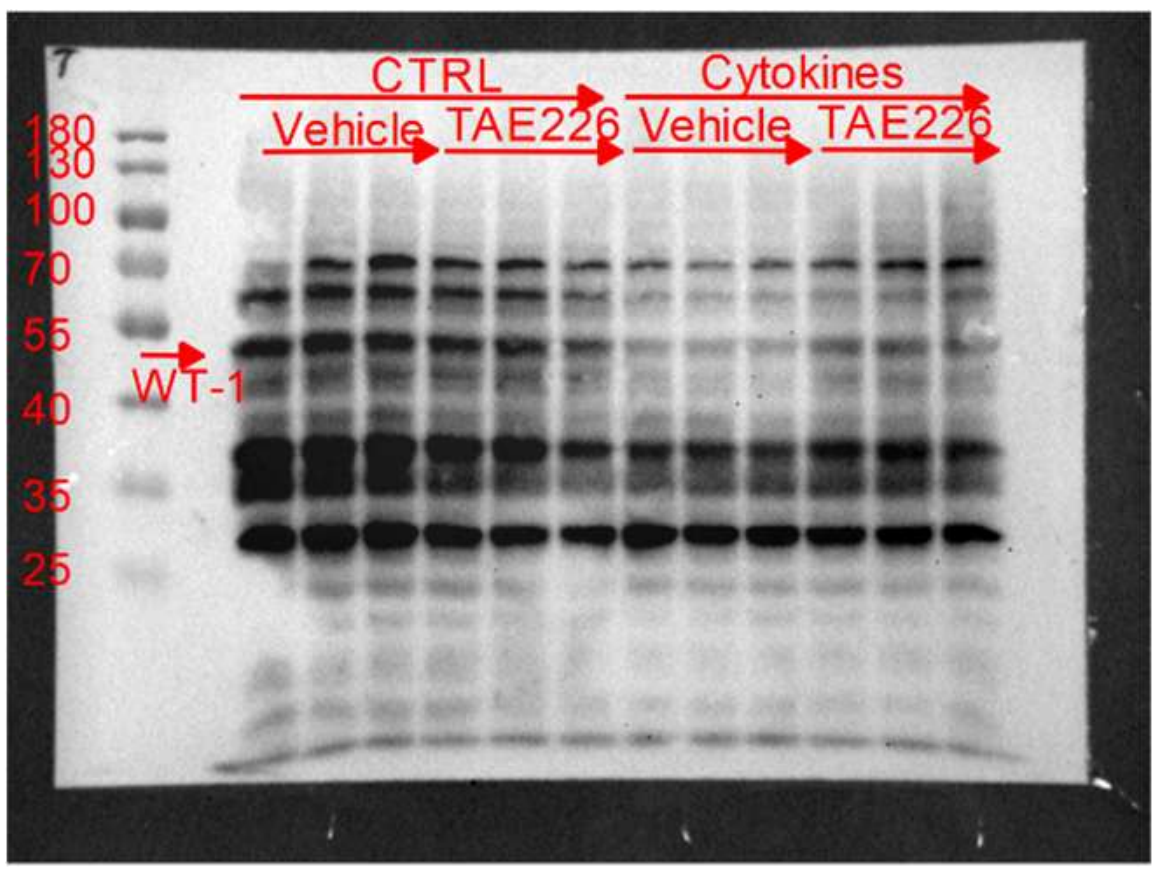

GAPDH (36 kDa)

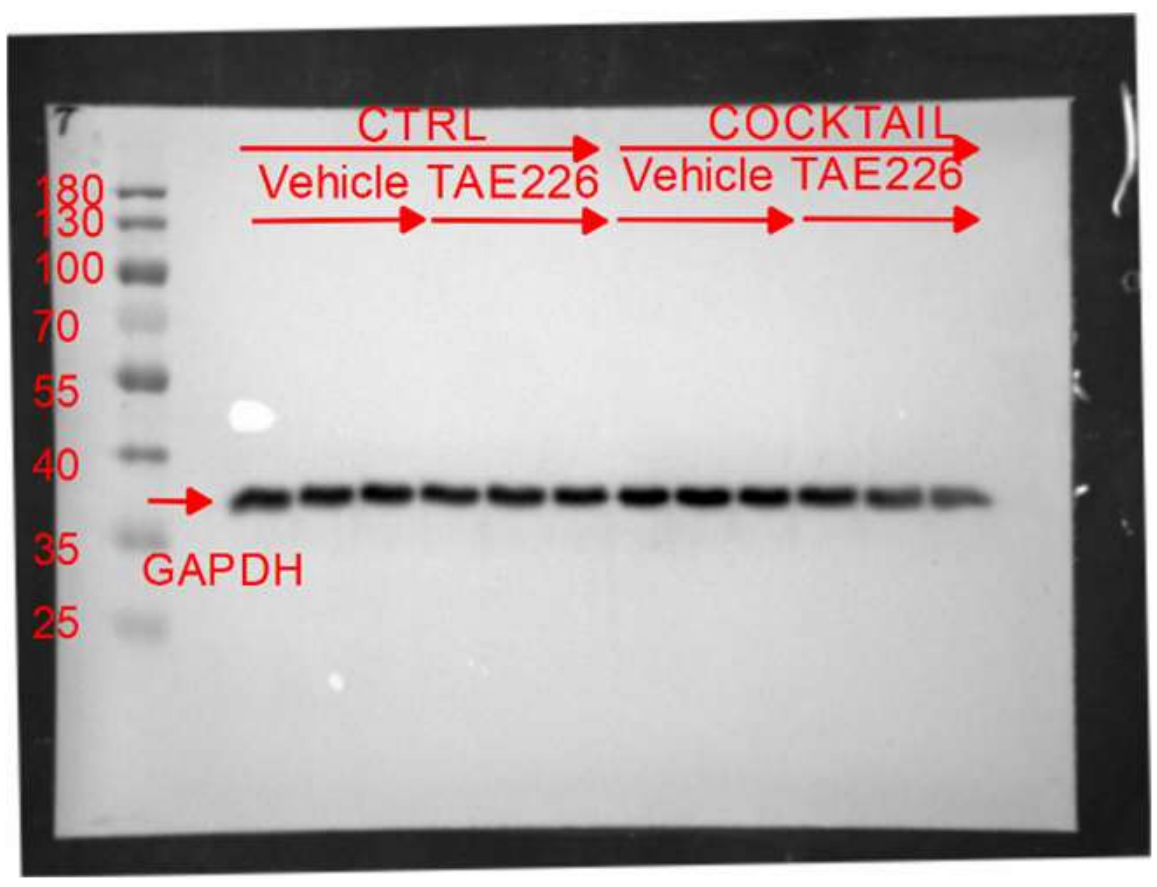

Figure S1B

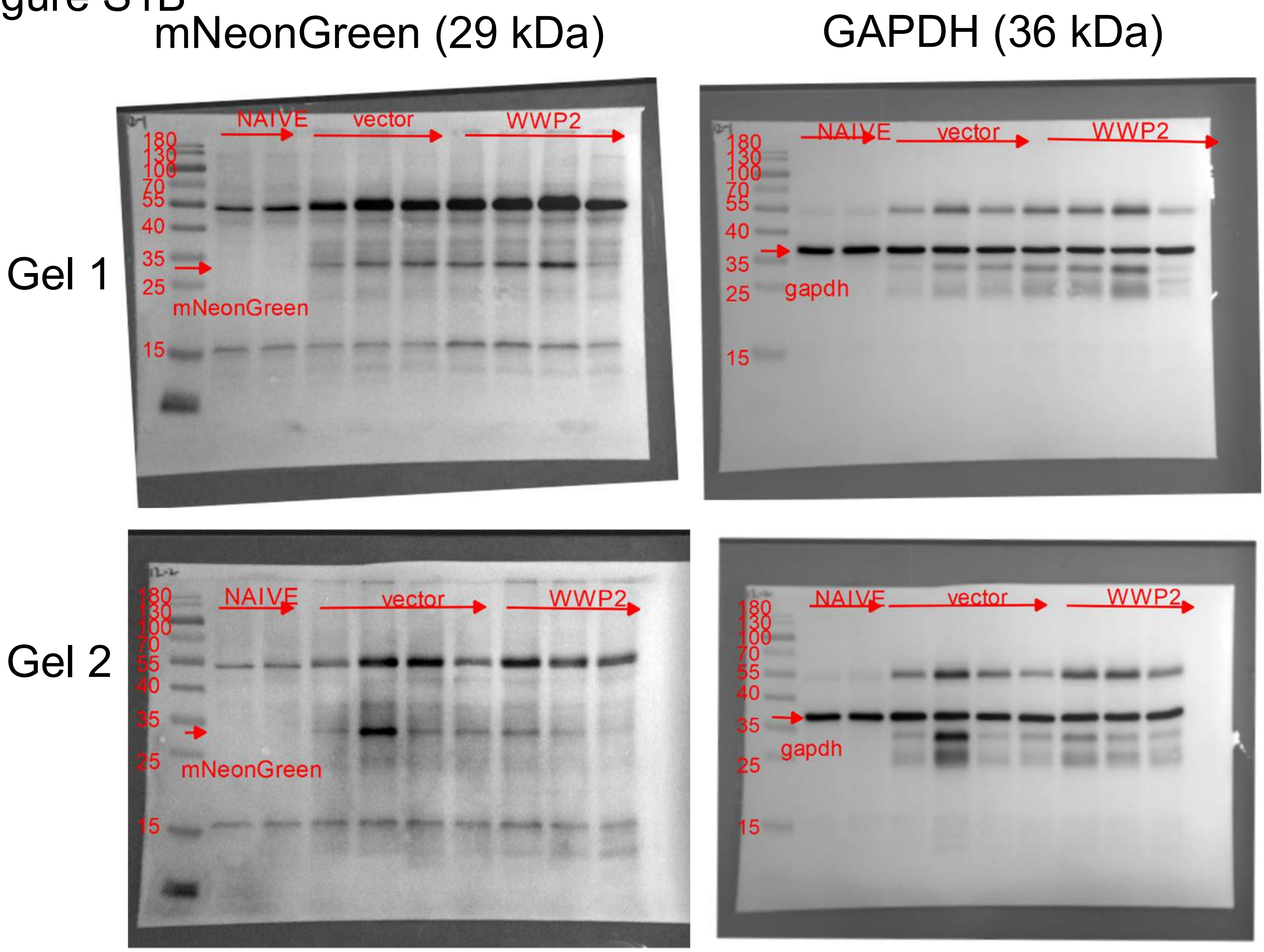

mNeonGreen (29 kDa)
GAPDH (36 kDa)
Gel 1
Gel 2
NAIVE
vector
WWP2
180
130
100
70
55
40
35
25
15
mNeonGreen
gapdh

Figure S1D&L

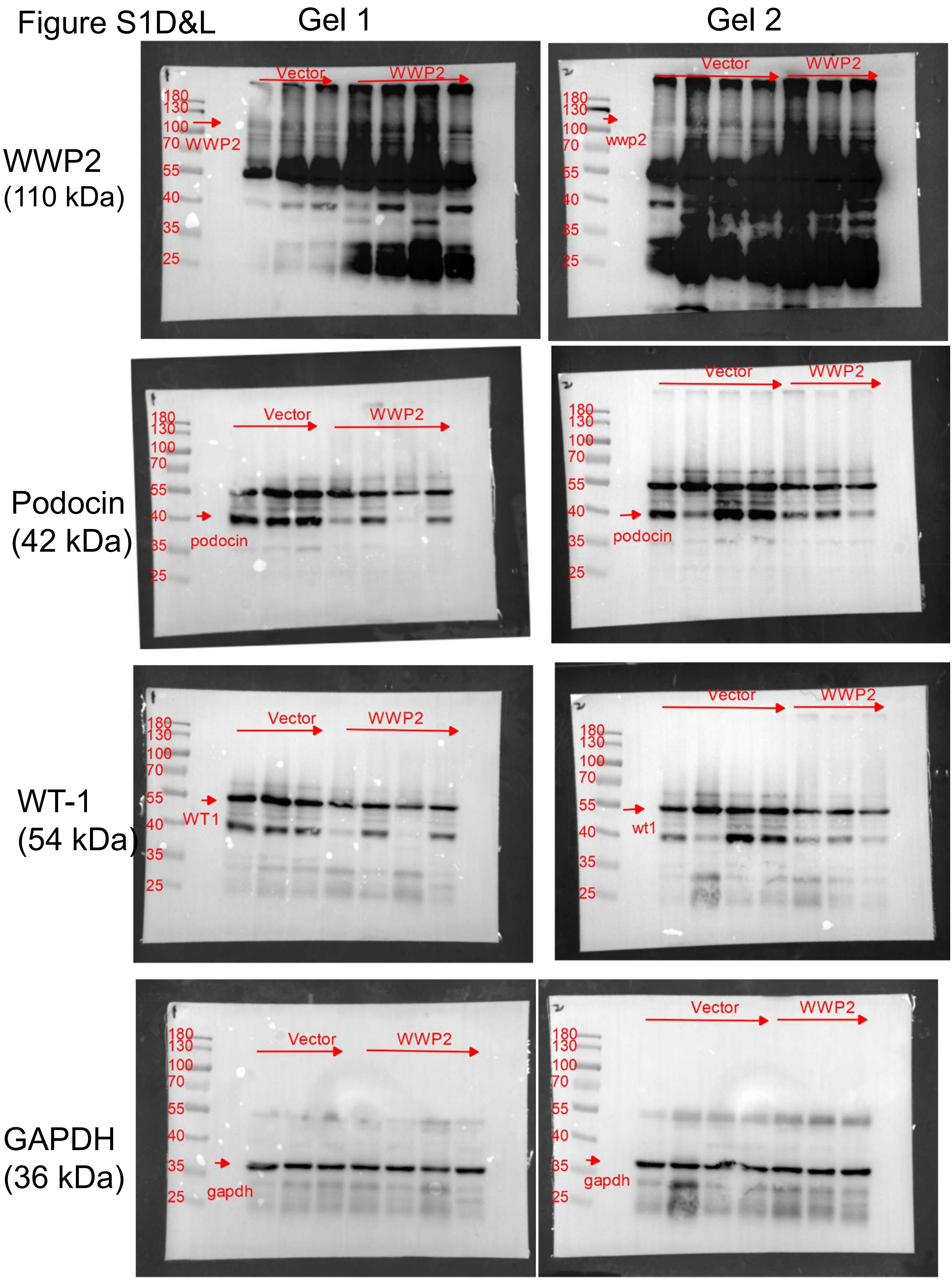

Gel 1
Gel 2
WWP2
(110 kDa)
Podocin
(42 kDa)
WT-1
(54 kDa)
GAPDH
(36 kDa)
Vector
WWP2
180
130
100
70
55
40
35
25
podocin
WT1
wt1
gapdh
wwp2

Figure S2A

NGAL (23 kDa)

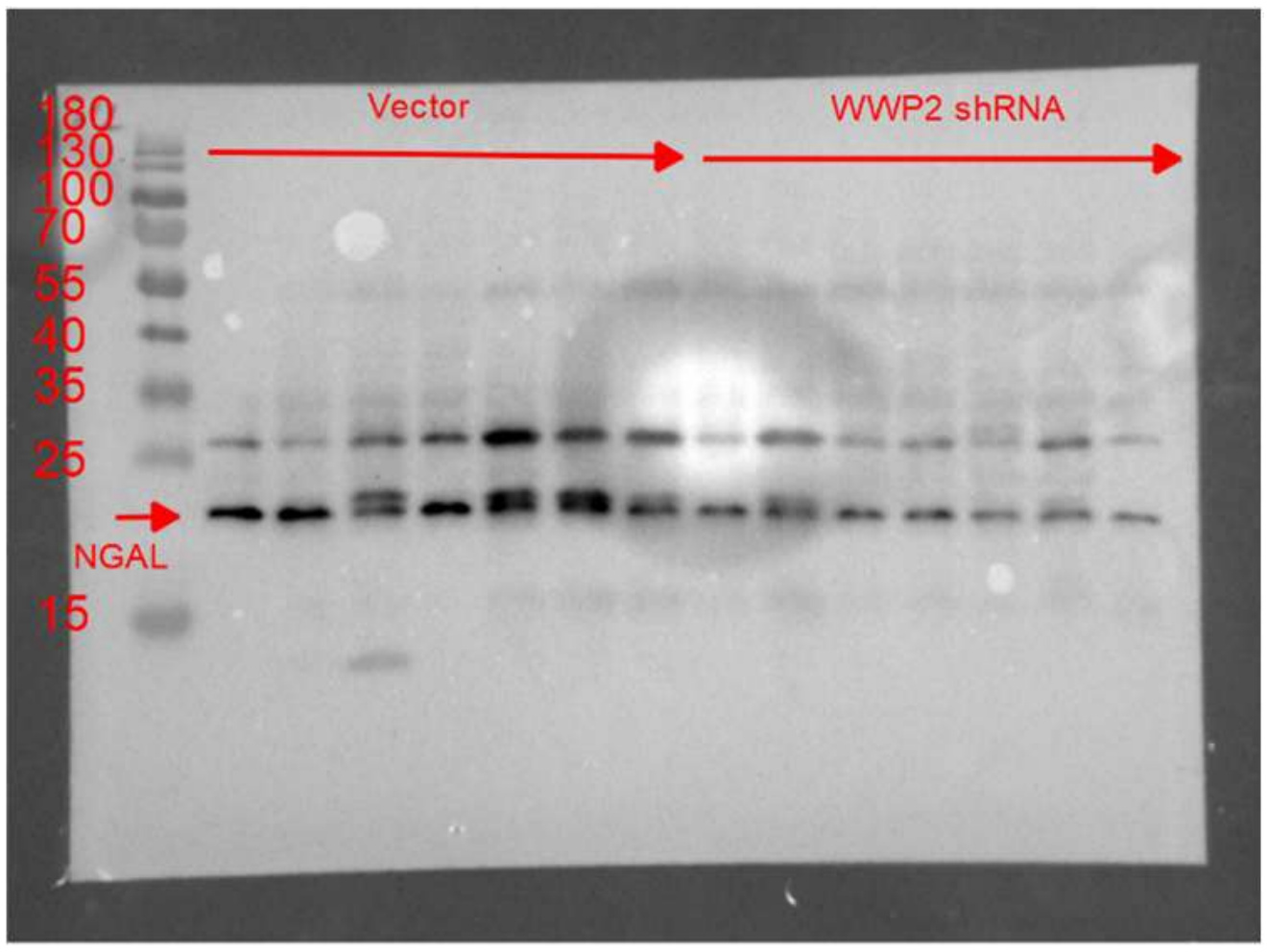

GAPDH (36 kDa)

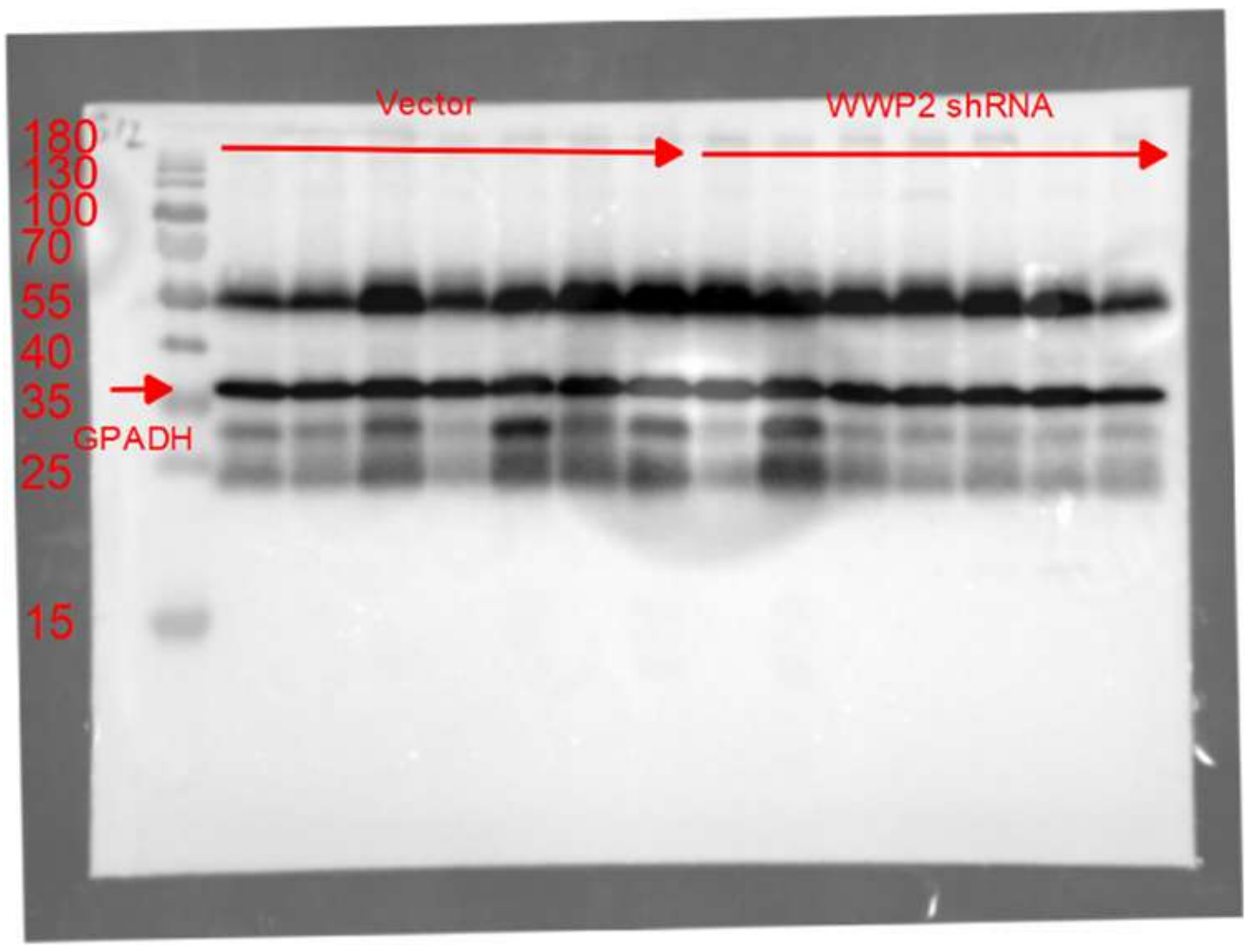

Figure S2D

NGAL (23 kDa)

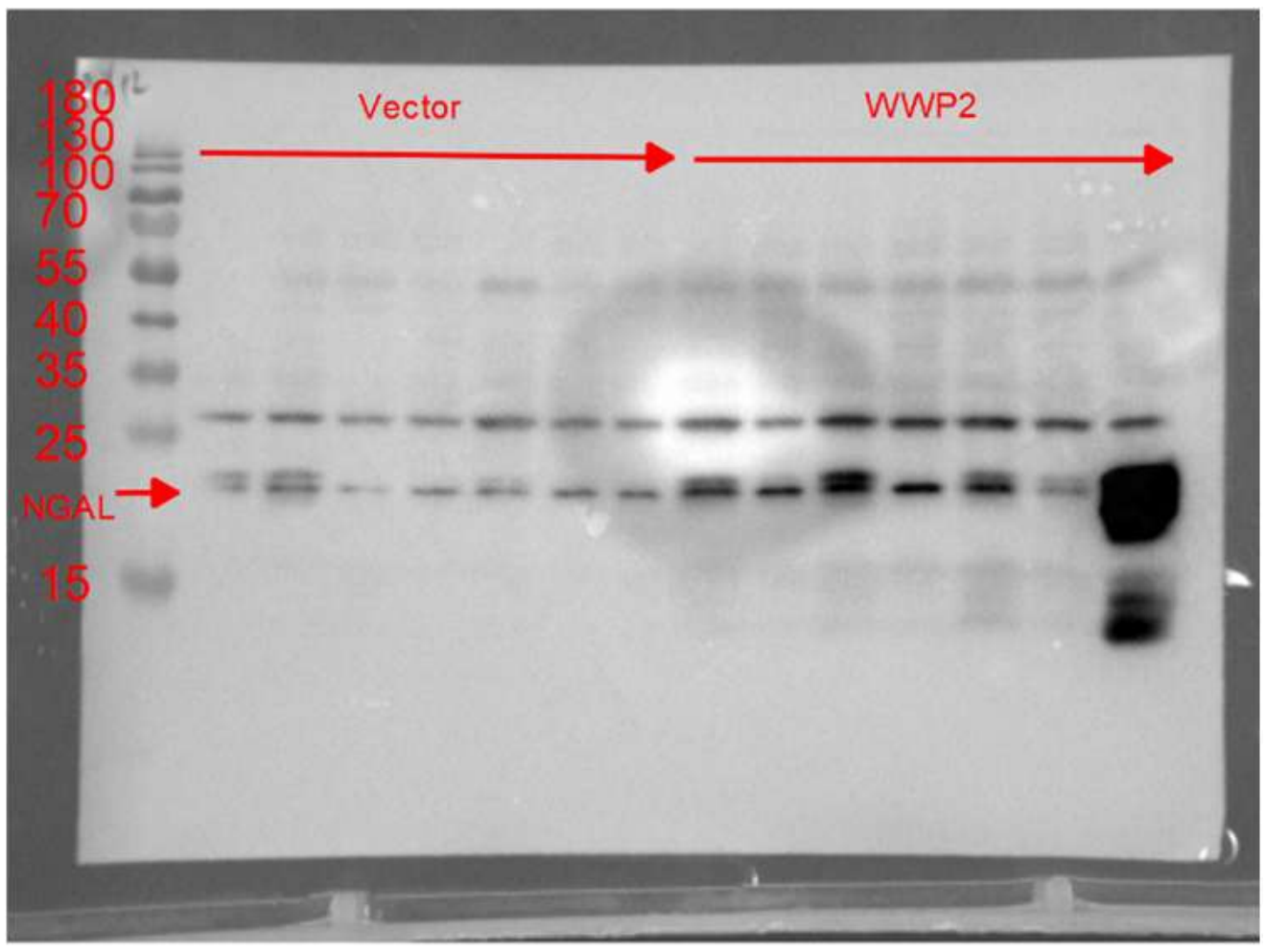

GAPDH (36 kDa)

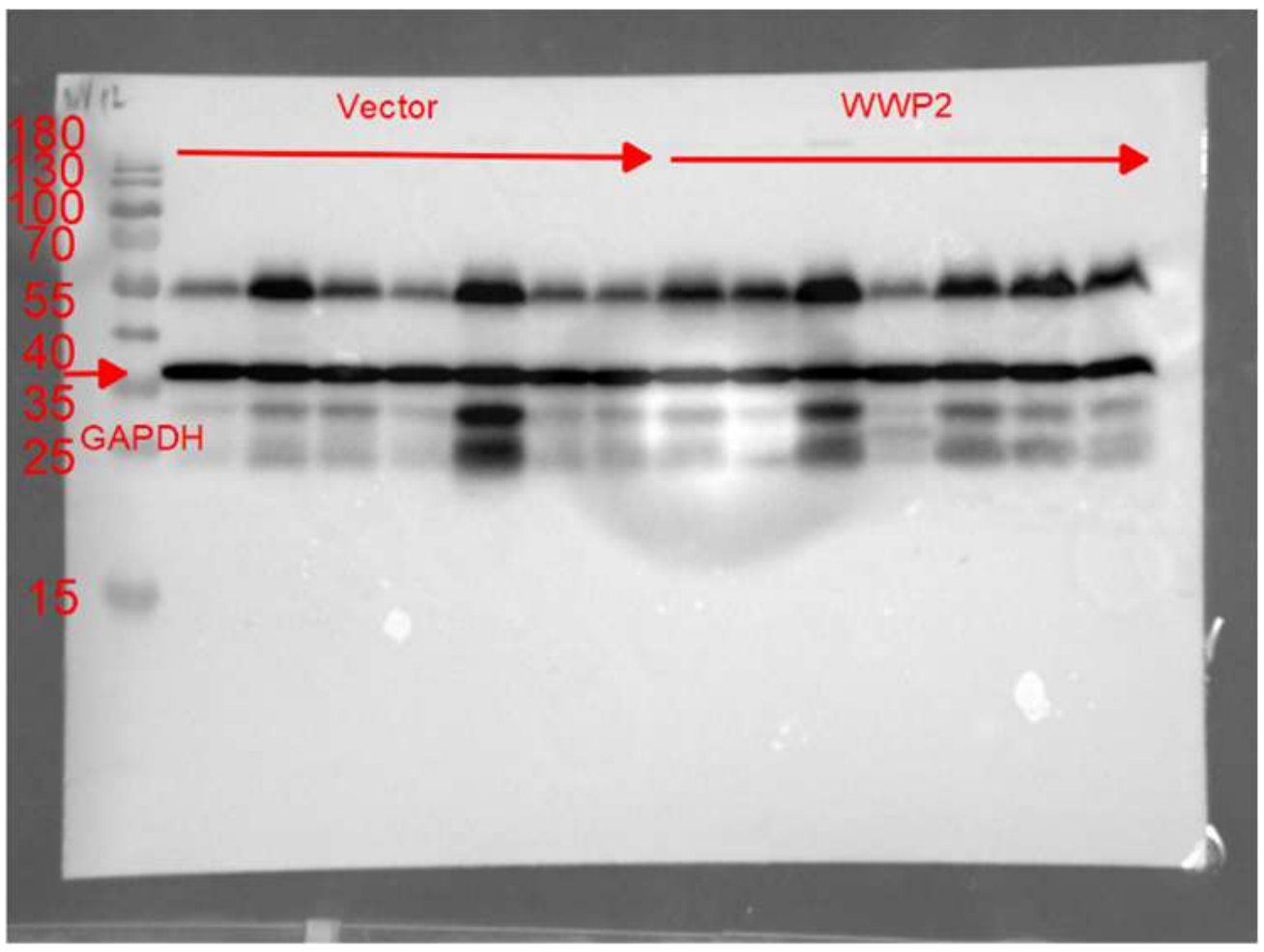

Figure S3A H2A (15 kDa)

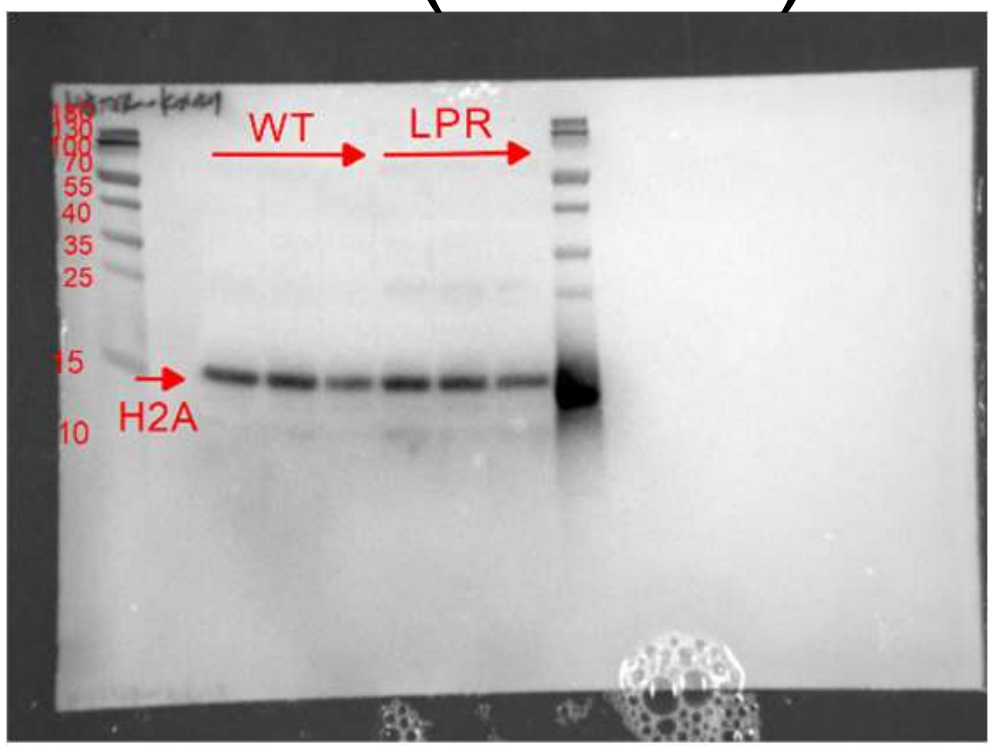

Ponceau S staining

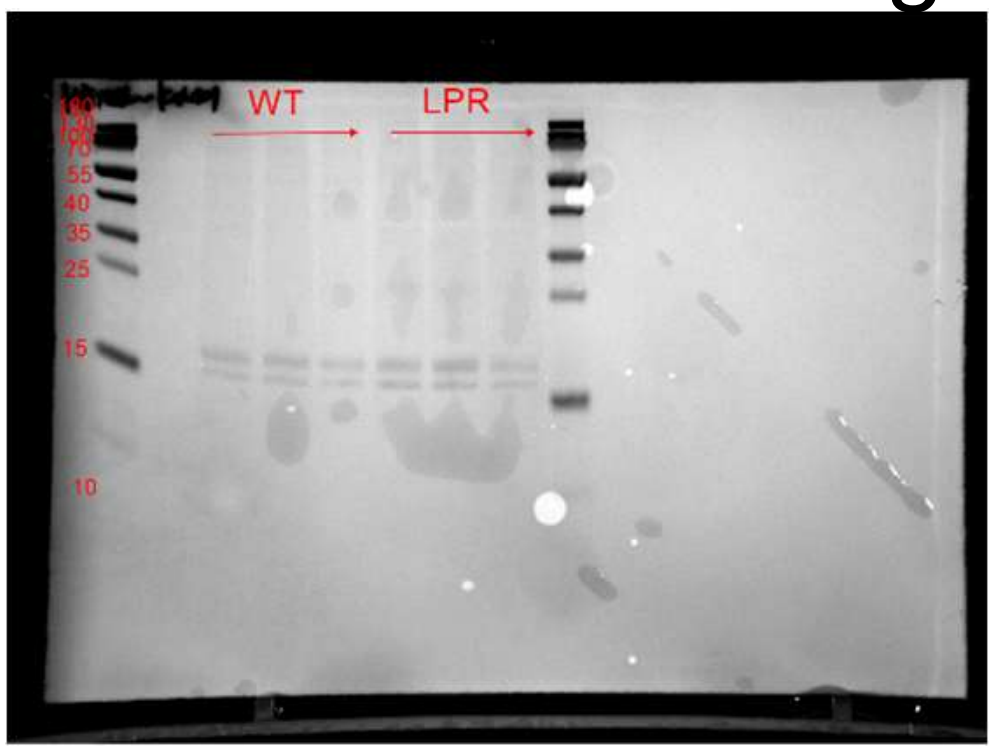

Figure S3C H2A (15 kDa)

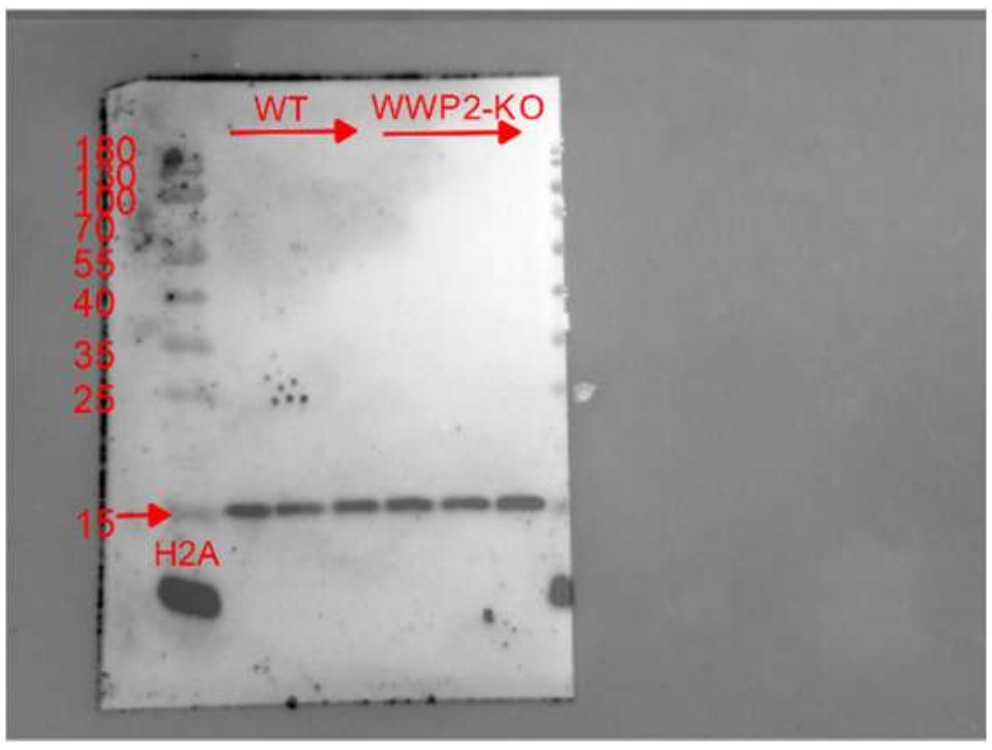

H3 (15 kDa)

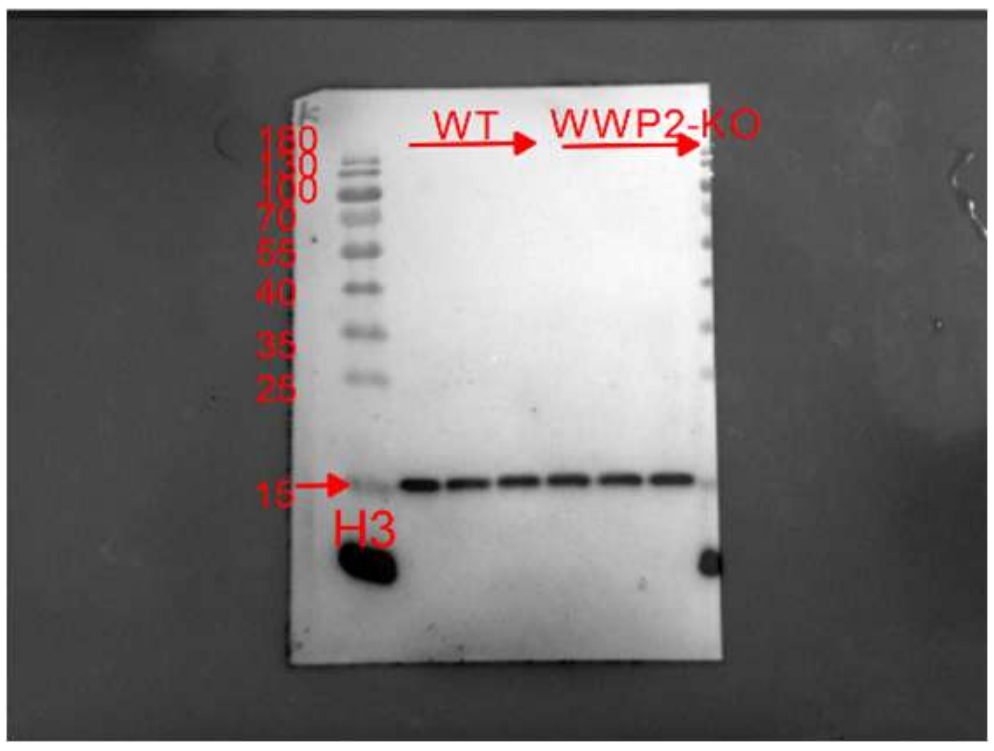

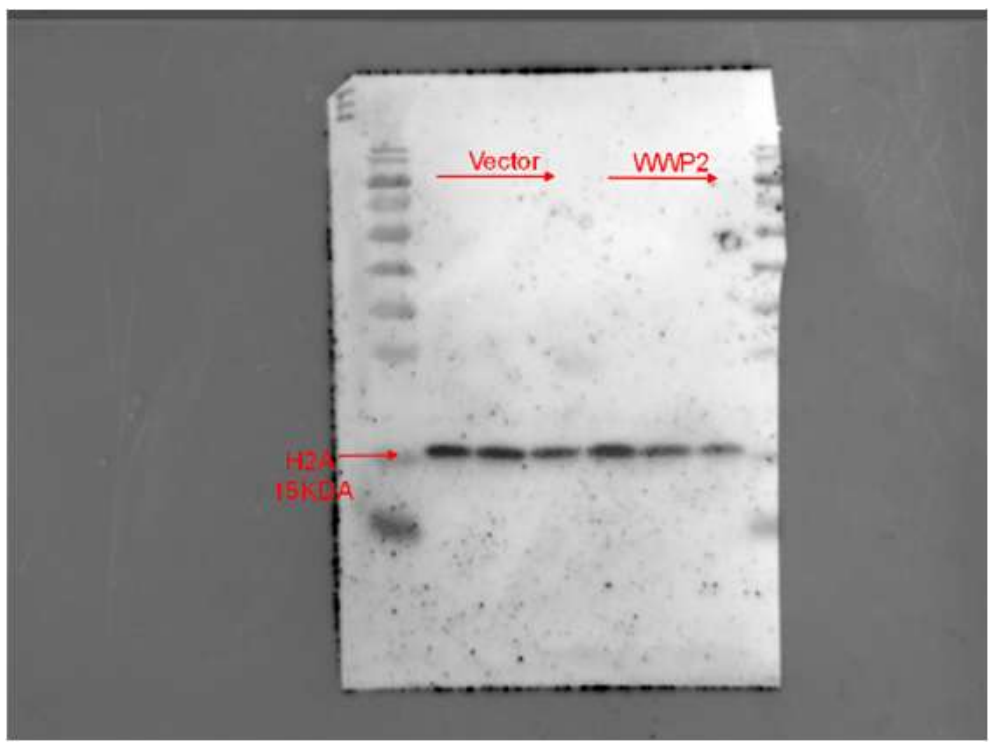

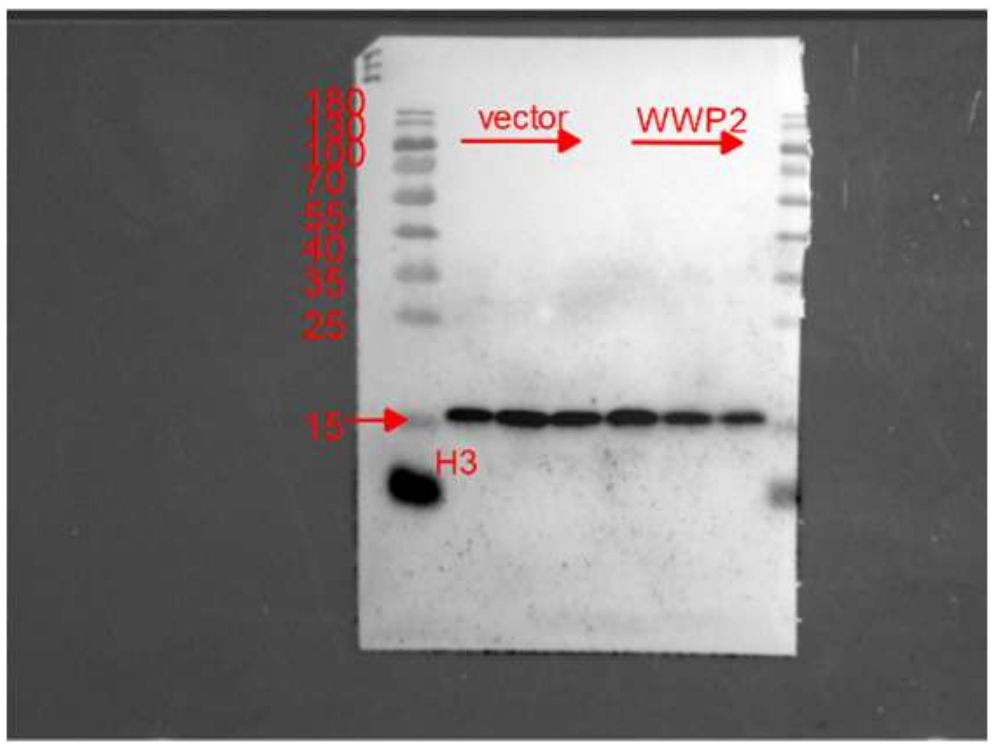

Figure S4

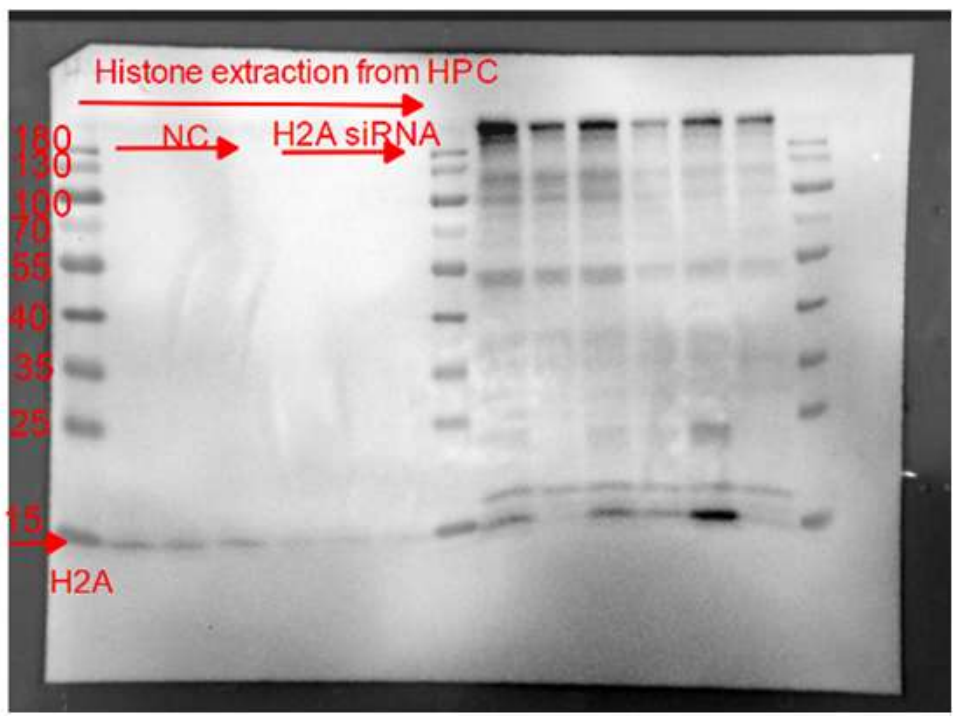

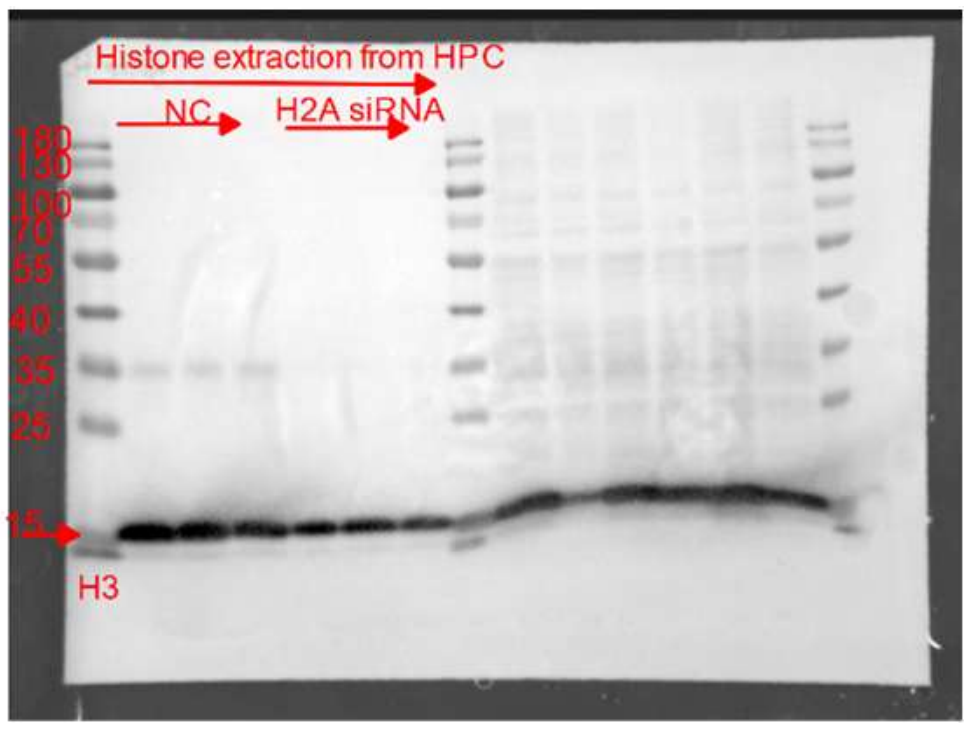

Supplement: Supplementary file 1 [file ijbsv22p6969s1.pdf]
